# Orogen-scale uplift in the central Italian Apennines drives episodic behaviour of earthquake faults

**DOI:** 10.1038/srep44858

**Published:** 2017-03-21

**Authors:** P. A. Cowie, R. J. Phillips, G. P. Roberts, K. McCaffrey, L. J. J. Zijerveld, L. C. Gregory, J. Faure Walker, L. N. J. Wedmore, T. J. Dunai, S. A. Binnie, S. P. H. T. Freeman, K. Wilcken, R. P. Shanks, R. S. Huismans, I. Papanikolaou, A. M. Michetti, M. Wilkinson

**Affiliations:** 1 University of Bergen, Bergen, Norway; 2 University of Leeds, Leeds, United Kingdom; 3 Birkbeck College, University of London, London, United Kingdom; 4 University of Durham, United Kingdom; 5 University College London, London, United Kingdom; 6 University of Cologne, Cologne, Germany; 7 University of Edinburgh, UK; 8 Scottish Universities Environmental Research Centre, East Kilbride, United Kingdom; 9 Australian Nuclear Science & Technology Organisation, Sydney, New South Wales, Australia; 10 Agricultural University of Athens, Athens, Greece; 11 Università degli Studi dell’Insubria, Como, Italy

## Abstract

Many areas of the Earth’s crust deform by distributed extensional faulting and complex fault interactions are often observed. Geodetic data generally indicate a simpler picture of continuum deformation over decades but relating this behaviour to earthquake occurrence over centuries, given numerous potentially active faults, remains a global problem in hazard assessment. We address this challenge for an array of seismogenic faults in the central Italian Apennines, where crustal extension and devastating earthquakes occur in response to regional surface uplift. We constrain fault slip-rates since ~18 ka using variations in cosmogenic ^36^Cl measured on bedrock scarps, mapped using LiDAR and ground penetrating radar, and compare these rates to those inferred from geodesy. The ^36^Cl data reveal that individual faults typically accumulate meters of displacement relatively rapidly over several thousand years, separated by similar length time intervals when slip-rates are much lower, and activity shifts between faults across strike. Our rates agree with continuum deformation rates when averaged over long spatial or temporal scales (10^4^ yr; 10^2^ km) but over shorter timescales most of the deformation may be accommodated by <30% of the across-strike fault array. We attribute the shifts in activity to temporal variations in the mechanical work of faulting.

Many areas of the Earth’s crust deform by distributed extensional faulting, not only in low-lying rift settings but also in areas of high topography^1^,^2^,^3^,^4^,^5^,^6^,^7^,^8^. Rather than being dominated by a single major plate boundary fault, these areas are characterised by numerous faults that accommodate the total strain, and the stress field in the seismogenic part of the crust varies significantly, both spatially and over time^1^,^2^,^5^,^6^. The consequence is that progressive loading of individual faults towards failure (earthquake rupture) is complex^6^ and this leads to large uncertainties in our assessment of earthquake hazard. Geodetic data, collected over the last few decades across the same areas, generally indicate a simpler picture of continuum deformation but unfortunately models that rely on these data to constrain loading rates on earthquake prone faults are often non-unique (e.g., refs 4 and 7). We show that new observational constraints on rates of fault slip over multiple earthquake cycles, interpreted within a geodynamic framework, can offer a fundamental advance in our understanding of the link between paleo-earthquake records, historical seismicity and geodetic measurements.

Across the central Italian Apennines (Fig. 1a) active extensional faulting is well documented, and there are clear correlations between regional extensional strain rates, elevated topography and active surface uplift of up to 1 mm/yr^4^,^7^,^8^,^9^ (Fig. 1b,c). However, the relationships between mapped faults, paleo-earthquake records, and interseismic strain accumulation are ambiguous^10^,^11^. The geodetically determined strain-rate field has been modelled assuming a homogeneous viscous lithospheric structure indicating a relatively simple relationship between gravitational potential energy (GPE) and smoothed decadal strain rates^4^. Viscous deformation occurs at depth (>15–18 km) where temperatures are higher, but nearer the surface seismogenic slip on fault planes dominates. Here we address the question of whether Holocene slip rates on faults within the central Apennines fault array are a passive marker of deeper viscous flow and, if not, what does control fault activity and earthquake recurrence ?

A key observation that helps us address this question is that there is a clear asymmetry in the distribution of historical seismicity since 1349 A. D. (Figure 1d). The 1349 A. D. earthquake sequence ruptured at least one fault on the southwest flank of the Apennines^12^, but since that time the spatial distribution of strong earthquake shaking is skewed towards the northeast flank of the long wavelength topography, including the 24th August 2016 M_w_6.2 and October 30^th^ M_w_6.6 events that ruined towns and villages around Amatrice and Norcia (in the Provinces of Rieti and Perugia). Some workers^7^ have therefore concluded that, based on historical records, many mapped faults on the southwest flank are no longer active and deformation is concentrated on faults to the northeast. However, the Holocene averaged extensional strain accumulation is distributed approximately symmetrically over both flanks (Fig. 1b) in contrast to the asymmetric pattern of strong earthquake shaking (Fig. 1d), suggesting that the historically-observed spatial distribution of large earthquakes may be a short-term feature. Vertical stress variations arising from dynamic support of the topography^13^ are unlikely to explain asymmetric seismic activity over this length scale. Here we present results of cosmogenic sampling of bedrock fault scarps along the southwest flank of the topographic high (Fig. 1a,c), which not only show that these faults have been active during the Holocene, but that slip-rates along individual faults vary over time scales of several thousand years, with quiescence on some faults in the southwest since 1349 A.D.

## Using cosmogenic radionuclides to constrain tectonic rates

Cosmogenic nuclides accumulate over time in the top few meters of the Earth surface, as a result of the interaction of cosmic rays with rock minerals, and are widely used to quantify rates of active geomorphic and tectonic processes^14^. Measurements of variations in cosmogenic ^36^Cl concentration along exhumed faults planes have been used to infer the timing of earthquakes on extensional faults (e.g., refs 15 and 16). However, identifying individual earthquake ruptures from these data has proved difficult, particularly at sites where geomorphic processes have also contributed to exhumation of the fault plane^17^. Here we use an alternative approach where we combine independent constraints on rates of Holocene fault slip (from offsets of a ~15 ka paleosurface mapped with LiDAR and ground penetrating radar (GPR), and constrained by geochronology and paleoclimate proxies) with a cosmogenic sampling strategy that captures both the exhumed and the pre-exhumation stage of fault slip by sampling the buried portion of each fault plane. This allows us to reconstruct the entire slip history for these faults since the demise of the Last Glacial Maximum (LGM; 12–18ka) and to test whether the inferred slip-rates deviate significantly over time from the rates implied by decadal geodetic measurements, thereby significantly improving our understanding of the underlying geodynamic controls on fault behaviour and seismic hazard.

**Figure 1:**
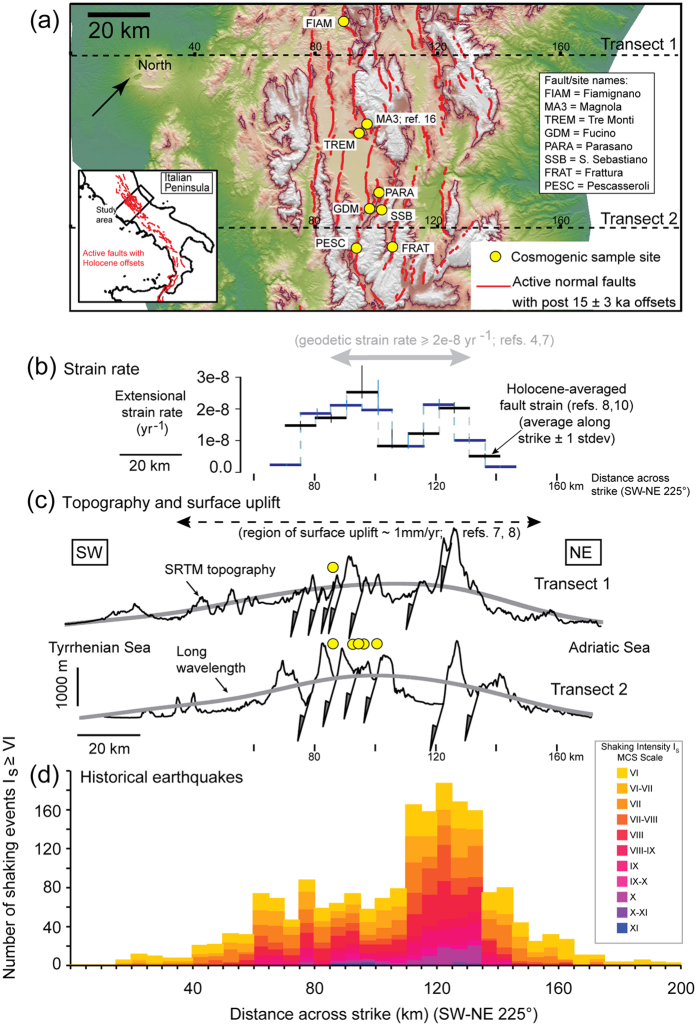
Location and regional setting of the Central Italian Apennines. (**a**) Sample sites (yellow circles; site MA3 described in ref. 16) located on a topographic map of the region using 10 m DEM (data source http://tinitaly.pi.ingv.it/download.html (described in ref. 34) plotted using ArcGIS 10.2-3 (www.ArcGIS.com)) (brown line marks 1000 m elevation contour). Inset indicates study area in central Italy; red lines are active faults. Across-strike variations (along transects oriented 225°) in (**b**) extensional deformation: geodetic rates (grey arrow from ref. 4) and strike-averaged Holocene rates (blue/black bars: two sets of 10km-wide transects offset by 5 km across strike to avoid sampling bias (from refs 8 and 10), (**c**) topography: long wavelength (grey line (from ref. 4)) and short-wavelength (black line (Shuttle Radar Topography Mission (SRTM) data from ref. 35); distribution of surface uplift (black dashed arrow; refs 7 and 8), (**d**) macroseismic shaking intensities (I_MCS_ ≥ VI; M ≥ 5.8) from 1350–2016 AD (see Methods and refs 32 and 33).

**Figure 2:**
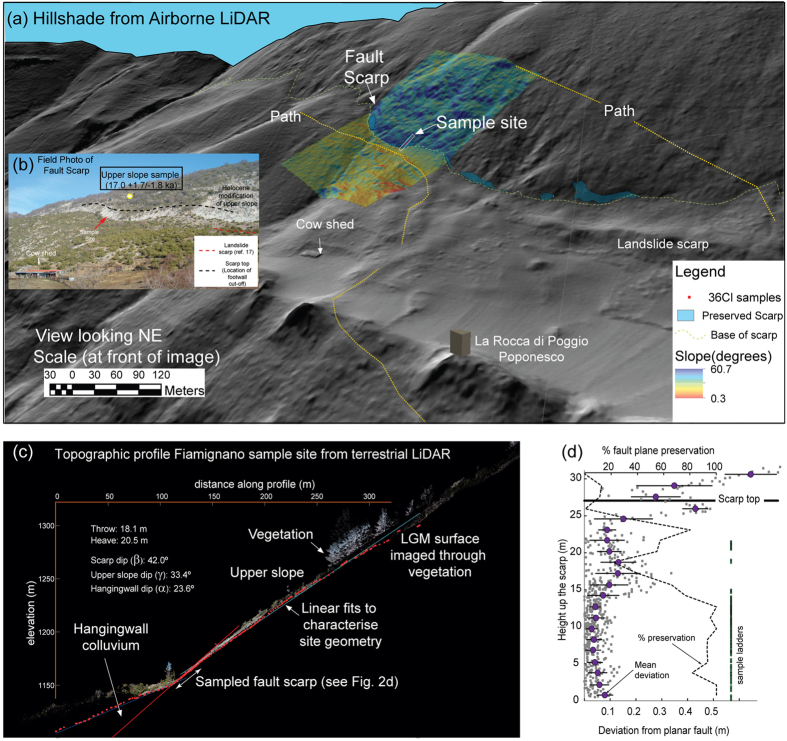
Scarp geometry and preservation along the Fiamignano fault. (**a**) Airborne LiDAR image showing along-strike continuity of the bedrock scarp (DEM generated from ALS data and co-visualised with slope data calculated using ArcGIS version 10.1 (http://www.esri.com/), (**b**) field photograph highlighting the sample locality away from areas of Holocene erosion (see also ref. 17), (**c**) and (**d**) LiDAR topographic profile (plotted using Riscan Pro version 1.2.1 b9 (http://www.riegl.com/products/software-packages/riscan-pro/)), site geometry parameters, fault plane surface roughness and % preservation used in the modelling of the ^36^Cl data at FIAM (see Methods Summary and Table S4.4.1).

**Figure 3:**
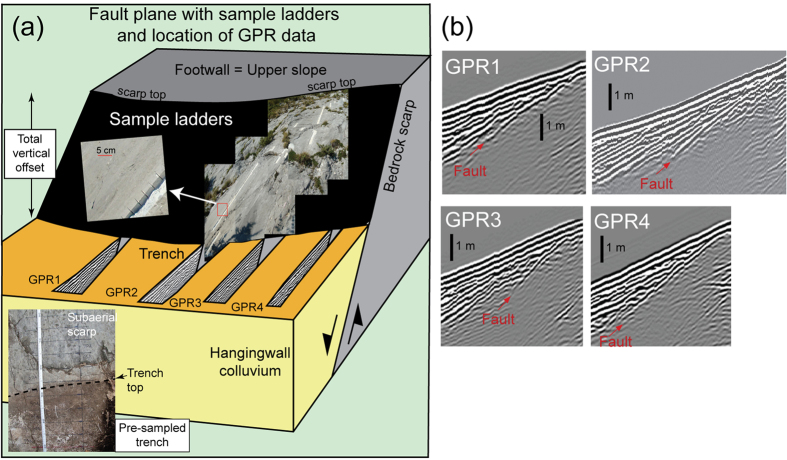
Sampling ladders and ground penetrating radar (GPR) images at site FIAM. (**a**) Detailed view of site and the sampling ladders showing location of GPR lines in the hanging-wall, (**b**) Four parallel GPR images showing undisturbed colluvial wedge and subsurface fault plane (plotted using Ekko View Deluxe 42 (https://www.sensoft.ca/products/ekko-project/overview/)). Sampling location indicated in Figs 1 and 2.

Along several large extensional faults (Fig. 1a) we sampled (by trenching) the portion of the fault plane not yet exhumed as well as the subaerial bedrock scarp as a function of increasing height (Figs 2 and 3). The scarps offset planar hillslopes preserved by the ten-fold reduction in erosion rate^18^ associated with the demise of the LGM ~15 ka^19^ (e.g., Fig. 2b,c). Each site consists of a striated fault plane, which we sample parallel to the slip vector, that becomes progressively rougher up dip (Figs 2d and 3a). Our methodology differs from that of previous workers in that we use LiDAR data to constrain the total post ~15 ka offset from displaced footwall and hanging-wall hillslopes (Fig. 2a,c), plus any variations in fault plane surface roughness (Fig. 2d), and we use these as independent constraints in the modelling of the cosmogenic data (see Methods and Supplementary Material). By utilizing both LiDAR and GPR data (Figs 2c and 3b) we select only those sites where Holocene geomorphic processes have not contributed to scarp formation or exhumation^17^. In particular, our GPR profiles and trenches reveal preserved (i.e., undisturbed) Holocene soil horizons and LGM stratigraphy on the hanging-wall side of the fault so that processes such as hill-slope erosion and landsliding can be ruled out (Fig. 3b). Our sample preparation and analytical approach follow published protocols^16^. We use a published Matlab^®^ code^16^ to model the measured ^36^Cl variations but we implement it in a Bayesian Markov Chain Monte Carlo (MCMC) modelling approach to obtain the best fit model for the full post-LGM slip history as well as to estimate confidence intervals on these fits. The novelty of using this Bayesian approach is that it does not require initial identification of slip events from subtle ^36^Cl variations^16^ and it allows data from independent sources (e.g., the timing of the demise of the LGM) to be used to constrain model fits in such a way that any uncertainty in these constraints is also taken into account.

## Evidence for fault slip-rate variations over time

**Figure 4:**
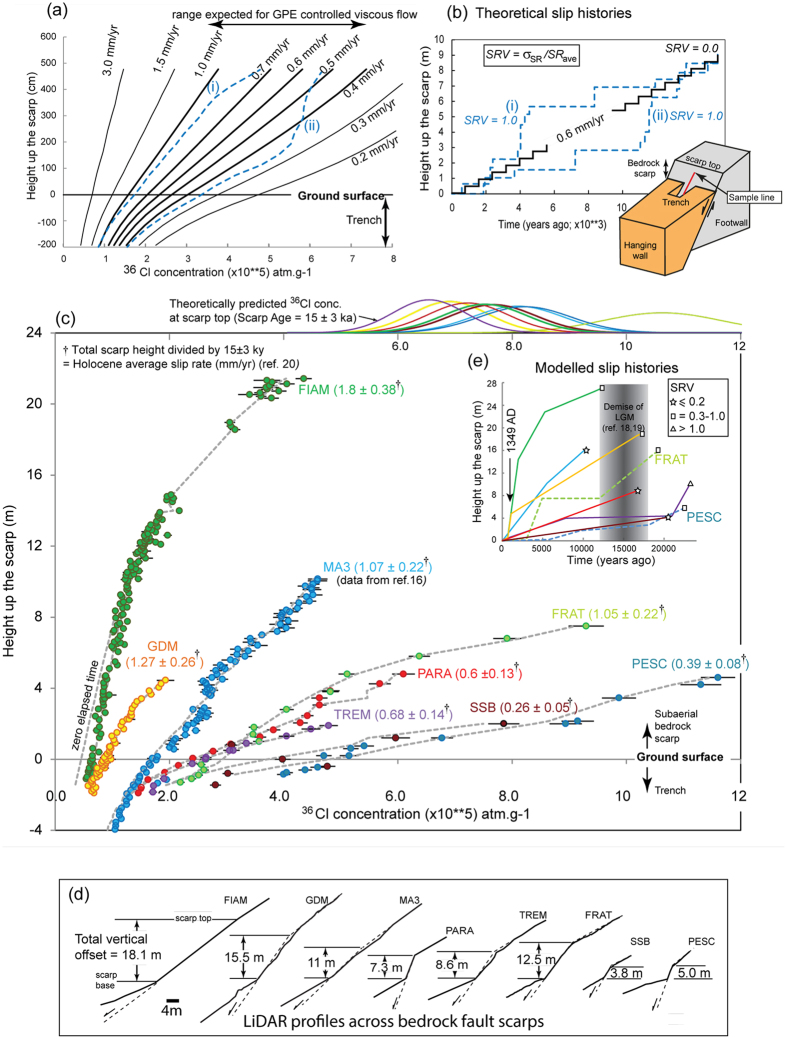
Theory, data and model results. (**a**) Variation in ^36^Cl concentration with sample height for constant slip-rate faults for a range of slip-rates (black); blue dashed lines: two notional cases of variable slip-rates (see Fig. 4b). Thicker lines in (**a**): range expected assuming distributed faulting (across *n* major faults, where 3 ≤ *n* ≤ 7) assuming the faults are passive markers of deeper viscous flow^4^,^9^. (**b**) Notional slip histories (i) and (ii) with SRV = 1(ref. 6). (**c**) ^36^Cl measurements from the eight sites; SR_ave_ in parentheses (locations in Fig. 1a; MA3 from ref. 16); grey dashed lines show highest likelihood fit for each site (Fig. 4(e)). Also shown for site FIAM is the fit for ‘zero elapsed time’; highest likelihood fit is for an elapsed time of ~665 years (i.e. AD 1349 from ref. 12) top axis in (**c**): predicted scarp-top ^36^Cl concentrations at each site assuming constant slip-rate since 15 ± 3 ka and zero inherited ^36^Cl. (**d**) LiDAR topographic profiles ordered left to right in same order as ^36^Cl data shown in (**c**), (**e**) highest likelihood slip histories for each data set (Figs S4.5.1–S4.5.10); symbols (star, square, triangle) denote SRV values and mark the top of the scarp. All analytical data (AMS and sample chemistry) are provided in the Supplementary Material Section 6.

The modelling of cosmogenic data along bedrock scarps involves a large number of parameters, many of which have associated uncertainties^16^, and excluding alternative exhumation scenarios can be challenging. However, our sampling strategy reveals a first order confirmation of theoretical predictions even for the un-modelled data (Fig. 4) and this greatly increases our level of confidence. Theory^16^ predicts that the overall increase in cosmogenic ^36^Cl concentration with height up a bedrock scarp should vary systematically with the average fault slip-rate (Fig. 4a). Where the fault plane is exhumed more slowly, i.e., a low slip-rate fault, the time the fault plane spends in the sub-surface cosmogenic production zone is longer and thus (i) the ^36^Cl concentration at the top of the trench, (ii) the rate of decrease in concentration with depth in trench and (iii) the rate of increase in ^36^Cl concentration with height on the scarp itself, should all be larger. Thus if faults slipping at different rates are plotted together, we expect an overall ‘fanning’ pattern of ^36^Cl profiles to be observed (Fig. 4a). To first order this is indeed the case (Fig. 4c). Because our approach already excludes geomorphic effects, deviations from this simple pattern must reflect either site specific cosmogenic production rates and/or temporal variations in fault slip-rate (e.g., Fig. 4b).

To further demonstrate the first-order agreement with theory (Fig. 4a), an independent estimate of the average Holocene slip-rate implied by these scarps can be obtained by dividing total scarp height at each site (Fig. 4d) by 15 ± 3 kyrs^20^. These rates, quoted in Fig. 4(c), show a variation between sites from ~0.3 mm/yr to ~1.8 mm/yr, consistent with the ‘fanning’ pattern of the ^36^Cl profiles. Furthermore, these rates (when corrected for fault dip) compare well with rates predicted by assuming that the total extension rate (3 mm/yr^7^) is uniformly distributed across strike (shared equally across several faults) (e.g., Figs 1c and 4a). Finally, the ^36^Cl concentrations in the top samples at 7 of the 8 sites are consistent (given that weathering precludes sampling the full height) with the maximum ^36^Cl concentration (Fig. 4c, top axis) predicted assuming each scarp formed at the average Holocene rate. These independent constraints strongly support a tectonic explanation for the observed ^36^Cl variations (Fig. 4c). In any case, alternative exhumation scenarios, such as landsliding (e.g., Fig. S4.5.3), cannot explain these data.

Using a Bayesian modelling approach, with site-specific parameterisations (Table S4.4.1) and whole rock sample chemistry ( Supplementary Materials: Table 6.1.0 and online data files), we then model the full temporal development of each scarp and thereby confirm our first order observations: the highest likelihood modelled slip histories for each of the eight ^36^Cl data sets (Fig. 4e) indicate that these bedrock scarps record cumulative fault slip on the southwest flank of the central Apennines since 17.8 ± 4.3 ka (average scarp age across all eight sites; Table S4.4.4), which overlaps with the demise of the LGM (12–18 ka) and an independent age estimate obtained by directly dating the preserved LGM hillslope (17.0 + 1.7/−1.8 ka; Fig. 2b). More importantly, however, our modelling also reveals that slip-rates have varied over time (Fig. 4e and Supplementary Material). As the periods of high slip-rate are not synchronous on all faults, a climate control on fault plane exhumation is not plausible.

Distributed extensional faulting across the central Apennines and the *average* Holocene rates (Fig. 4c) are consistent with bulk deformation that approximates a (viscous) continuum^4^,^9^, but marked changes in fault slip-rate during the Holocene, as indicated by the ^36^Cl data, are not. To evaluate this further we calculate slip-rate variability (SRV; ref. 6), which is the standard deviation of short term slip-rates, σ_SR_, divided by the long term average, SR_ave_ (e.g., Fig. 4b). Unless a strongly non-linear rheology is invoked, SR_ave_ is anticipated to differ between adjacent faults but SRV should be ≈0 and we can use our data to test this. A sliding time window of 3000 years is used to estimate short term rates (σ_SR_) and hence SRV based on our own sensitivity study ( Fig. S4.3) and previous work^6^. At 5 of the 8 sites presented here, we estimate SRV to be in the range 0.3–1.4 (Fig. 4e; Table S4.4.2). These temporal variations in slip-rate exceed the ±20% uncertainty on SR_ave_ associated with adopting an age range (15 ± 3 kyrs^20^) for the formation of the bedrock scarps since the demise of the LGM^18^,^19^, which sets a minimum magnitude of SRV ≥0.2 that we are confident can be distinguished from SRV = 0 ( Fig. S4.2.2). Our Bayesian modelling approach favours simpler slip histories and lower SRV values, thus slip histories characterised by SRV ≥0.2 must reflect significant temporal variations in slip rate over the Holocene. The robustness of our SRV estimates is further tested using synthetic cosmogenic data sets for different slip history scenarios (see Supplementary Material (Fig. S4.2.2)).

In summary, the cosmogenic data show that, since the demise of the LGM (12–18 ka), faults in the southwestern part of the central Apennines fault array have, over periods of several thousand years, slipped at rates significantly greater than the Holocene average rate while over other, similar length time intervals, these faults have been moving much more slowly or were temporarily quiescent. The overall summed across-strike strain-rate is maintained because when one fault slows another across strike becomes more active, e.g., sites PESC and FRAT (Fig. 4e) and quiescence in fault activity in the southwest since 1349 A. D., revealed at site FIAM, coincides with the focussing of historical earthquake activity in the northeast (Fig. 1d and ref. 7). Our main conclusion is that, whereas the decadal and Holocene-averaged extension rates in this area are consistent with continuum (viscous) deformation^4^,^9^, the millennial-scale behaviour of individual faults is more episodic, with elapsed times on some faults of several thousands of years^11^,^21^. The magnitude of the maximum slip-rates (SR_max_) that we infer from the ^36^Cl data (up to several mm/yr; Table S4.4.2) further imply that at any given time only a small fraction of the total fault population (≤30%; or ≤2 out of 6 faults across strike; Fig. 1c) takes up most of total regional extension.

## Geodynamic explanation

The periods of fault activity documented here are characterised by cumulative slip consistently larger in amplitude (many meters) than that generated by individual earthquakes and our field observations (Figs 2 and 3) exclude a geomorphic explanation. Maximum earthquake magnitudes in the Italian Apennines are in the range M 5.8–6.9 and generally produce average coseismic slip at the surface of 10’s of centimeters, rather than many meters^22^. During the periods of activity, the average earthquake recurrence must be relatively short (hundreds of years) to explain the higher than average slip rates that we observe (Fig. 4e). In contrast, the periods of quiescence that we infer are long (several thousands of years) compared to typical earthquake recurrence timescales in this area^23^ and instead relate to the migration of the locus of fault activity across strike. These characteristics reveal a spatial and temporal organisation to the active deformation that is at odds with the expected stochastic response of a heterogeneous elastic-brittle crust to distributed loading^5^. However, we can explain our observations if we consider the total energy dissipated during the formation of extensional faults in this tectonic setting.

**Figure 5:**
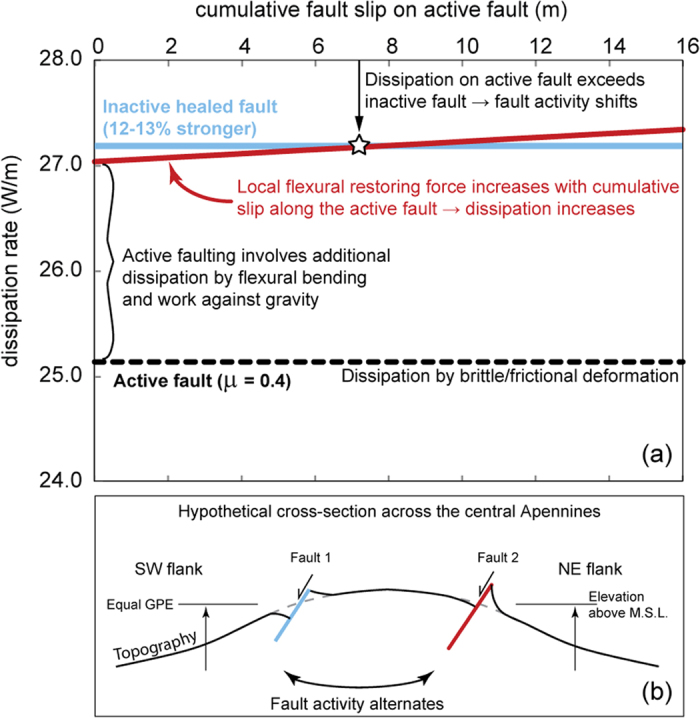
Geodynamic interpretation. Calculation of energy dissipation associated with normal faulting for a hypothetical case of two potential faults. Theory given in ref. 25 is modified to include variations in stress due to local flexural restoring force adjacent to the active fault^26^ (model parameters given in Table S4.6). Both faults are located at the same elevation above sea level so that viscous dissipation related to variations in GPE is the same. We ignore rock cohesion and deformation by pure shear. Flexural restoring forces during periods of time that one fault is active leads to an increase in the rate of work. In order to minimize energy dissipation in the system as a whole, the locus of activity will shift to an existing but inactive sub-parallel fault across strike. The abandonment of the active fault occurs only after several meters of cumulative slip, i.e., many individual slip events, hence the millennial timescale that we observe (Fig. 4e).

We apply dissipation analysis^24^,^25^ to the case of two normal faults at equal elevation located on either side of a high topography area so that viscous dissipation related to variations in GPE (e.g., ref. 4) is the same (Fig. 5). Strain weakening along faults localises deformation and reduces the rate of dissipation. However, as an extensional fault accumulates displacement, work is done against friction along the fault plane as well as by flexing fault-bounded crustal blocks and against gravity in generating footwall uplift^24^. The local flexural restoring force increases with cumulative slip along an active fault, increasing the rate of dissipation and hence resisting further motion (Fig. 5). Although the restoring force generated by meters of fault slip (e.g., Fig. 4e) is small (<1 MPa)^26^, comparable in magnitude to static stress changes that can be generated by nearby earthquakes^27^, it is the combination of flexure-induced stress variations and the accumulation of finite slip that progressively increases energy dissipation. Meanwhile, strength recovery (healing) increases the frictional strength of inactive faults (~12% increase with the parameters used in Fig. 5 (Table S4.6)). When the dissipation rate on the active fault exceeds that of a ‘healed’ inactive fault, the locus of activity can shift across strike (Fig. 5). Our interpretation does not preclude rupture of a previously quiescent fault if there is a sufficiently large stress increase following adjacent earthquake ruptures^27^, but for such a fault to become the locus for meters of further slip to accrue it needs to be one that is energetically favoured, i.e., lowest rate of total work. The mechanism we propose may be viewed qualitatively as analogous to the kinematic mechanism suggested to explain suppressed activity along faults in strike slip settings^28^, but in our example it is more appropriately ascribed to a flexural effect.

Finally, the dissipation analysis (Fig. 5) can reconcile evidence for regional deformation across the entire width of the central Apennines (Fig. 1b) with focussed historical earthquake activity (Fig. 1d). It implies that high strain rates (>1e-7 yr^−1^), currently confined to a zone only ~50 km wide on the northeast flank of the mountains, are the explanation for the skew in historical earthquake shaking and may even be interpreted as deformation associated with a ‘single’ fault system, as previous authors have suggested^7^. But our analysis also implies that this is a transient localisation phenomenon because in the past the zone of high strain rate was probably concentrated on the southwest flank. The viscous lower crust must be rather weak and characterised by a non-linear rheology for it to be able to accommodate localisation on this scale. Importantly, the cosmogenic data indicate that fault slip histories measured at the surface do not record a passive response to deep viscous flow but instead reflect interaction between brittle-frictional and viscous deformation processes. Finally, interpreting information about earthquake recurrence patterns on individual faults in this setting requires the migration of the locus of active deformation across strike to be taken into consideration.

## Conclusions

In summary, the ^36^Cl data reveal evidence for distributed deformation across both flanks of the central Italian Apennines but with significant temporal variability in fault slip-rates, and thus earthquake activity, that can be explained by the principal of minimum work. The implication is that the recent concentration of seismic activity on the northeast flank of the Apennines may persist for several thousand years but ultimately represents just one ‘snap-shot’ of a naturally complex deformational response to regional surface uplift that has, in the past, led to both flanks rupturing in major earthquakes. Slip-rate variability over multiple earthquake cycles can now be quantified and is essential to understand seismic hazard in areas of distributed extensional faulting because short term slip-rates, over the last few thousand years, can be significantly higher (and recurrence intervals much shorter) than both decadal (geodetic) and longer term geologic estimates may suggest.

## Methods

### Site selection, characterisation and sampling strategy

Detailed site characterisation (Figs 2 and 3) was undertaken to ensure that the fault surface was exposed only through tectonic exhumation (earthquake rupture) and did not include subsequent or contemporary geomorphic modification of the hanging wall, footwall or bedrock fault scarp. Sites were selected where the upper and lower slopes in the footwall and hanging wall of the fault plane were planar and free of Holocene hill-slope erosion and/or Holocene sedimentation^17^. The geomorphology of each site was assessed using LiDAR (terrestrial and airborne; Fig. 2a,c) and ground penetrating radar (GPR, Fig. 3). Terrestrial LiDAR was used to measure the 3D site geometry (Fig. 2c), the height of the bedrock scarp, and to assess fault plane surface roughness (Fig. 2d). Airborne LiDAR was used to assess the along-strike continuity of the scarps and preservation of the LGM paleosurface in the footwall and hanging wall of each fault (e.g., Fig. 2a). The GPR data image the hanging wall stratigraphy and were used to exclude geomorphic processes of fault plane exposure or burial in the Holocene by processes such as hill-slope erosion or landsliding (Fig. 3b). Weathering of the sampled fault plane is <1 mm, evidenced by preserved frictional wear striae. Structural data were collected at each site to determine the fault orientation and slip vector. Individual rectangular slabs of bedrock scarp were collected every 5 cm from the base of 1–2 m deep trenches up the fault plane, forming continuous sample ladders (e.g., Figs 2d and 3a) parallel to the slip vector. The trench part of each ^36^Cl profile strongly constrains the slip history and elapsed time (See Supplementary Material Fig. S4.2.3). Where an offset in the sample ladder was necessitated by incomplete fault plane preservation, two or more samples at the same height were taken from overlapping ladders. The integrated whole-soil bulk-density of the hanging wall colluvial wedge was calculated by determining the volume and weight of a sample from each soil horizon exposed in the trench. A bedrock sample collected from the planar upper-slope at FIAM (Fig. 2b) yielded a cosmogenic age of 17.0 + 1.7/−1.8 ka, which confirms the timing of the x10 drop in hillslope erosion rates (and the onset of scarp preservation) associated with the demise of the LGM^18^. Data tables and details of laboratory procedures are given in the Supplementary Material Section 2.

### Estimating fault slip-rates from the cosmogenic data

Preparation of *in situ*-produced cosmogenic ^36^Cl AMS (Accelerator Mass Spectrometry) targets from carbonate bedrock samples broadly followed the method in ref. 29, with subsequent AMS analyses according to ref. 30. The ^36^Cl data were then used to model fault slip histories by embedding the Matlab^®^ code developed in ref. 16 into a Bayesian MCMC parameter estimation framework to obtain the best-fit model and to estimate the uncertainties on our values for SR_ave_ and SRV for each site. The LiDAR and GPR datasets were used to constrain the site geometry parameters (Fig. 2c) and the whole rock chemistry of each sample is included (Supplementary Materials Table 6.1.0; full data files available online). Fault plane roughness variations measured using LiDAR were used to help define the heights of slip-rate change points (e.g., Fig. 2d). Rather than include an arbitrary pre-exposure correction^16^, we model the full height of the scarp at each site (Fig. 4d) by assuming that it is built by repeating earthquakes with magnitudes that are typical of Abruzzo (M 5.8–6.9), with appropriately scaled displacements based on ref. 31 (this approach is defined as ‘seismic pre-exposure’ in ref. 16). Slip-rate variations required to fit the ^36^Cl data are generated by increasing the number of earthquakes per unit time. Slip-rate variability, SRV (ref. 6), was calculated using a 3000 year sliding window. Full details of the modelling, the Bayesian implementation for each site, SRV calculations, sensitivity analyses, testing of alternative exhumation scenarios and results are given in the Supplementary Materials.

### Historical Seismicity

Historical records, consisting of macroseismic intensity measurements in individual settlements, were compiled from the Parametric Catalogue of Italian Earthquakes in the central Apennines from 1350–2016 (earthquakes from 1350–1997: refs 32 and 33). Intensity measurements less than VI on the Mercalli-Cancani-Seiberg (I_MCS_) scale and measurements caused by earthquakes with magnitudes less than 5.8 were removed (due to incomplete data for these events). The records were projected onto a transect orientated southwest-northeast (225°) perpendicular to the mean strike of faults in the central Apennines and plotted in 5 km bins along this transect (Fig. 1d). As the strongest intensity and highest density of macroseismic records occur in the immediate hangingwall of the fault that generated an earthquake, these historical records can be considered a proxy for the distribution of seismic moment release since 1350 A.D.

## Additional Information

**How to cite this article:** Cowie, P. A. *et al*. Orogen-scale uplift in the central Italian Apennines drives episodic behaviour of earthquake faults. *Sci. Rep.* **7**, 44858; doi: 10.1038/srep44858 (2017).

**Publisher's note:** Springer Nature remains neutral with regard to jurisdictional claims in published maps and institutional affiliations.

## Supplementary Material

Supplementary Material
