# Supplementary material for: Orogen-scale uplift in the central Italian Apennines drives episodic behaviour of earthquake faults
Source: Sci Rep. 2017 Mar 21;7:44858. doi: 10.1038/srep44858 (PMC5359594; doi:10.1038/srep44858)
Supplement: Supplementary Material [file srep44858-s1.pdf]

Supplementary materials

Orogen-scale uplift in the central Italian Apennines drives episodic behaviour of earthquake faults

Cowie P. A.[1], Phillips, R. J.[2], Roberts, G. P.[3], McCaffrey, K.[4], Zijerveld, L. J. J.[1], Gregory, L. C.[2], Faure Walker, J.[5], Wedmore, L.[5], Dunai, T. J.[6], Binnie, S. A.[6], Freeman, S.P. H. T.[7], Wilcken, K.[7,8], Shanks, R P.[7], Huismans, R. S.[1], Papanikolaou, I.[9], Michetti, A. M.[10], Wilkinson, M.[4].*

[1]University of Bergen, Bergen, Norway. (Previous address: University of Edinburgh, UK) *Patience.cowie@geo.uib.no (corresponding author)

[2]University of Leeds, Leeds, United Kingdom. R.J.Phillips@leeds.ac.uk; L.C.Gregory@leeds.ac.uk

[3]Birkbeck College, University of London, London, United Kingdom. Gerald.Roberts@ucl.ac.uk

[4]University of Durham, United Kingdom. k.j.w.mccaffrey@durham.ac.uk; maxwell.wilkinson@durham.ac.uk

[5]University College London, London, United Kingdom. j.faure-walker@ucl.ac.uk; l.wedmore.11@ucl.ac.uk

[6]University of Cologne, Cologne, Germany. tdunai@uni-koeln.de (T. Dunai also at University of Edinburgh, UK); sbinnie@uni-koeln.de

[7]Scottish Universities Environmental Research Centre, East Kilbride, United Kingdom. s.freeman@suerc.gla.ac.uk

[8]Australian Nuclear Science & Technology Organisation, Sydney, New South Wales, Australia. klausw@ansto.gov.au

[9]Agricultural University of Athens, Athens, Greece. i.pap@aua.gr

[10]Università degli Studi dell'Insubria, Como, Italy. alessandro.michetti@uninsubria.it

Availability of data and modelling code:

All analytical data (AMS and sample chemistry) for all samples are included in data tables in this document and are also available online at: https://www.dropbox.com/home/NatureScienceReports/data%20files%20as%20PDFs?preview=PDFs+of+all+data+txt+files.zip.
All of the the structural measurements, GPR and Lidar data that support the findings of this study are available from the co-authors upon reasonable request (KM, GR, JFW, LW), and ultimately will be part of the NERC data repository.
The Matlab® script used for the Bayesian implementation will be provided on request (PC). It relies on the code of Schlagenhauf et al. (2010) which is available as an online supplementary file (doi: 10.1111/j.1365-246X.2010.04622.x).

Table of Contents

1) Field surveying methods
1.1) Ground penetrating radar (GPR)
1.2) Airbourne LiDAR
1.3) Terrestrial LiDAR
1.4) Colluvial density estimation
2) ^{36}Cl Analysis: Sampling and Laboratory Methods
2.1) Fault plane sampling
2.2) ^{36}Cl sampling procedures and analyses
3) Site characterisation methods and individual site characterisation data
4) Modelling
4.1) Modelling methodology used for fault plane samples and sensitivity analysis
4.1.1 Methodology used for modelling upper slope sample at site FIAM (LGM inheritance)
4.1.2 Summary of 36Cl production rates used in the Schlagenhauf et al. (2010) Matlab code
4.2) Figures S 4.2.1 – S 4.2.3 Results of sensitivity analysis – site geometry parameters, slip rate variability (SRV) and elapsed time (ET)
4.3) Figure S 4.3 Test of Bayesian flexible change point approach using synthetic data
4.4) Tables S 4.4.1 – S 4.4.4 Summary tables (site specific parameters, Bayesian parameters and model results)
4.5) Figures S 4.5.1-S 4.5.10 Individual site specific model results in detail
4.6) Table S 4.6 Parameters used in dissipation analysis (Geodynamic explanation)
5) References
6) Data tables
All analytical data (AMS and sample chemistry) for all samples

1. Field surveying methods

1.1 Ground Penetrating Radar

For this study, we operated a Sensors and Software PE-100 GPR system in common offset profiling mode. For the acquisition of high-resolution data we used 200 MHz antenna with a separation distance of 0.5 m and a step size of 0.1 m. According to wave theory, the highest achievable vertical resolution of the survey is one quarter of the operating wavelength (Jol and Bristow, 2003). Using the setup described above we can calculate an ideal vertical resolution of 0.125 m (assuming an average pulse velocity of 0.1 m/ns and dry conditions). However, this value represents the best that can be achieved; in reality the resolution will be slightly less owing to the complexity of ground responses.

Raw radargrams were processed using a common workflow but with varying parameter values due to favourable conditions at all sites. Data processing included time-zero correction, de-wow filtering, bandpass filtering and automatic gain control application to boost signals at depth. To determine

average wave velocities a common mid-point (CMP) survey was conducted perpendicular to the survey lines and parallel to the fault planes. The results from these surveys give an approximate average pulse velocity of 0.1 m/ns, comparable to values obtained from the profile data using the shape of diffraction hyperbolae. This value was used to apply the topographic correction to the processed radargrams. Further details on the applications and processing procedures of GPR are presented by Neal (2004), Schrott and Sass (2008) and Jol and Bristow (2003). Data were plotted using Ekko View Deluxe 42 (https://www.sensoft.ca/products/ekko-project/overview/).

1.2 Airborne Laser Scanning (ALS)

Airborne laser scanning (ALS) is an active remote sensing technology that acquires 3D coordinates from the ground surface that can be used to generate automated digital terrain and surface models (Ackermann, 1999). Information on range, location and altitude of the target is generated in a 3D domain. The range information is determined by measuring the return time of the laser pulse from the surface to the aircraft mounted sensor (Flood & Gutelius 1997). Similarly, information on location is determined from ground based permanent or campaign GNSS control, and in-flight differential GNSS and inertial navigation systems (Wehr and Lohr, 1999).

For the present study, ALS data were captured using a Leica ALS 50-II system on board a Natural Environment Research Council's Airborne Research and Survey Facility Dornier 228-101 from an altitude of 18,000 ft. The Leica ALS 50-II is a discrete return system operating at a wavelength of 1064nm recording the first, second, third and last return and their intensity. This system has the ability to capture 83,000 pulses per second with a scan frequency of 45Hz, scan angle between 22 and 25º and beam divergence of 0.22mrad. In this study, the point density ranged between 0.9 returns/m2 and 1.2 returns/m2.

The initial stage of creating an elevation model from ALS is reassembling the data into separate return data layers using LAStools™ in ArcGIS 10.2™(http://www.esri.com/). In theory, the last returns represent ground surface, however, in the target area, a significant number of last returns came from forest canopy and low vegetation. The last return data was further filtered to remove vegetation using a Matlab™ Lowest_points script. An elevation model with a grid density of 1m was created using the nearest neighbourhood interpolation method in ArcGIS™ software from the filtered last return data. Subsequently, the elevation model was visualised as shaded relief model with standard ArcGIS™ software settings.

1.3 Terrestrial Laser Scanning (TLS)

A Riegl LMS z420i (RIEGL Laser Measurement Systems GmbH, Horn, Austria) was used to collect 38 laser scans during the period 06 April 2008 – 10 April, 2008 to characterise the surface topography at each of the 6 sample sites. The scanner has a nominal range of 800m and a specified range error of 5mm at 50m range (0.0001%). Each fault was scanned from an optimum viewing position located (147.8 to 661.1 m) from the fault on the hangingwall side. This gave a good definition of the footwall, scarp and hangingwall and enabled profiles to be created to determine the throw across the fault (e.g., Fig 2(c) in main text plotted using Riscan Pro version 1.2.1 b9 (http://www.riegl.com/products/software-packages/riscan-pro/). Point spacing is determined by the scanner step angle and varies uniformly with range. For the overview scans, point spacing varied from 3 mm closest to the scanner to 0.5 m at maximum range on the footwall with an average of

0.16 m on the fault scarp. Where possible, the faults were also scanned prior to sampling from a position close to each sample site (1-15m) to enable the quality of the fault plane to be recorded prior to the destructive sampling process. Here point spacing varied from 4mm at the base of the scarp to 20mm at the top of the highest scarp (at site FIAM shown in Fig 2 main text and Figure 4.5.2 in supplementary material).

The close up laser scans were filtered and cropped to remove vegetation. The roughness of the scarp (Fig. S 4.5.2) *Rq* was defined as

$$R_q = \sqrt{\frac{1}{n}\sum_{i=1}^{n} y_i}$$

where y_i is the distance of an individual point normal to an average plane through all points defining the fault surface.

At some sites (see details of individual sites in Section 3 below), a ruler survey method and clinometer measurements along the sample ladder were combined with the terrestrial LiDAR data to improve the detailed characterisation of the site geometry at both the meter scale and 10-100 m length scale.

1.4 Colluvial density estimation

Whole-soil bulk-density of hangingwall colluvium was calculated following the methods of Vincent & Chadwick (1994). Subsequent to trenching, soil horizons were identified and individual bulk-densities were determined for each horizon. An integrated bulk-density was then calculated for the entire sequence and this value was used in the Matlab® modelling. Density values for each site are given in Table S 4.4.1.

2. ^{36}Cl Analysis: Sampling and Laboratory Methods

2.1 Fault plane sampling

For fault plane samples, carbonate bedrock fault samples were collected in continuous sample 'ladders' from a single locality on each of the Pescasseroli, San Sebastiano, Gioia dei Marsi, Parasano, Frattura, Tre Monte and Fiamignano faults. Prior to sampling, each fault was examined along-strike for optimal characterisation, i.e. the exposed bedrock reflected only seismotectonic exhumation rather than modification through a geomorphic process such as erosion, sedimentation or gravitational sliding. Detailed site characteristics are provided in Section 3, with relevant surface and sub-surface survey methods outlined in Section 1.

Once a suitable fault plane site has been selected a trench is dug down to 1-2m and the hangingwall colluvium examined and whole-soil bulk-density calculated. Samples are then taken from the fault scarp by use of a hand-held angle grinder and diamond blade. A continuous ladder of rectangular blocks with dimensions of 15 cm wide, 5 cm high and 2.5 cm deep are cut from the base of the trench to the top of the preserved fault plane, following the down-dip direction of the striae. Overstepping of sample ladders is utilised to avoid isolated erosive features and fracture infill containing secondary calcite. Once cut, the samples are then numbered, described and

photographed before removal by use of a hammer and chisel. A sample numbering protocol is followed whereby the surface-rock interface at the top of the trench is set as the zero reference-frame.

For the upper slope sample at FIAM, the selected site was chosen on an elevated site on the footwall, away from the fault scarp. The sample site had a similar planar dip to the upper slope (c. 33°) and was approximately 0.5m above the footwall surface. Multiple 2.5cm deep samples were selected from the surface and analysed separately (see Section 3 and Table 6.1.8 for further data).

Finally, the sample site is characterised in terms of topographic shielding, elevation and geographic coordinates as described in Gosse and Phillips (2001).

2.2 ^{36}*Cl sampling procedures and analyses*

Prior to chemical digestion in the laboratory, the all individual samples are examined in detail for textural and lithological variability such as colour, entrained clasts within the breccia, presence of fine-grained clay cement and secondary calcite and/or iron-magnesium oxides. This information, coupled with separate chemical analyses, is used in conjunction with the 36Cl data to ensure that each sample is providing a robust measure of seismotectonic exhumation.

Preparation and chemical digestion broadly follows the methods outlined by Stone et al. (1996) and more recently in Schlagenhauf et al. (2010). After the removal of secondary calcite and/or metal oxides using a rock-cutting saw and handheld rotary tool, samples are crushed and an aliquot removed for PGNAA analysis. The remaining sample is then sieved to produce a 250–500 µm fraction for chemical processing. Multiple rinsing in 18 MΩ·cm H_2O and leaching in 0.33N nitric acid ensures removal of contamination by atmospheric chlorine within grain boundaries and defects. A 30 g fraction of leached sample is then used for further preparation. An aliquot preserved for whole rock chemical analysis via ICP-MS. After addition of an isotopically enriched $^{35/37}$Cl carrier, samples are fully digested in 2N nitric acid in an ice bath and any remaining residues are weighed so that precise total dissolved mass can be calculated. Following extraction of an aliquot for corroborative major element determination by ICP-OES, silver nitrate is added to precipitate silver chloride in darkroom conditions. In order to minimise isobaric interference from ^{36}S during accelerator mass spectrometry (AMS), the silver chloride precipitate is redissolved using an ammonium hydroxide solution and a barium nitrate solution is added. The sample is left for ~48 hours to promote precipitation of barium sulphate crystals. Samples are then passed through a 10µm Anatop syringe filter and the silver chloride re-precipitated. Further purification is achieved using multiple dissolution and re-precipitation steps, followed by water rinses of the precipitate using 18 MΩ·cm H_2O. After drying, the silver chloride precipitate is pressed into a silver bromide substrate within a copper cathode. Two reagent blanks are processed alongside each batch of 14 samples in order to trace Cl contamination during laboratory analysis. Typical blank $^{36/35}$Cl ratios are on the order of 10^{-15}.

Sample ^{36}Cl and natural-Cl concentrations are measured by acceleratory mass spectrometry (AMS) at the Scottish Universities Environmental Research Centre. AMS is ultrasensitive isotope-ratio mass spectrometry done at high ion energies to resolve molecule and atomic isobaric interference. The sputter ion source is operated for low source memory and gas-stripping is preferred for beam high brightness. 30 MeV $^{36}Cl^{5+}$ separation from ^{36}S isobar is wholly by efficient active ion-stopping measurement with a gas ionisation detector rather than involving passive post-stripping. Sample ^{36}Cl

is measured in ratio to sample ^{35}Cl and ^{37}Cl stable isotope signals in turn normalised to ^{36}Cl standards of natural stable-isotope ratio. The resulting twin ^{36}Cl/Cl values differ according to the relative amounts of natural-Cl and isotopically-adulterated carrier in the processed sample, enabling derivation of the former. Carrier relatively close to the natural stable-isotope abundance is preferred for consistent measurement (Wilcken et al, 2013). Data reduction accommodates the carrier ^{36}Cl content (Table 6.1.9).

Whole rock chemical analysis is undertaken to constrain low energy neutron production of ^{36}Cl on ^{35}Cl. Natural Cl concentrations within the rock were estimated from AMS determinations of ^{35}Cl/^{37}Cl. ^{36}Cl target elements (Ca, K, Fe, Ti) and a host of ancillary elements, are analysed following the procedures outlined in Schlagenhauf et al. (2010) for use in the Matlab® code. Tables 6.1.0 - 6.1.8 summarise the AMS and mean whole rock element compositions for each fault and the upper slope sample at Fiamignano. Table 6.1.9 show the Cl content and Cl isotopic composition of all processes blanks. Tables 6.2.x (available online) present the data in the format required by the Matlab® code used for modelling fault slip histories (Schlagenhauf et al., 2010).

3. Site Characterisation methods and individual site characterisation data

It is critical that fault plane sites studied with ^{36}Cl are fully characterized to show that exhumation from the ground is solely due to fault slip and not due to erosion or sedimentation processes. This supplement documents the geology and geomorphology of the sites in this paper, giving details of the variety of methods used for characterization. We provide two pages of figures for each site including the following datasets. Site parameters are given in Table S 4.4.1.

A) **Air photos and slope maps derived from LiDAR.** These data are needed to show that the fault scarp is continuous along strike for hundreds of metres along a geological fault that offsets mapped stratigraphy (Roberts and Michetti 2004). The fault scarp should be characterized by an exposed fault plane in bedrock limestones that offsets an upper slope and a lower slope that were originally continuous across the fault during the high erosion rate period of the last glacial maximum (LGM; Roberts and Michetti 2004). The images should demonstrate that no alluvial fans or colluvial fans exist that have been fed from incised gullies located above the chosen sample site because their presence would reveal exhumation/burial of the sample site by erosion/sedimentation processes. They should also demonstrate that the hangingwall and footwall cut-offs of the slope formed during the demise of the last glacial maximum are preserved as parallel lines, allowing the slip to be reconstructed back to that time if the slip vector is known (see below).

B) **Stereographic data recording the fault and slip geometry.** The slip vector orientation is needed to demonstrate that the slip can be reconstructed to guide the chosen orientation of sample transects for ^{36}Cl and the orientation of scarp profiles used to measure the offset of the slopes. The slip vector orientation is defined by the strike and dip of the fault, along with the plunge, and plunge-direction of any frictional wear-striae or corrugations on the fault plane. The preservation of millimetre-scale striae on the fault plane are also used prove minimal erosion of the fault plane after exhumation.

C) **Structural mapping along the strike of the fault.** Strike and dip data, along with plunge and plunge direction data for frictional wear striae and corrugations, must exhibit a consistent pattern along strike to show that the kinematics of the chosen sample site are not anomalous and hence un-representative of the exhumation of the fault scarp. These data

are displayed as graphs of these parameters as a function of distance along strike. The data show that the kinematics of the chosen sites are consistent along strike for hundreds of metres and hence the sample sites are representative of the scarps as a whole.

D) **Scarp profiles.** The amount of slip and hence offset of the slopes that formed during the demise of the LGM must be constrained because this is an input parameter for modeling the ^{36}Cl data. We have used airborne and terrestrial LiDAR supported by geomorphic observation in the field to define these offsets with cm-scale precision. We used the slip vector orientation to define the orientation of scarp profiles. We used geomorphic observations, observations in trenches, and ground penetrating radar (see below) to define the locations of the hangingwall and footwall cut-offs of the slope formed during the demise of the LGM. These cut-offs, alongside the dip of the fault measured from field structural measurements, are used to define the throw, heave and displacement.

E) **Ground penetrating radar.** The position of the hanging wall cut-off must be defined so that slip in the plane of the fault can be measured and used in ^{36}Cl modelling. We used geomorphic observations to try to identify sites where no hangingwall sedimentation or erosion have occurred to obscure or destroy the hanging wall cut-off (see above). We then checked our interpretations with ground penetrating radar to define the lateral continuity of sub-surface layers of sediment. Confirmation that the hangingwall cut-off is preserved and coincident with the ground surface defined by LiDAR is provided if the sedimentary layers prove to be parallel to the ground surface with no major discontinuities, continuing up to the position of the fault, as is the case with our sites. We check this further by excavating a trench and logging the trench walls to determine sub-surface layering (see below). The ground penetrating radar data also demonstrate the sites have not been affected by mass-movement/landsliding (Bubeck et al. 2015). The fault plane dip measured at the surface is plotted as a red arrow on the following figures to indicate the position and dip of the fault.

F) **Photos of the fault plane and sample locations.** We select sampling locations mainly based on the criteria listed above, but sites are prioritised if they also exhibit well-preserved fault planes. We select fault planes where we can prove minimal erosion after exhumation due to their smoothness and the preservation of millimeter-scale frictional wear striae produced at depth by frictional fault slip. We take samples from striated surfaces using a rock-saw, avoiding sites where the fault plane has been degraded by erosion (chemical dissolution, physical plucking - usually along fractures, and biological disturbance due to plants exploiting weaknesses in the fault planes). We also excavate trenches and sample fault plane beneath the ground to allow sample collection and hence measurements of the pre-exposure ^{36}Cl concentrations. The sub-surface fault planes are in places disturbed by fractures, but we avoid these whilst sampling, selecting surfaces with clear frictional wear striae where possible.

G) **Trenching excavation.** We excavate trenches to (i) check the subsurface layering and hangingwall cut-off location from ground penetrating radar and LiDAR, (ii) expose the fault plane for sub-surface sampling, and (iii) measure the density of the hangingwall material as this shields samples prior to excavation. The sites typically show 10-20 cm of organic rich soil that we assume is Holocene. Deeper material is usually scree with a fine matrix that contains markedly-less organic material – we assume this is colluvium deposited during the last glacial maximum. We correct the location of the hangingwall cut-off using the identified location of the Holocene to LGM sediment transition. We measure the density of the excavated material in trench because this is an input parameter for modeling the ^{36}Cl.

In summary, we take great care with sample site selection and characterisation because the exposure history and hence slip history is depends on selecting samples with well-defined exhumation solely by fault slip.

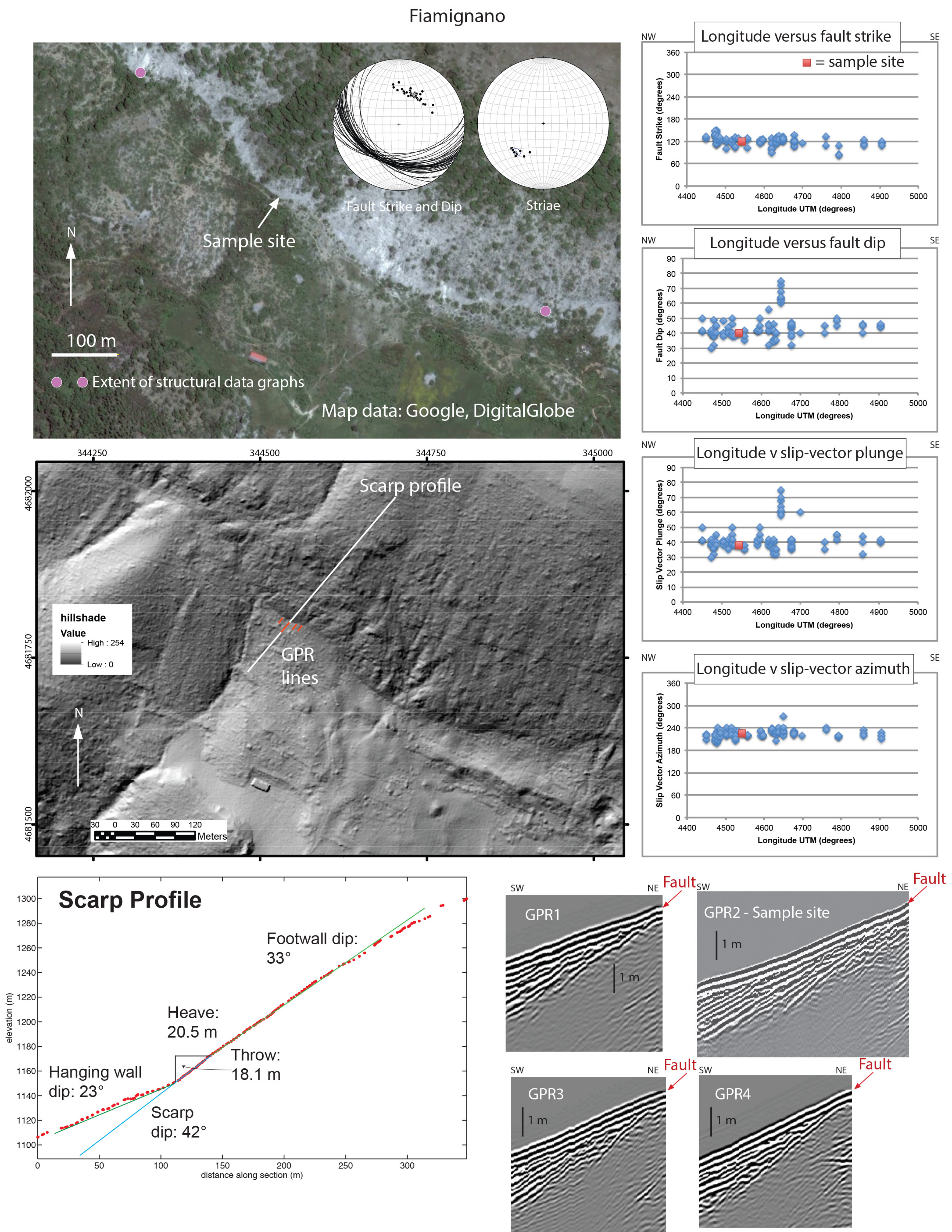
Fiamignano
Fault Strike and Dip
Striae
Sample site
N
100 m
Extent of structural data graphs
Map data: Google, DigitalGlobe
Longitude versus fault strike
= sample site
Fault Strike (degrees)
Longitude UTM (degrees)
NW
SE
Longitude versus fault dip
Fault Dip (degrees)
Longitude v slip-vector plunge
Slip Vector Plunge (degrees)
Longitude v slip-vector azimuth
Slip Vector Azimuth (degrees)
344250
344500
344750
345000
4682000
4681750
4681500
Scarp profile
hillshade
Value
High : 254
Low : 0
GPR lines
30 0 30 60 90 120
Meters
Scarp Profile
Footwall dip: 33°
Heave: 20.5 m
Throw: 18.1 m
Hanging wall dip: 23°
Scarp dip: 42°
elevation (m)
distance along section (m)
SW
NE
Fault
GPR1
GPR2 - Sample site
GPR3
GPR4
1 m

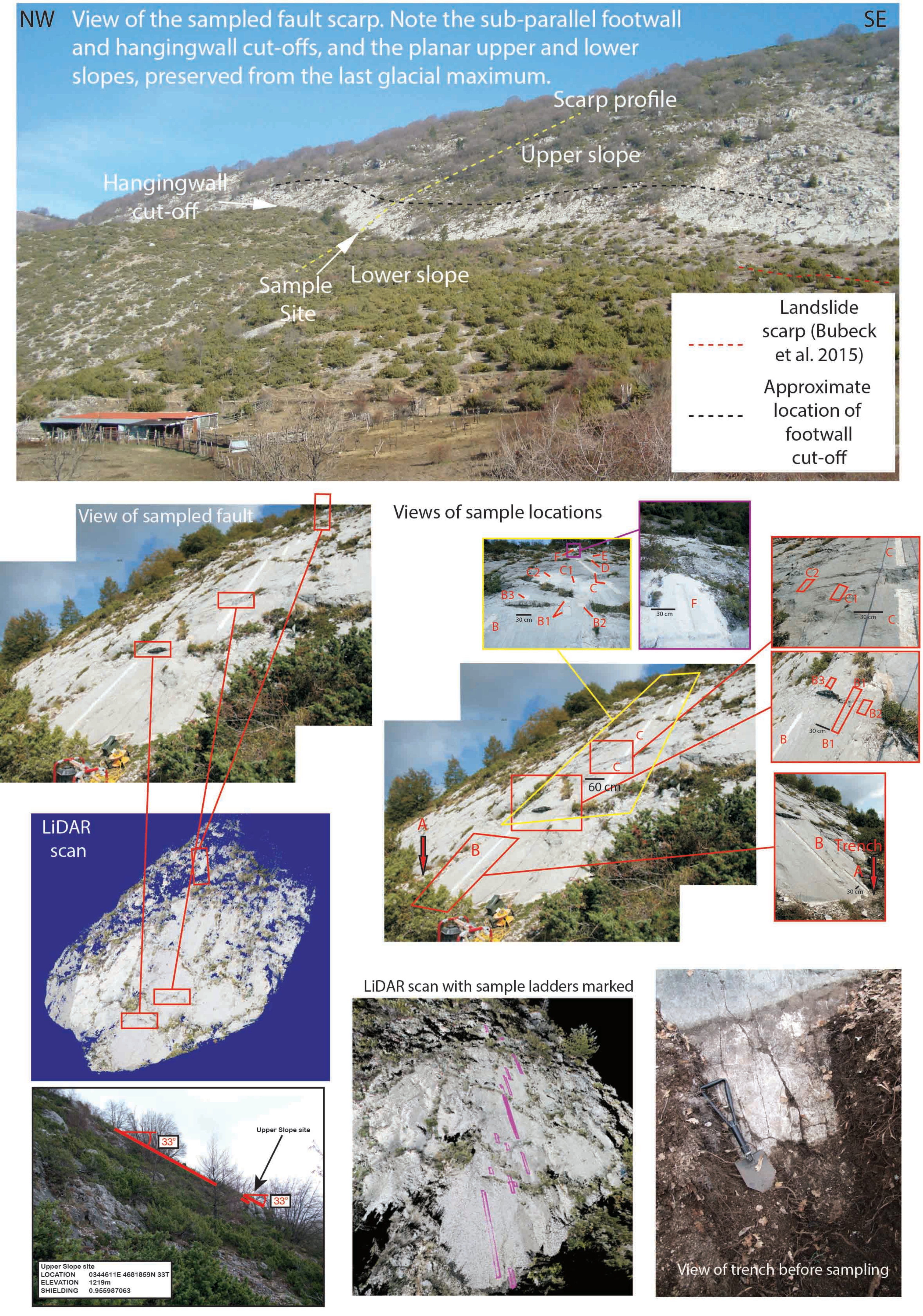
NW
View of the sampled fault scarp. Note the sub-parallel footwall and hangingwall cut-offs, and the planar upper and lower slopes, preserved from the last glacial maximum.
SE
Scarp profile
Upper slope
Hangingwall cut-off
Sample Site
Lower slope
Landslide scarp (Bubeck et al. 2015)
Approximate location of footwall cut-off
View of sampled fault
Views of sample locations
F
E
D
C2
C1
C
B3
B
30 cm
B1
B2
60 cm
A
Trench
LiDAR scan
33°
Upper Slope site
Upper Slope site
LOCATION 0344611E 4681859N 33T
ELEVATION 1219m
SHIELDING 0.955987063
LiDAR scan with sample ladders marked
View of trench before sampling

Frattura

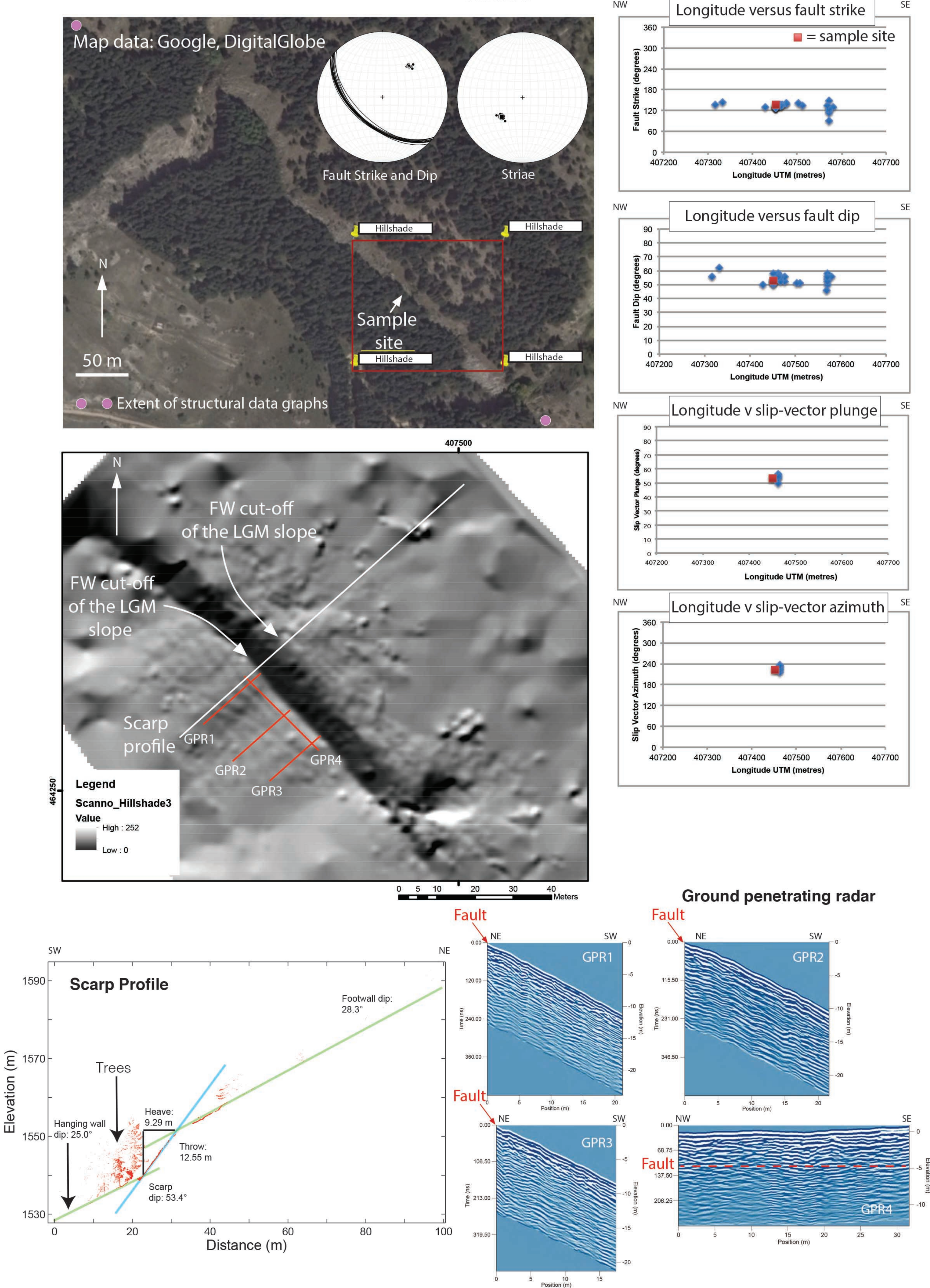

Map data: Google, DigitalGlobe
Fault Strike and Dip
Striae
Hillshade
Sample site
N
50 m
Extent of structural data graphs
Longitude versus fault strike
= sample site
Fault Strike (degrees)
Longitude UTM (metres)
Longitude versus fault dip
Fault Dip (degrees)
Longitude v slip-vector plunge
Slip Vector Plunge (degrees)
Longitude v slip-vector azimuth
Slip Vector Azimuth (degrees)
NW
SE
407500
464250
FW cut-off of the LGM slope
Scarp profile
GPR1
GPR2
GPR3
GPR4
Legend
Scanno_Hillshade3
Value
High : 252
Low : 0
Meters
Ground penetrating radar
Fault
NE
SW
Time (ns)
Elevation (m)
Position (m)
Scarp Profile
Footwall dip: 28.3°
Trees
Heave: 9.29 m
Throw: 12.55 m
Hanging wall dip: 25.0°
Scarp dip: 53.4°
Elevation (m)
Distance (m)

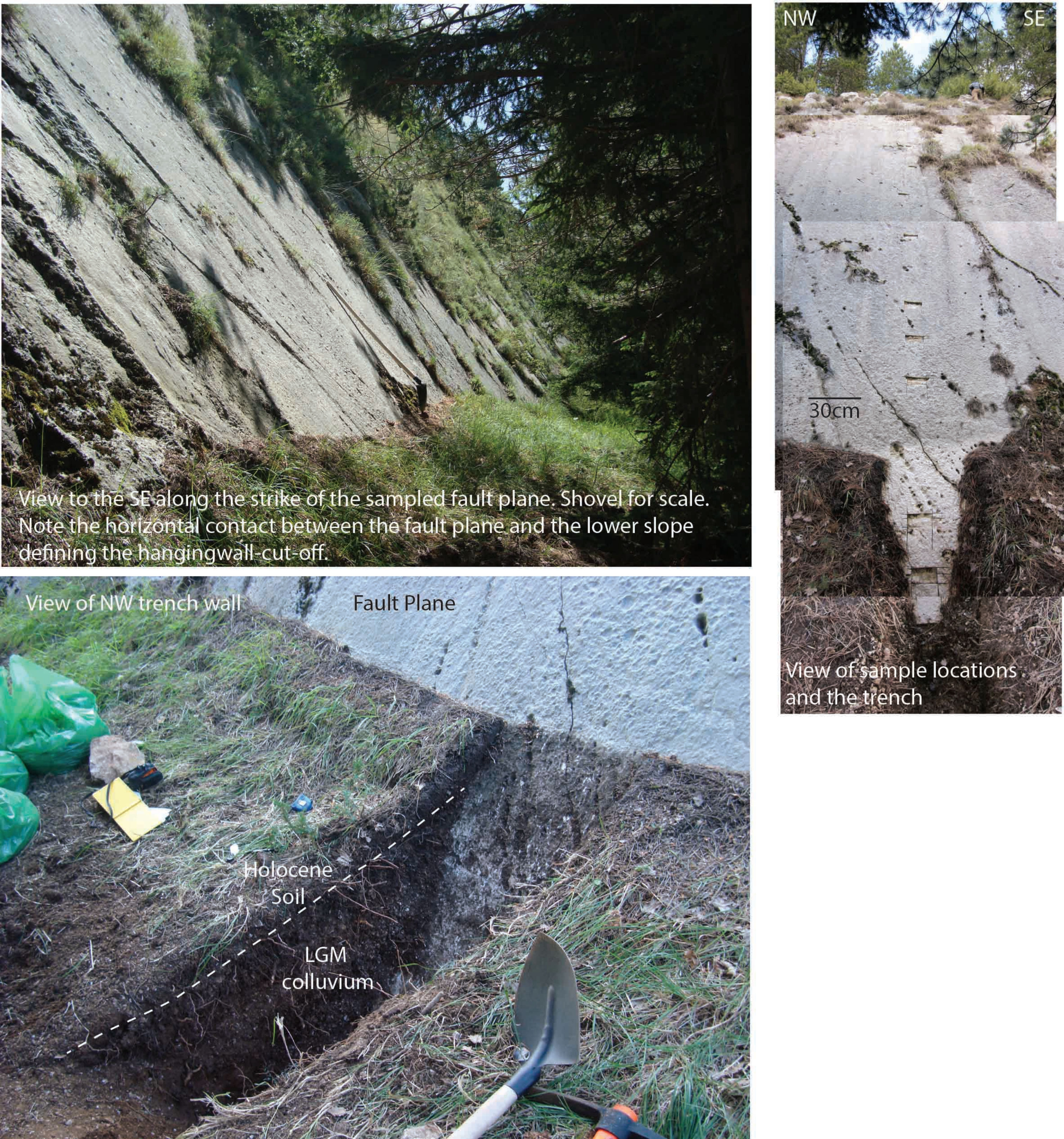
View to the SE along the strike of the sampled fault plane. Shovel for scale.
Note the horizontal contact between the fault plane and the lower slope
defining the hangingwall-cut-off.
NW
SE
30cm
View of sample locations
and the trench
View of NW trench wall
Fault Plane
Holocene
Soil
LGM
colluvium

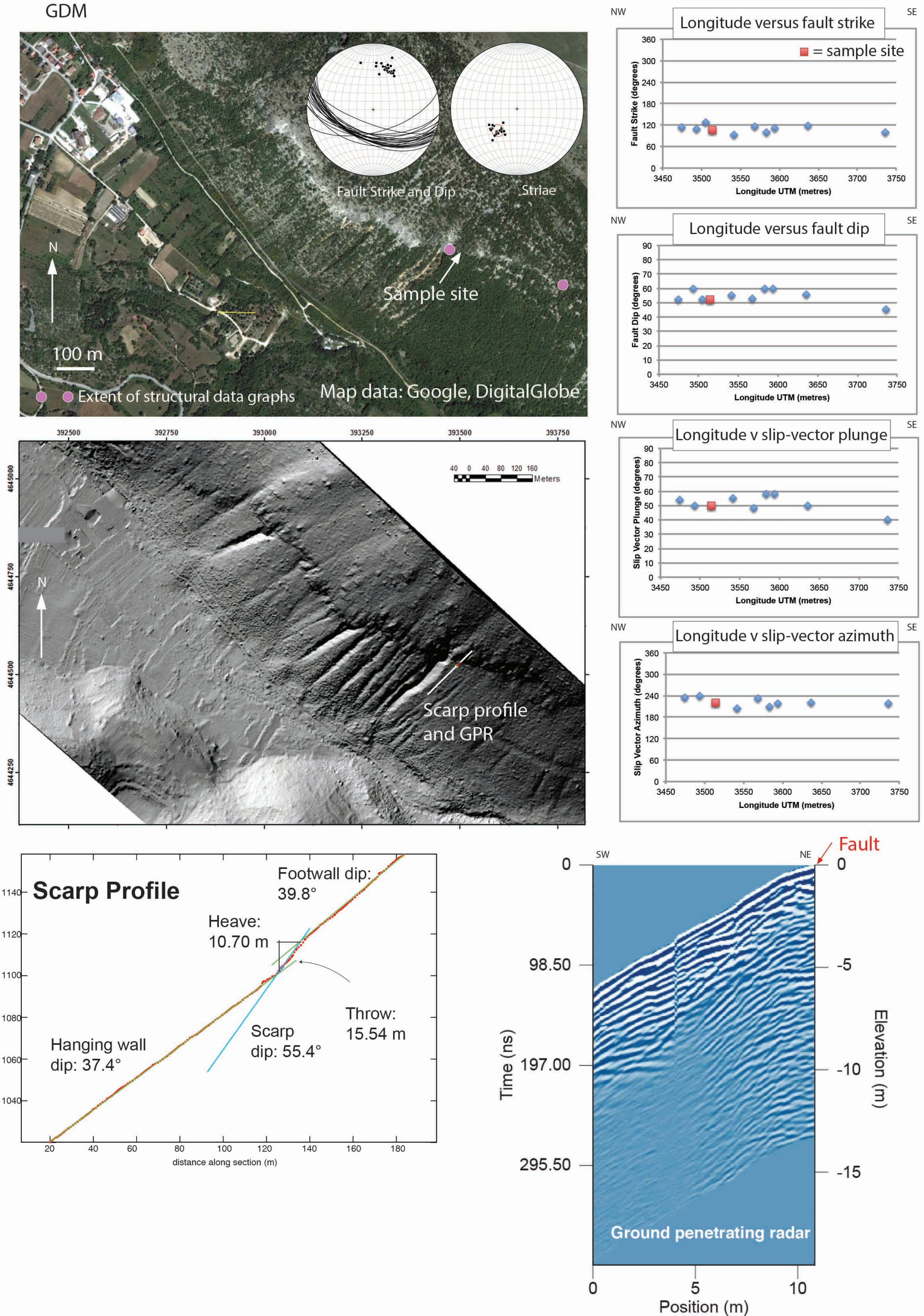
GDM
Fault Strike and Dip
Striae
N
Sample site
100 m
Extent of structural data graphs
Map data: Google, DigitalGlobe
NW
SE
Longitude versus fault strike
= sample site
Fault Strike (degrees)
Longitude UTM (metres)
Longitude versus fault dip
Fault Dip (degrees)
Longitude v slip-vector plunge
Slip Vector Plunge (degrees)
Longitude v slip-vector azimuth
Slip Vector Azimuth (degrees)
392500
392750
393000
393250
393500
393750
4645000
4644750
4644500
4644250
40 0 40 80 120 160
Meters
Scarp profile and GPR
Scarp Profile
Footwall dip: 39.8°
Heave: 10.70 m
Throw: 15.54 m
Scarp dip: 55.4°
Hanging wall dip: 37.4°
distance along section (m)
SW
NE
Fault
Time (ns)
Elevation (m)
Position (m)
Ground penetrating radar

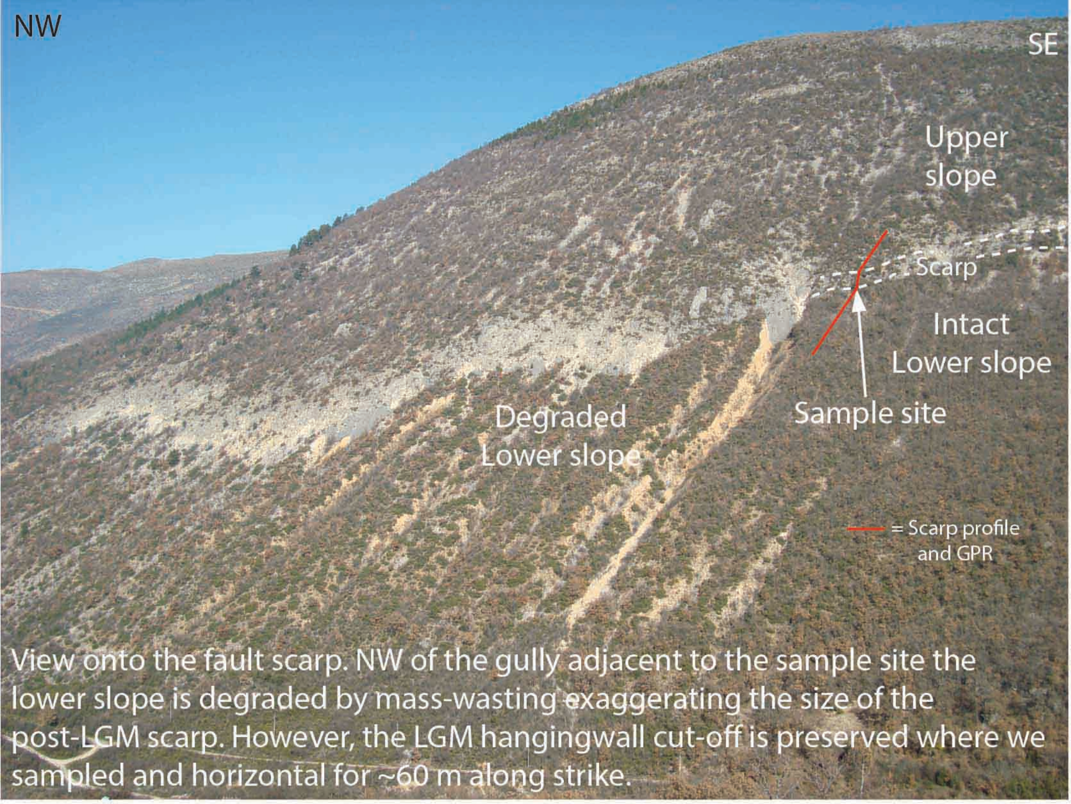
NW
SE
Upper slope
Scarp
Intact Lower slope
Sample site
Degraded Lower slope
= Scarp profile and GPR
View onto the fault scarp. NW of the gully adjacent to the sample site the lower slope is degraded by mass-wasting exaggerating the size of the post-LGM scarp. However, the LGM hangingwall cut-off is preserved where we sampled and horizontal for ~60 m along strike.

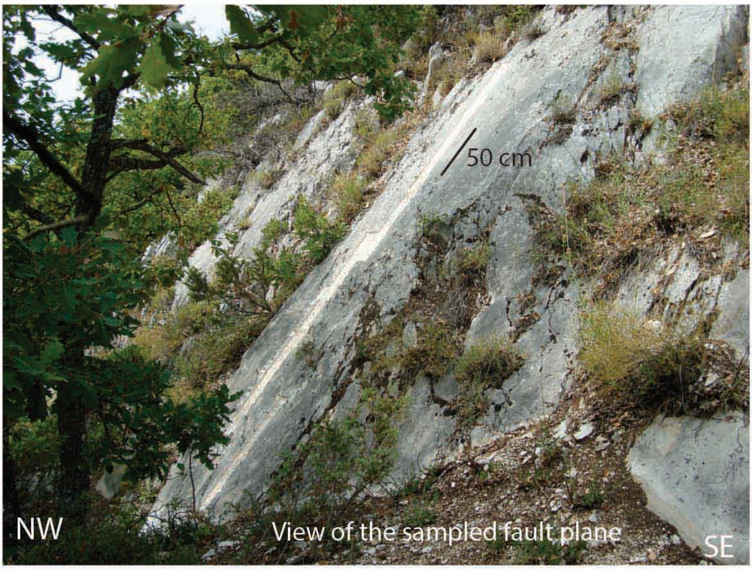
50 cm
NW
View of the sampled fault plane
SE

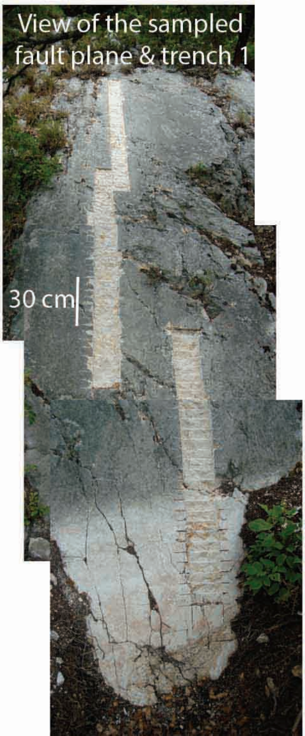
View of the sampled fault plane & trench 1
30 cm

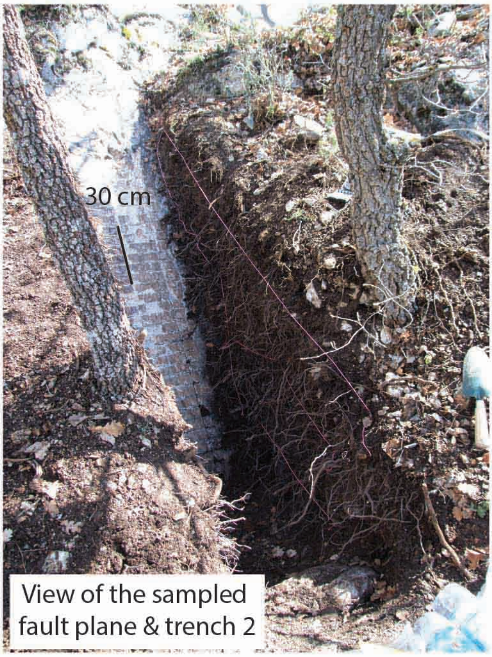
30 cm
View of the sampled fault plane & trench 2

Parasano

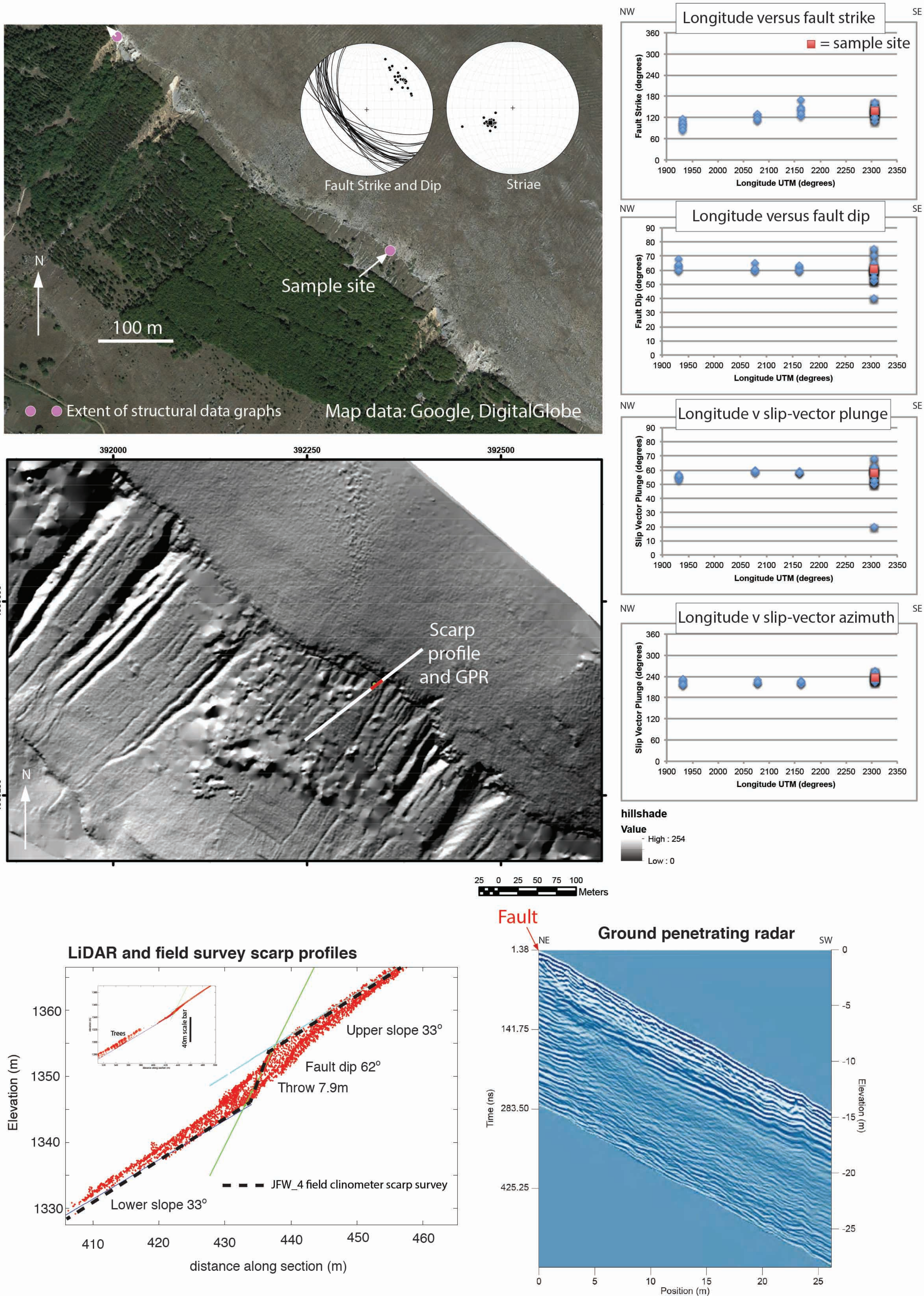

Fault Strike and Dip
Striae
N
Sample site
100 m
Extent of structural data graphs
Map data: Google, DigitalGlobe
NW
SE
Longitude versus fault strike
= sample site
Fault Strike (degrees)
Longitude UTM (degrees)
Longitude versus fault dip
Fault Dip (degrees)
Longitude v slip-vector plunge
Slip Vector Plunge (degrees)
Longitude v slip-vector azimuth
Slip Vector Plunge (degrees)
392000
392250
392500
4650500
4650250
Scarp profile and GPR
hillshade
Value
High : 254
Low : 0
25 0 25 50 75 100
Meters
LiDAR and field survey scarp profiles
Elevation (m)
distance along section (m)
Trees
40m scale bar
Upper slope 33°
Fault dip 62°
Throw 7.9m
Lower slope 33°
JFW_4 field clinometer scarp survey
Fault
Ground penetrating radar
NE
SW
Time (ns)
Elevation (m)
Position (m)

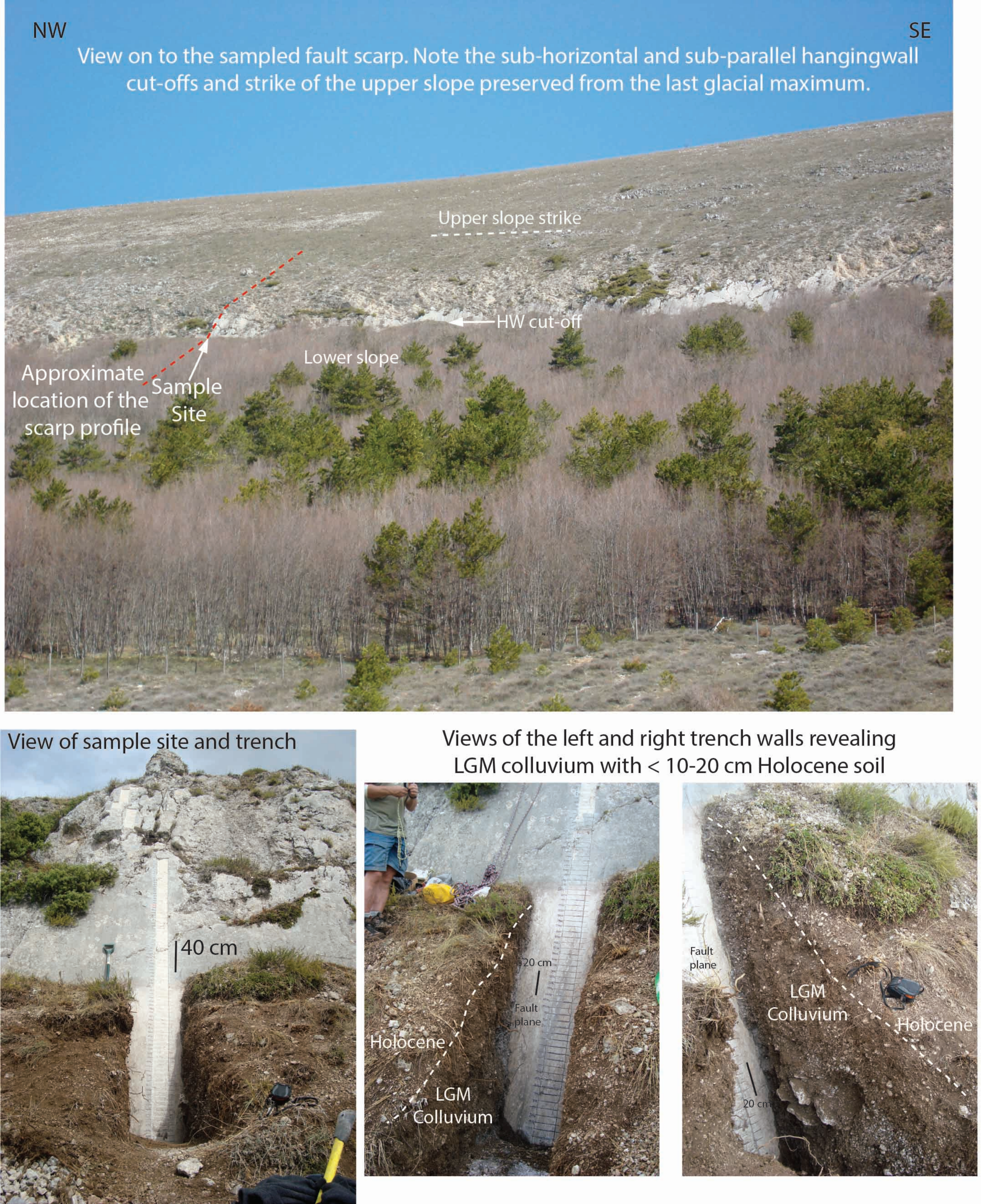
NW
SE
View on to the sampled fault scarp. Note the sub-horizontal and sub-parallel hangingwall cut-offs and strike of the upper slope preserved from the last glacial maximum.
Upper slope strike
HW cut-off
Lower slope
Approximate location of the scarp profile
Sample Site
View of sample site and trench
40 cm
Views of the left and right trench walls revealing LGM colluvium with < 10-20 cm Holocene soil
20 cm
Fault plane
Holocene
LGM Colluvium
Fault plane
LGM Colluvium
Holocene
20 cm

Pescasseroli

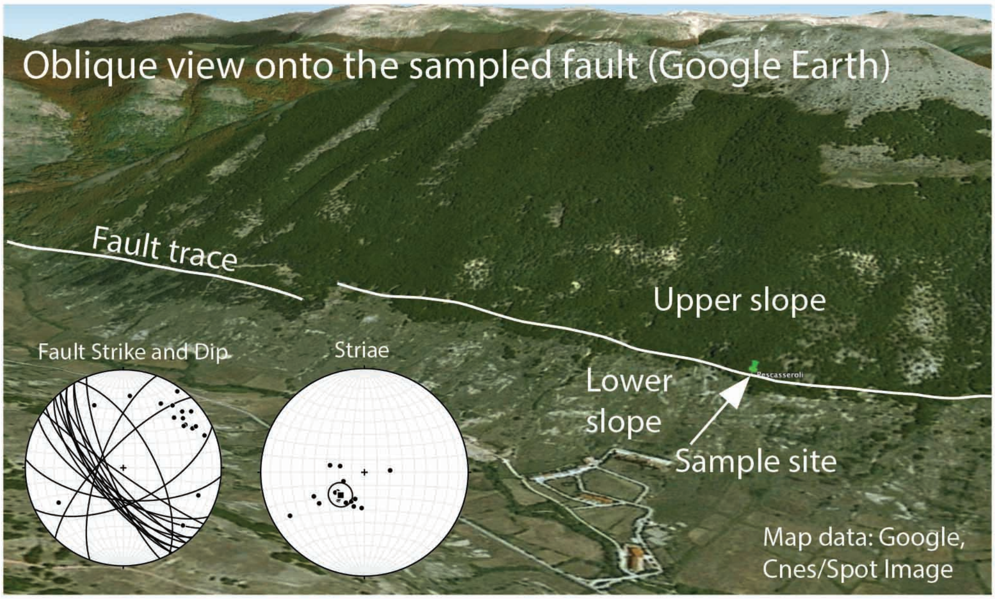

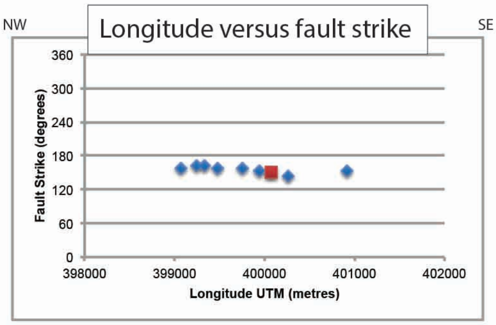

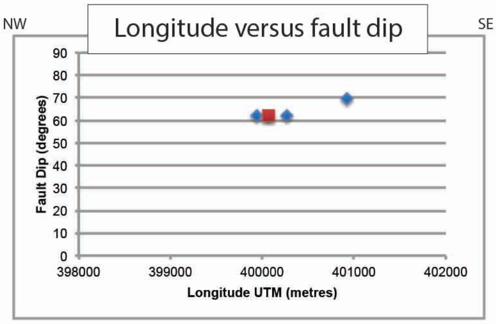

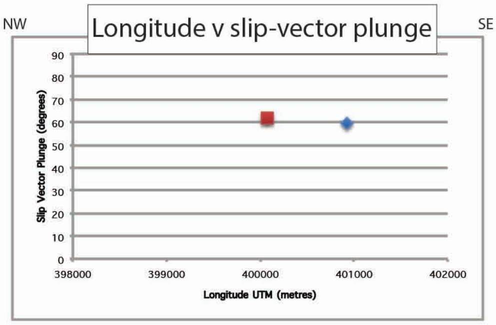

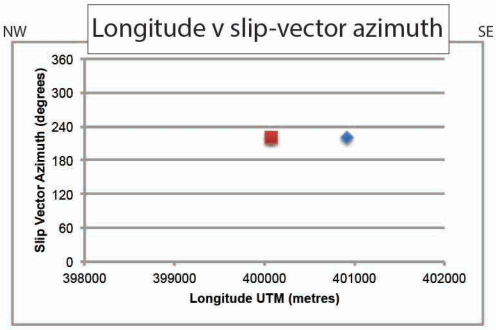

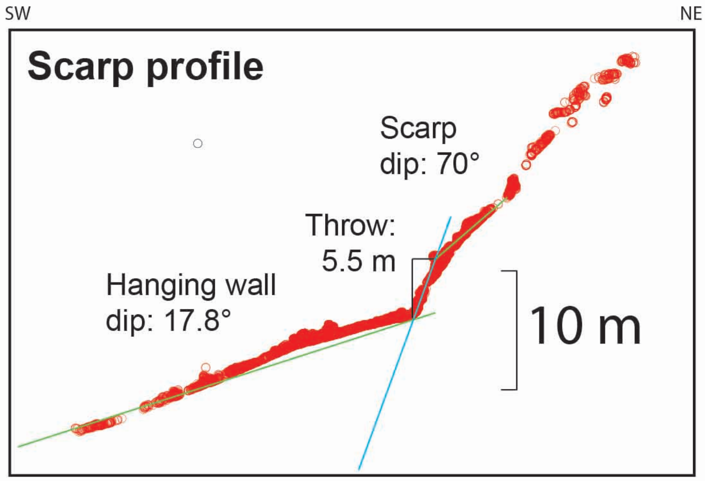

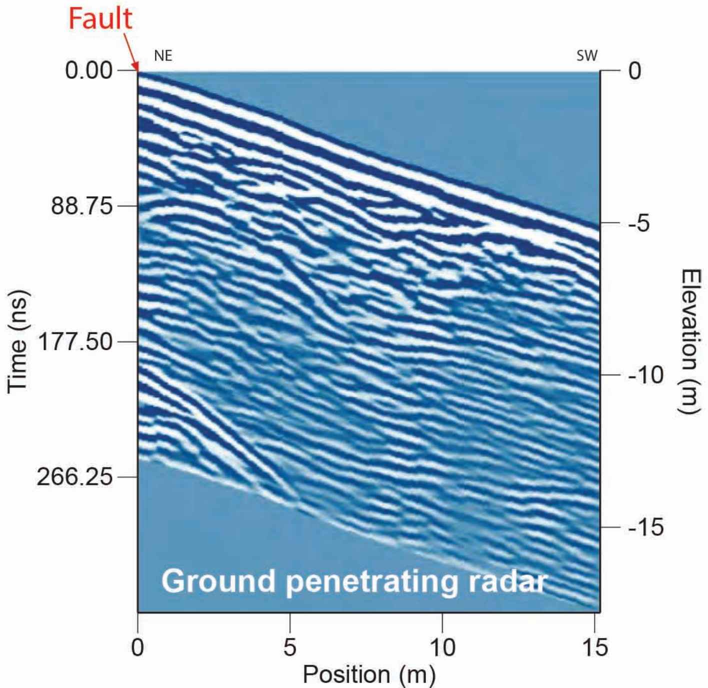

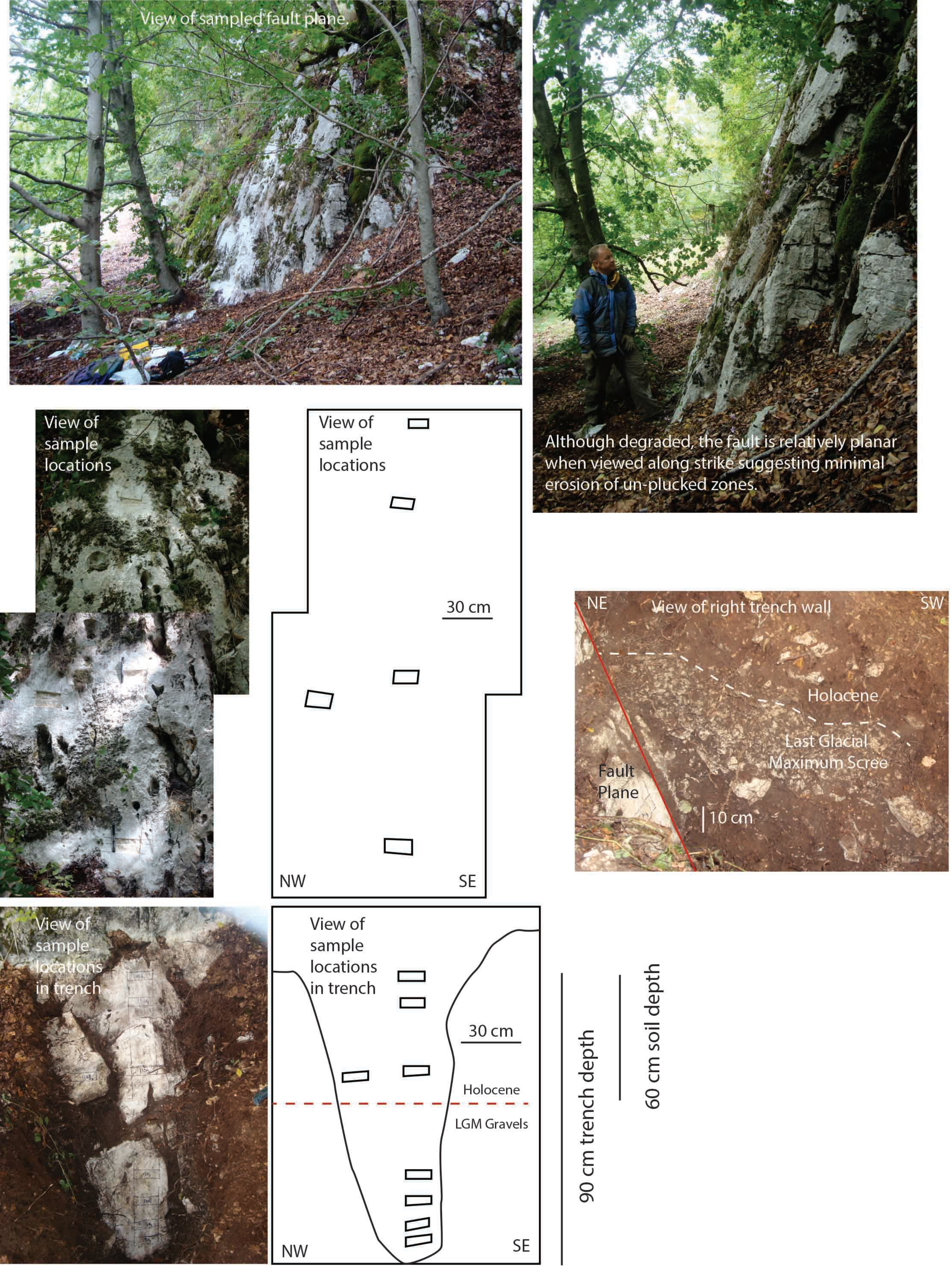
View of sampled fault plane.
Although degraded, the fault is relatively planar when viewed along strike suggesting minimal erosion of un-plucked zones.
View of sample locations
View of sample locations
30 cm
NW
SE
NE
View of right trench wall
SW
Holocene
Last Glacial Maximum Scree
Fault Plane
10 cm
View of sample locations in trench
View of sample locations in trench
30 cm
Holocene
LGM Gravels
NW
SE
90 cm trench depth
60 cm soil depth

San Sebastiano

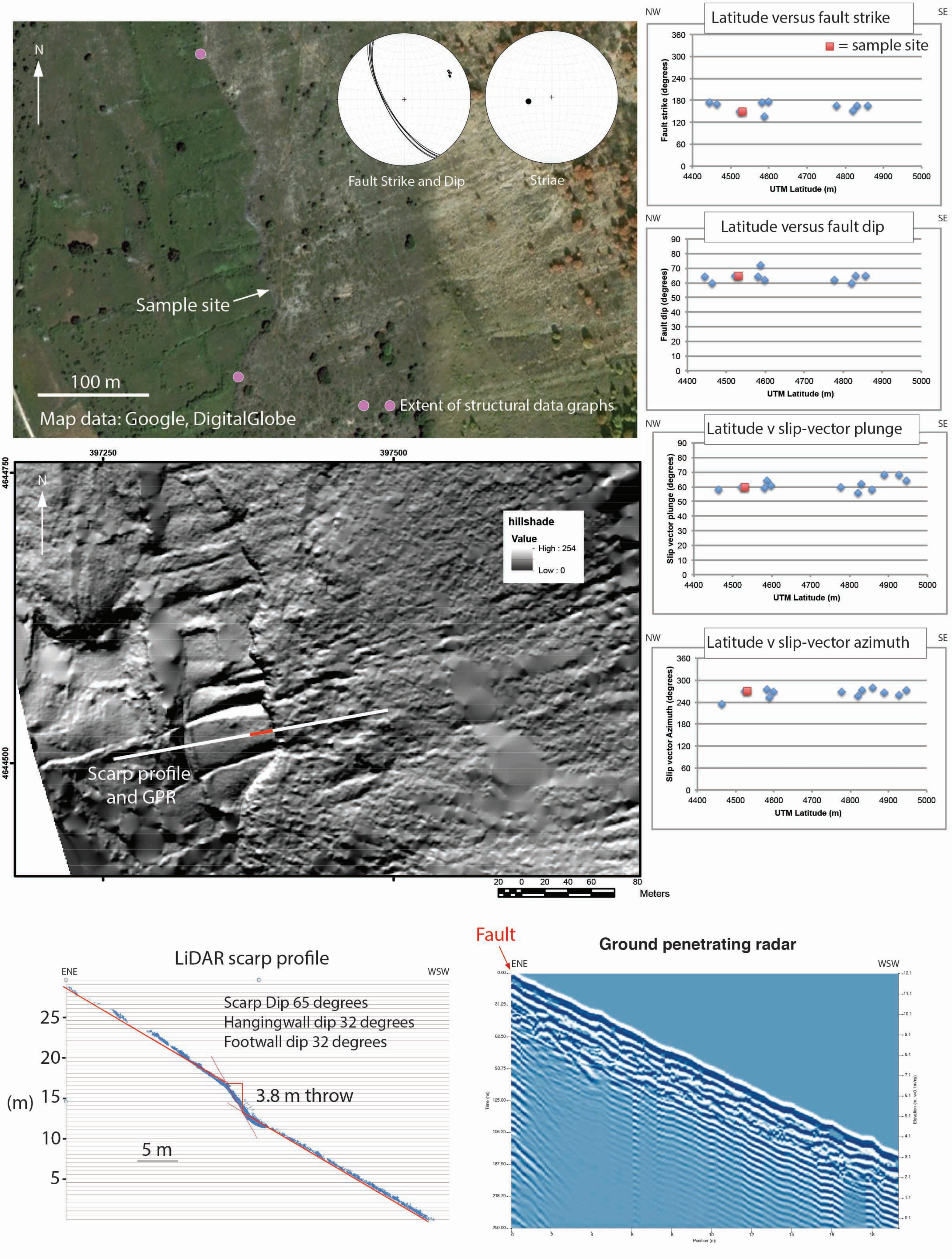

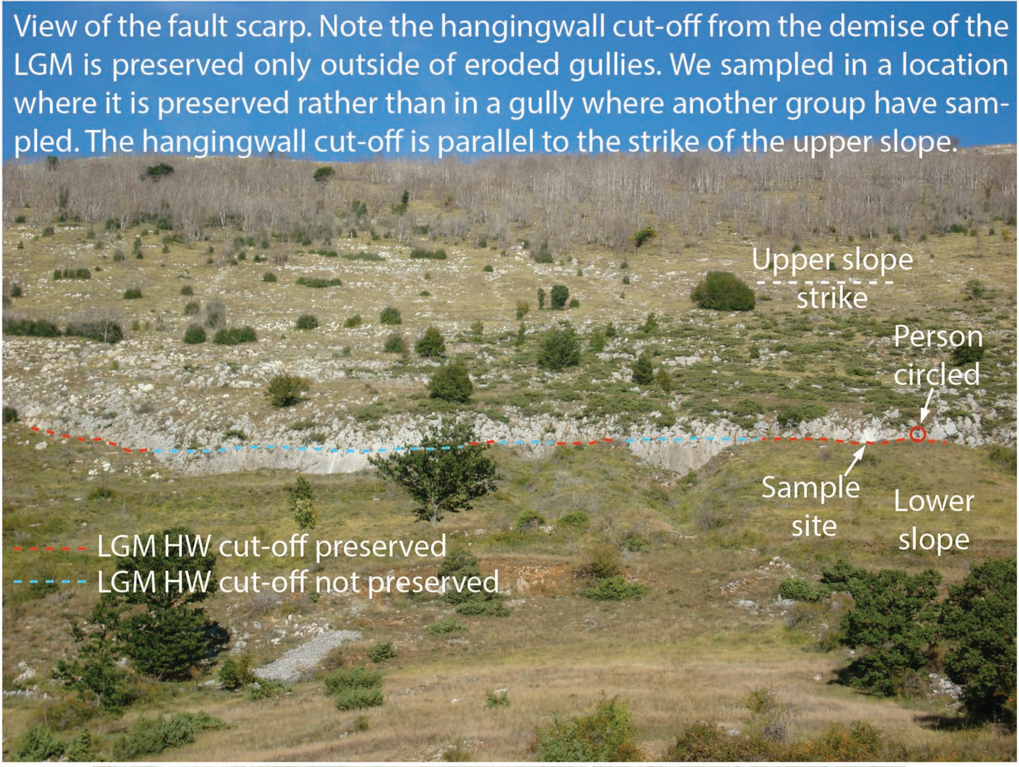
View of the fault scarp. Note the hangingwall cut-off from the demise of the LGM is preserved only outside of eroded gullies. We sampled in a location where it is preserved rather than in a gully where another group have sampled. The hangingwall cut-off is parallel to the strike of the upper slope.
Upper slope strike
Person circled
Sample site
Lower slope
LGM HW cut-off preserved
LGM HW cut-off not preserved

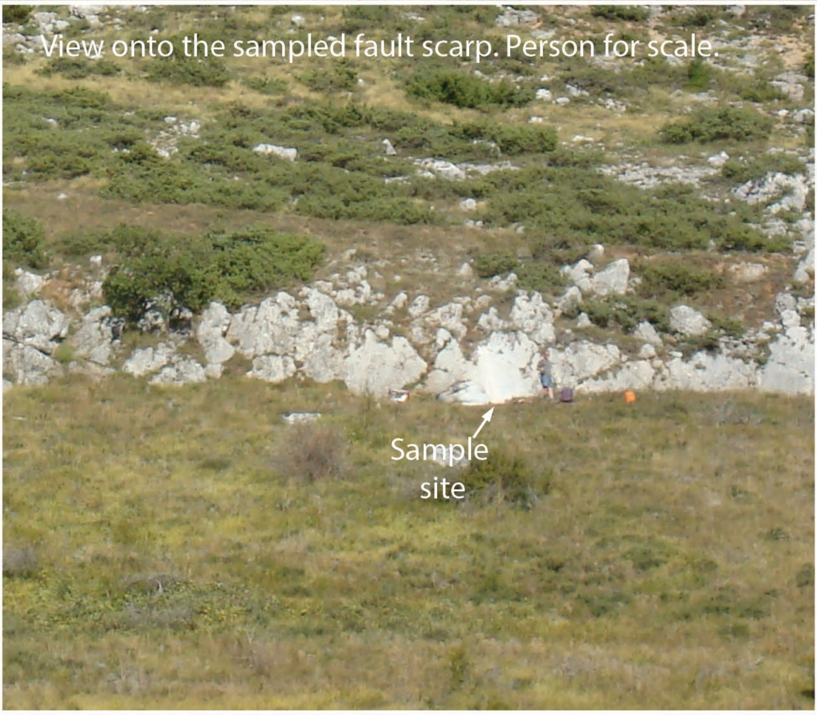
View onto the sampled fault scarp. Person for scale.
Sample site

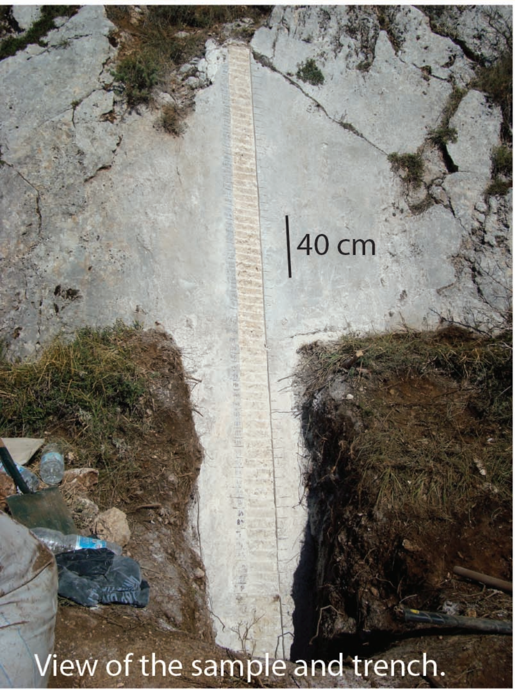
40 cm
View of the sample and trench.

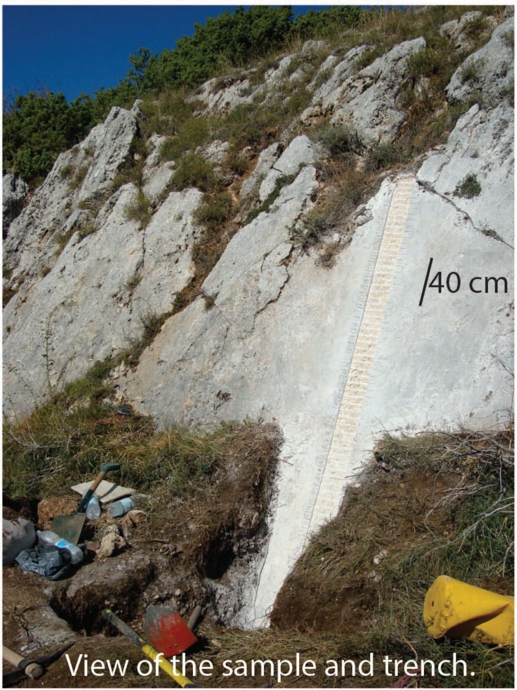
40 cm
View of the sample and trench.

Tre Monti

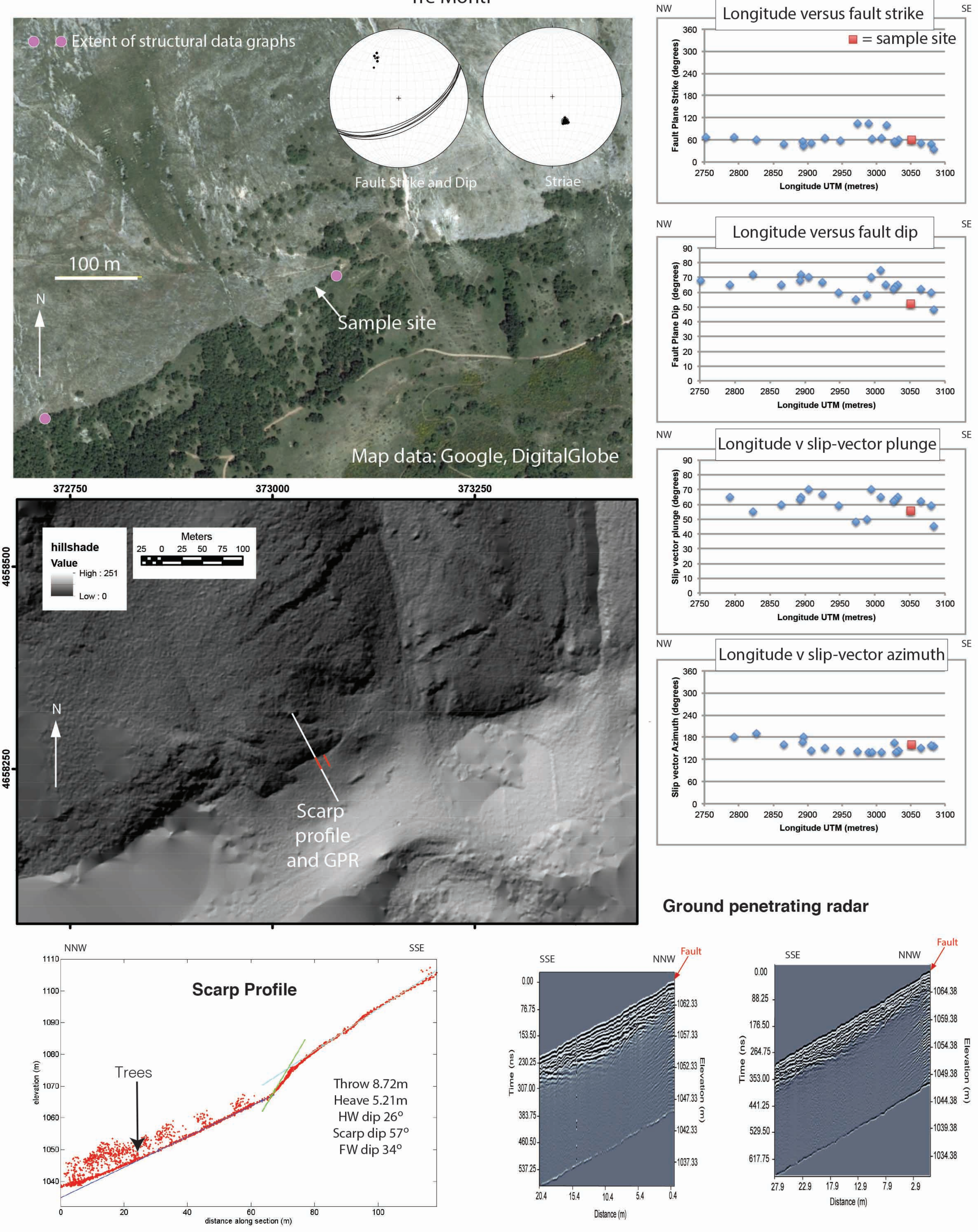

Extent of structural data graphs
Fault Strike and Dip
Striae
100 m
N
Sample site
Map data: Google, DigitalGlobe
372750
373000
373250
4658500
4658250
hillshade
Value
High : 251
Low : 0
Meters
25 0 25 50 75 100
N
Scarp profile and GPR
NW
SE
Longitude versus fault strike
= sample site
Fault Plane Strike (degrees)
Longitude UTM (metres)
Longitude versus fault dip
Fault Plane Dip (degrees)
Longitude v slip-vector plunge
Slip vector plunge (degrees)
Longitude v slip-vector azimuth
Slip vector Azimuth (degrees)
NNW
SSE
Scarp Profile
Trees
Throw 8.72m
Heave 5.21m
HW dip 26°
Scarp dip 57°
FW dip 34°
elevation (m)
distance along section (m)
Ground penetrating radar
SSE
NNW
Fault
Time (ns)
Elevation (m)
Distance (m)

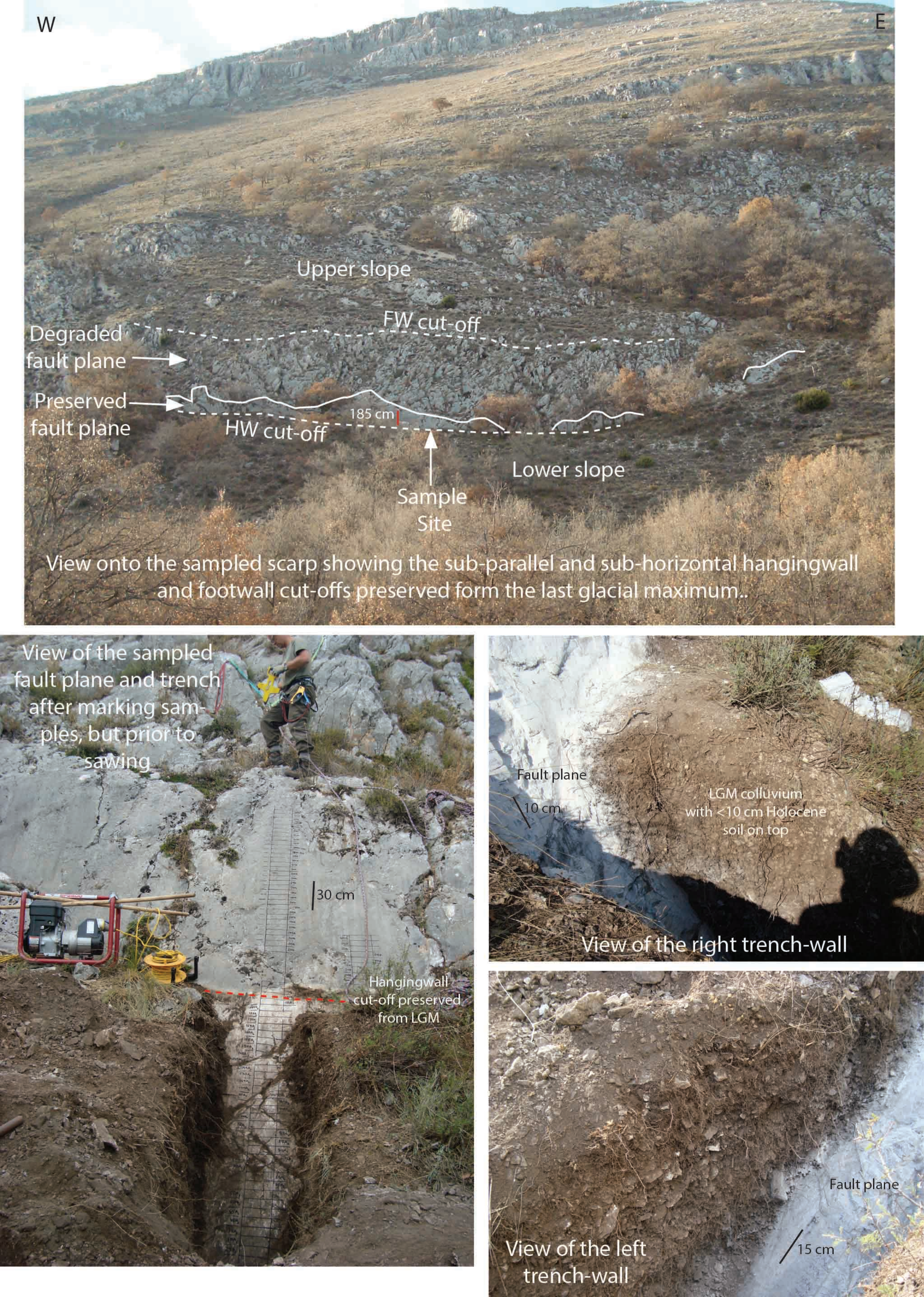
W
E
Upper slope
FW cut-off
Degraded fault plane
Preserved fault plane
185 cm
HW cut-off
Lower slope
Sample Site
View onto the sampled scarp showing the sub-parallel and sub-horizontal hangingwall and footwall cut-offs preserved form the last glacial maximum..
View of the sampled fault plane and trench after marking sam-ples, but prior to sawing
30 cm
Hangingwall cut-off preserved from LGM
Fault plane
10 cm
LGM colluvium with <10 cm Holocene soil on top
View of the right trench-wall
Fault plane
15 cm
View of the left trench-wall

4. Modelling

4.1 Methodology used for modelling fault plane samples

When fitting slip histories to the observed ^{36}Cl data for each fault site we consider three different approaches depending on the complexity apparent in the data and the presence of independent data from LiDAR, historical earthquake and paleoseismic records:
(i) Optimization;
(ii) Bayesian Markov Change Monte Carlo (MCMC) with fixed slip-rate change points;
(iii) Bayesian MCMC with flexible slip-rate change points that can be iterated.
In each approach we use a Matlab script that generates possible slip-histories and which calls another Matlab program, published by Schlagenhauf et al. (2010) for modelling ^{36}Cl concentrations on bedrock scarps, to quantify the fit of each of the slip histories we consider. Table S 4.4.1 contains all site characterisation data; Bayesian parameters and model results see Tables S 4.4.3 & S 4.4.4. N.B. In all model runs the site characterisation is fixed; we vary only the slip history parameters. We show the sensitivity to uncertainties in the site characterisation data below (see also Fig. S 4.2.1).

Measures of fit to the data

Schlagenhauf et al. (2010) suggest several measures of fit, including weighted Root Mean Squared Error weighted by the uncertainty of the measurements (RMSw), Akaike Information Criterion (AICc) and Chi-Squared. Here we use RMSw and AICc. RMSw is defined as:

$$RMSw = \sqrt{\sum_{i=1}^{n}\left[\left(\frac{O_i - M_i}{S_i}\right)^2\right]/n}$$

where $O_i$and M_i are the observed and modelled ^{36}Cl concentrations, S_i is the significance or error of the measurement and n is the number of measurements. Aikake's Information Criterion is defined as

$$AICc = n\,log\left[\sum_{i=1}^{n}((O_i - M_i)^2)/n\right] + \frac{2kn}{n-k-1}$$

where n is the number of observations and k is the number of parameters used in the model. In the (Schlagenhauf et al., 2010) approach k is taken to equal the number slip events used to model the data. This version of AICc is a modification of the original definition of AIC and should be used when the ratio $n/_k$ is small (i.e. ≤40), which it is in this study.

For the Bayesian parameter estimation approach we need to define the likelihood $P(O_i|\theta)$, that is the probability of observing the data (O_i) given the parameters (θ). A standard approach is to define the likelihood as:

$$P(O_i|\theta) = \frac{1}{\sqrt{2\pi\sigma^2}}exp\left(-\left(\frac{O_i - M_i}{2\sigma}\right)^2\right)$$

where σ is the standard deviation of the data. Note that this no longer includes the analytical error of the measurements that was included in the $RMSw$.

(i) Optimization approach (see sites PARA and SSB; Figs. S 4.5.6& S 4.5.7)

Optimization can be used in cases where the slip-history is simple and can be explained by few parameters. It involves systematic scanning of the parameter space and in practice works for the estimation of at most 2 parameters. This works well in cases where we assume the slip rate is constant as then we run the model for a number of scarp-ages within a possible range to find the scarp age (SA) that results in the minimum value of the Root Mean Squared weighted by the uncertainty of the measurements (RMSw) or the Akaike Information Criterion (AICc). The latter is strongly related to the RMSw but takes into account the number ofslip events used to model the data. Fig. S 4.1.1 shows an example for site PARA (full modelling results for this site in Fig. S 4.5.6).

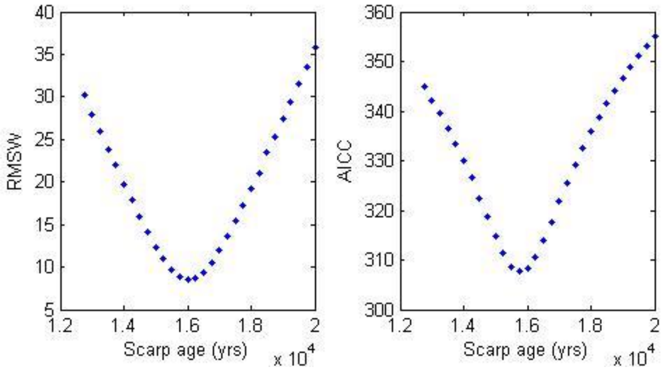

Fig. S 4.1.1 Optimisation approach assuming a constant slip rate (site PARA)

In the current paper slip-histories are modelled as a series of small slip events with constant offsets and inter-event times (slip size estimated from Wells and Coppersmith, 1994; Table S 4.4.1). We also considered offsets (or inter-event times) drawn from a random distribution with a given mean and standard deviation; tests showed that this did not change our conclusions and is not used in the results presented here.

In the case where the slip rate is not constant we need to infer the ages at which changes in the slip rate occurs as well as the total scarp age (SA). Initially, we assume that the height of the change point is known, based on independent observations of fault scarp morphology (e.g. from LIDAR measurements of surface roughness), see section (ii) below. In this case we generate a set of slips as before and count the number of offsets required to generate the sections of the scarp above and below the roughness change point(s). We assume constant slip rate above and below the change point(s) so that the inter-event times are given by the age of the change point and the number of slip events required.

(ii) Bayesian MCMC with fixed change-point heights

In the Bayesian approach we implement a sampling scheme based on the Metropolis Hastings algorithm (Metropolis et al. 1953, Hastings 1970, Sambridge et al. 2006). For a rigorous description of the underlying theory as well as a practical description of the implementation we refer to Sambridge et al. (2006). In brief, the method works as follows: The sampling starts with an arbitrary parameter set for which the fit to the data is calculated using the likelihood function. For each iteration a small random change is proposed to one of the parameters and the resulting fit is either accepted or rejected based on the ratio of the likelihoods. The method also allows for the inclusion of prior knowledge which in practice means that parameters that fit better with the prior distributions have a higher probability of being accepted (prior knowledge refers to information obtained from

independent sources, see more below). The theory behind the method prescribes that if we run this for long enough the sampled parameter sets will eventually “converge” and can be interpreted to represent the so called posterior distribution of the parameters, which takes into account the data as well as the prior knowledge about the parameters (Fig. S 4.1.2 is example below for site FIAM).

In our implementation for the ^{36}Cl data on fault scarps, the offset for each slip event is considered constant. The slip history in the fixed change point approach is fully defined by the elapsed time, ET (= time elapsed since most recent accrued slip), the height and age of the total scarp and the heights and ages of any number of change-points, CP1 etc. In practice we limit the number to those for which we have independent evidence (at most 2 change points) using measures of fault surface roughness from terrestrial LiDAR data. In each iteration we make a small change to the elapsed time, the scarp age or the age of any of the change points. An illustration of the results from this approach for site FIAM is given in Figs. S 4.1.2&S 4.1.3 where the estimated ages and likelihood are plotted versus iteration number. In that particular case we fix the elapsed at 665 yrs (before present), based on the timing of the 1349 AD earthquake on this fault (Guerrieri et al., 2002) and include change-points at 14.00 and 22.40 m height on the scarp (Fig. S 4.5.1 & S 4.5.2). The prior distribution for the scarp age (SA) is a normal distribution with mean of 15000 yrs and standard deviation 2500 yrs (implying that we are 95% certain that the scarp age is between 10000 and 20000 yrs). This is based on the timing of the demise of the LGM in this area (Giraudi & Frezzoti, 1997), associated with a marked drop in hill slope erosion rates and the onset of scarp preservation (Tucker et al., 2011), and also the upper slope age that we obtained at this site (see Section 4.1.1). Full modelling results for FIAM are shown in Figs. S 4.5.1, S 4.5.2 and S 4.5.3.

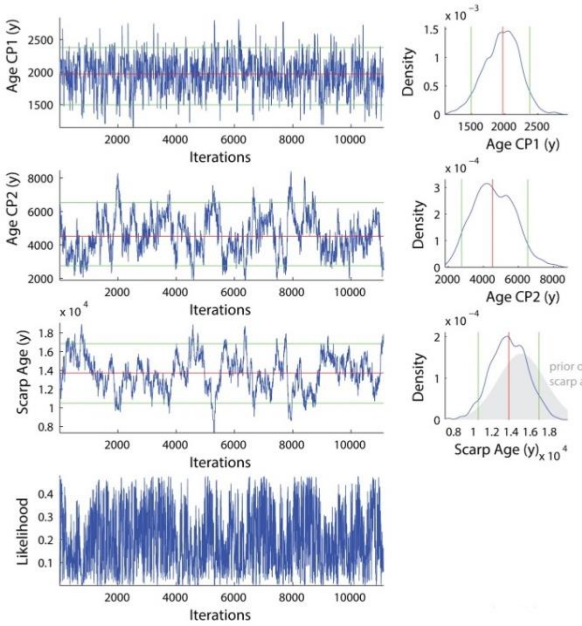

Fig. S 4.1.2 Trace plots for site FIAM showing age estimates as a function of model iteration for CP1, CP2 and SA (Scarp Age) and the resulting probability density functions (red line = median posterior and green lines mark 90% credible interval (CI)). Trace fluctuations show that parameter space is continuously explored. Convergence is indicated by trace returning to a similar value after a deviation.

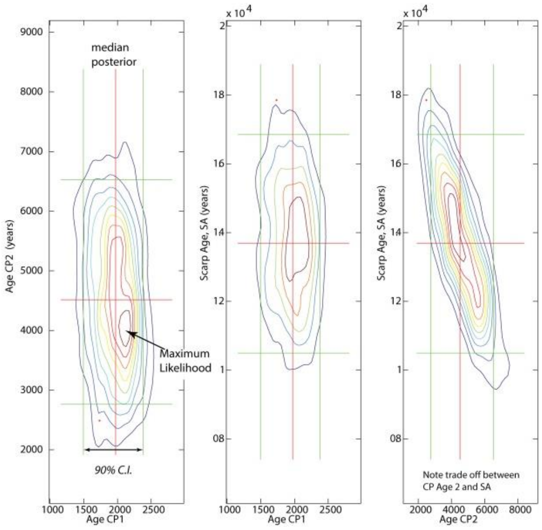

Fig. 4.1.3 Contours of likelihood for CP1, CP2 and SA age estimates derived from trace plot data for site FIAM (see Fig. S 4.1.2). Red line = median posterior and green lines mark 90% credible interval (CI). CP1 has the most tightly constrained age. CP2 and SA are negatively correlated with each other. Full results for this site shown in Figs. S 4.5.1 & S 4.5.2 & S 4.5.3.

(iii)   Bayesian MCMC with flexible change-point heights that can be iterated

The Bayesian MCMC approach with fixed change points works well when we have some independent knowledge about the position along the fault where a change occurs. If we do not have such information we have to infer the presence and height of change points as well as their timings. This can be achieved using Bayesian Reversible Jump MCMC (Green 1995, Sambridge et al. 2006). In this approach, the number and heights of change-points is not fixed. In each iteration we either add, or remove change-points and/or change the timing of existing ones in addition to changing the elapsed time (ET) and/or scarp age (SA). As before, the slip rate between change points is assumed to be constant and as define in method (ii) above. Although any number of change-points can in theory be considered, the method favors simple solutions with few change-points (Sambridge et al. 2006) and in our results the number of change-points never exceeded 6. Due to the fact that this is a very high dimensional parameter space in which each offset is a potential change point this method takes a long time to converge (cf. trace plots for fixed change points Fig. S 4.1.2). However, we can consider the likelihood of all the slip-histories explored and use this to represent the best fitting slip-histories (Fig. S 4.1.4). In this flexible change point approach we only present posterior distributions and quote credible intervals for estimates of ET and SA for which we do obtain convergence.

For the site FRAT we applied both the fixed and flexible change point methods. Fig. S 4.1.4 shows that both methods lead to similar results (full modelling results for FRAT site: Fig. S 4.5.9). The fixed change point CP1 at FRAT was selected based on the height of the best preserved (smoothest) part of the fault plane. This height corresponded to the height of our sample ladder and based on the detailed roughness analysis at site FIAM (Figure S 4.5.1 and S 4.5.2) we know that a lower change point can be reliably defined in this way. For simplicity we set CP2 = CP1 + 1 cm in the fixed change point method; the flexible change point result suggests that the change may be slightly more gradual.

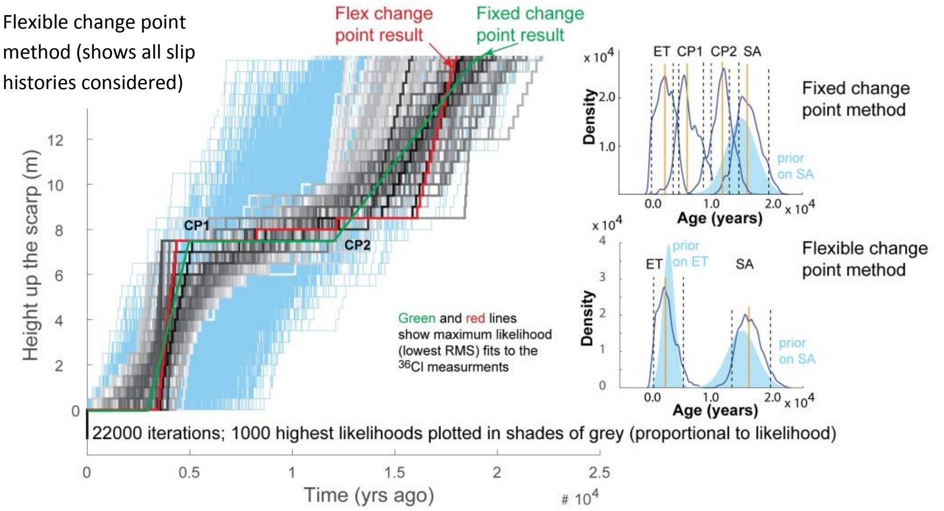

Fig. S 4.1.4 Comparison between fixed and flexible change point modelling approaches applied to site FRAT. Both fit the data with a similar, low RMS: 6.8 and 7.6 respectively.

Sensitivity tests

Figures S 4.2.1, S 4.2.2 and S 4.2.3 presents sensitivity tests regarding site characterization data, temporal slip rate variability (SRV) and the elapsed time (ET). We also applied the flexible change method to synthetic data (Fig. S 4.3) to demonstrate the robustness of our Bayesian approach. Our SRV sensitivity tests (Fig. S 4.2.2) use theoretically generated slip histories from the model of Cowie et al. (2012) in which (i) the viscous part of the crust is ignored, (ii) 100% coseismic stress drop is

transferred onto neighbouring structures, and (iii) fault healing is instantaneous. Consequently, large values of SRV reflect the dominant elastic interaction effect whereas in natural systems we know that the viscous lower crust has finite strength (ref. 9 main text), the magnitude of coseismic stress transfer is ~1% of earthquake stress drops (ref. 27 main text), and fault healing is not instantaneous. We use the slip histories from Cowie et al (2012) only as a source of synthetic data sets for testing our Bayesian approach.

Firstly, the impact of uncertainties that arise from site geometry characterised by LiDAR and structural data gathered in the field (Table S 4.4.1) are compared to uncertainty in scarp age (SA) that we anticipate based on the timing of the demise of the LGM in this area (12 – 18 ka). Figure S 4.2.1 shows that the range in SA dominates all of the other uncertainties related to site geometry and this is why we include it as prior information in the Bayesian modelling. Moreover, the range in SA also means that we can only resolve with confidence temporal variations in slip rate above a certain magnitude (Fig. S 4.2.2): Slip Rate Variability (SRV) characterized by SRV < 0.2 is too subtle to resolve with confidence in this study whereas SRV values > 0.3 become better resolved as SRV increases (Fig. S 4.2.2). The implication of the sensitivity tests shown in Figure S 4.2.2 is that where we have inferred SRV = 0.0 for the field data we cannot exclude some temporal variations in slip rate but where we estimate SRV > 0.3 we are confident that the rate variations have indeed been significant (i.e., periods of rapid slip interspersed with periods of relative quiescence). Table S 4.4.2 summarises our SRV analysis results.

In addition to slip rate variations, a long elapsed time (several hundreds to thousands of years) can be resolved by a change in slope in the ^{36}Cl profile at the ground surface (Fig. S 4.2.3). The trench portion of each sample ladder is generally pristine as it is not yet exhumed and the lowermost portion of the subaerial scarp has had the least amount of time to be modified by any surface processes. These factors permit denser sampling. The ^{36}Cl concentration at the top of the trench as well as the increase in ^{36}Cl concentration versus height along the fault plane both in the trench itself and in the lowermost part of the subaerial scarp are particularly sensitive measures of both SRV and long ET (see theoretical prediction in Fig. S 4.2.3(b&c)). Our field data are compared to the theory in Figure S 4.2.3(d) and confirm our overall interpretation that the 'fanning' pattern shown in Figure 3 in the main part of the paper reflects variations in average slip rate and that deviations from the simple fan shape reflect temporal variations in slip rate (SRV) and/or long elapsed times (ETs).

We also use synthetic data to test the flexible change point Bayesian approach (Fig. S 4.3). Using a slip history characterized by SRV $\approx$ 0.4 and Scarp Age (SA) = 15.6 ka (Fig. S 4.2.2(c)) we generated a synthetic ^{36}Cl data set with the site geometry, sample spacing and sample chemistry at site PARA. These data were then treated in exactly the same way as our field data to demonstrate not only the success of our approach in recovering the main features of the slip history, but also to determine the window length that best characterizes the SRV both for the actual slip history and the slip history inferred from the ^{36}Cl profile (Fig. S 4.3c&d). We obtain the median posterior estimate of SA = 14.6 ka (+4.0/-3.2 ka at the 90% C.I., which overlaps the actual SA = 15.6 ka); the maximum likelihood estimate SA = 14.4 ka. The maximum likelihood slip history has an SRV = 0.45, compared to 0.44 for the actual slip history, both calculated using a 3000 year sliding window. Furthermore, the SRV for the five highest likelihood fits range from 0.2 to 0.6, i.e, all resolve that SRV > 0 for the synthetic data.

4.1.1 Methodology used for modelling upper slope sample at site FIAM (LGM inheritance)

Using the exposure age calculator of Schimmelfennig (2009) we obtain an analytical age for the upper slope sample at site FIAM of 19.25 $\pm$ 1.81 ka. This age relates to the timing of stabilisation of the upper slope and the onset of bedrock scarp preservation associated with an order of magnitude drop in hill slope erosion rates at the end of the Last Glacial Maximum (LGM)/beginning of the Holocene (Tucker et al., 2011). Using Eqn. 3 in Tucker et al. (2011) we estimate the LGM erosion rate as 0.28 $\pm$ 0.05 mm/yr at this site (calculated from the site geometry). This erosion rate is similar to the rates (0.2 – 0.4 mm/yr) estimated by Tucker et al. for this area during the LGM and similar to rates calculated for all of our other sites. These erosion rates were sufficient to remove >85% of any ^{36}Cl that accumulated hence why we interpret the measured upper slope ^{36}Cl concentration as indicative of slope stabilisation and use Schimmelfennig's (2009) age calculator for this sample. Correcting for the finite erosion rate estimated for this site, using Tucker et al.'s formula, we obtain a 'corrected' upper slope stabilisation age of 17.0 +1.7/-1.8 ka (here the uncertainty includes both analytical uncertainty and uncertainty in the erosion rate estimate). This 'corrected age' lies within the 12-18 ka age range for the demise of the LGM in this area (Giraudi and Frezzoti, 1997).

The prior distribution for Scarp Age (SA) used in our Bayesian modelling of the fault plane samples (see Section 4.1) implies that we are 95% certain that the SA is between 10000 and 20000 yrs. It is a normal distribution based on the information on the 12 – 18 ka age range for the demise of the LGM from Giraudi and Frezzoti (1997). Any effect of inherited ^{36}Cl on SA from finite LGM erosion rates (≈ +/-2 kyrs, see calculation above) thus lies well within the range of our prior and is less than the C.I.'s that we quote on results in this paper. In other words, the effect of inherited ^{36}Cl on our overall results/conclusions is negligible because we have taken a conservative approach in our implementation of the Bayesian MCMC methodology. We demonstrate this, for example, in Fig. S 4.3 where, for the synthetic data, we quote the 90% C.I. on our estimate of SA to be +4.0/-3.2 ka and our estimates of SRV for the five highest likelihood fits = 0.4 ± 0.2, obtained using a 3000 year sliding window (Fig. S 4.3d; the actual SRV is 0.44). In Fig. S 4.2.2 we also show why, in this paper, we do not interpret values of SRV < 0.2 as significantly different from SRV = 0.

4.1.2 Summary of ^{36}Cl production rates used in the Schlagenhauf et al. (2010) Matlab code

Spallation on Ca: $\Psi_{36Cl\_Ca0} = 48.8 \pm 3.5$ at. of ^{36}Cl. g of Ca . yr^{-1} (Stone *et al.* 1996)
Spallation on K: 162 ± 24 at. of ^{36}Cl. g of K . yr^{-1} (Evans *et al.* 1997)
Spallation on Ti: 13 ± 3 at. of ^{36}Cl. g of Ti . yr^{-1} (Fink *et al.* 2000)
Spallation on Fe: 1.9 ± 0.2 at. of ^{36}Cl. g of Ti . yr^{-1} (Stone 2005)
Slow negative muons stopping rate at land surface: $\Psi_{\mu,0} = 190$ muon.g^{-1} yr^{-1} (Heisinger *et al.* 2002)
Neutron attenuation length: 208 g cm^{-2} (e.g. Gosse and Phillips, 2001)
Neutron apparent attenuation length for a horizontal unshielded surface: 160 g cm^{-2}
Muon apparent attenuation length for a horizontal unshielded surface: 1500 g cm^{-2}

Sections 4.2-4.6 Figures and Tables only

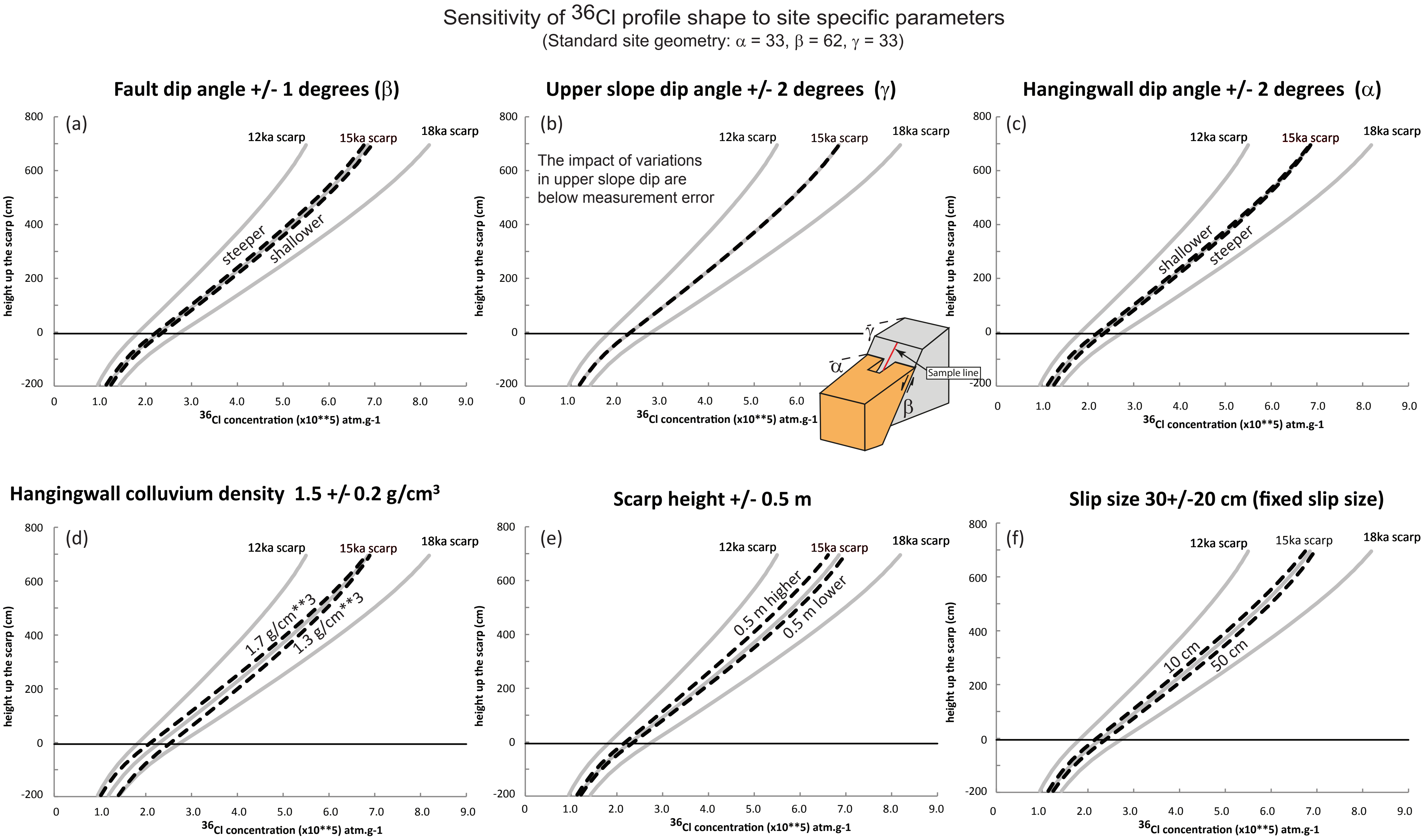

Figure S 4.2.1. Impact on ^{36}Cl profile shape of uncertainties that arise from site geometry characterised by LiDAR and structural data gathered in the field. All tests (black dashed lines) are based on a single typical field site (similar to PARA) with a 15 ka scarp age. In (b) the variations in scarp height derive from the combined measurement error on upper slope dip angle and fault plane dip. In (d) density range reflects the range of densities measured across all our sites. We use one value for each site but also test that our conclusions on SRV are not sensitive to the specific value. Effect of variations in slip rate (SRV) are considered in Figure S.4.2.2. Differences in scarp age (SA) for the standard site (see grey lines) reflect the age range that defines the demise of Last Glacial Maximum (LGM) = 12-18ka (Giraudi & Frezotti, 1997). The uncertainty in SA clearly dominates over other sources of uncertainty associated with the site geometry. We include prior information on SA in our Bayesian modelling approach (see Section 4.1 of Supplementary Material).

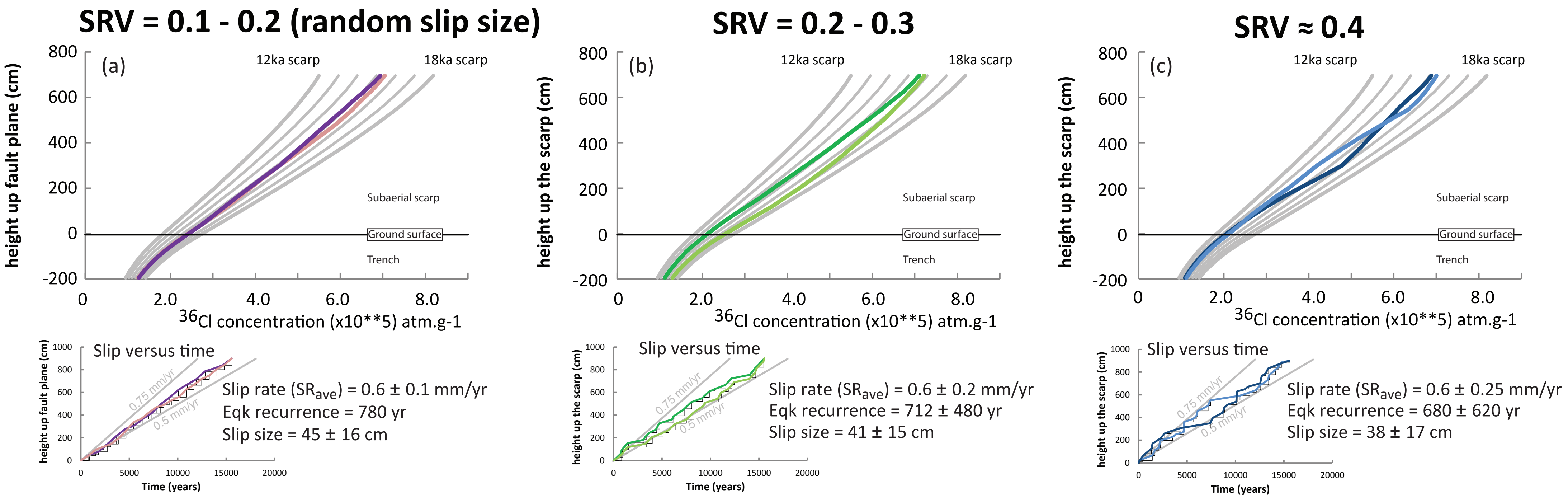

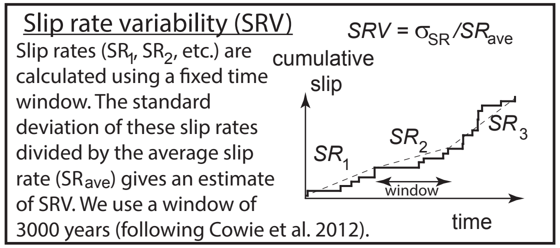

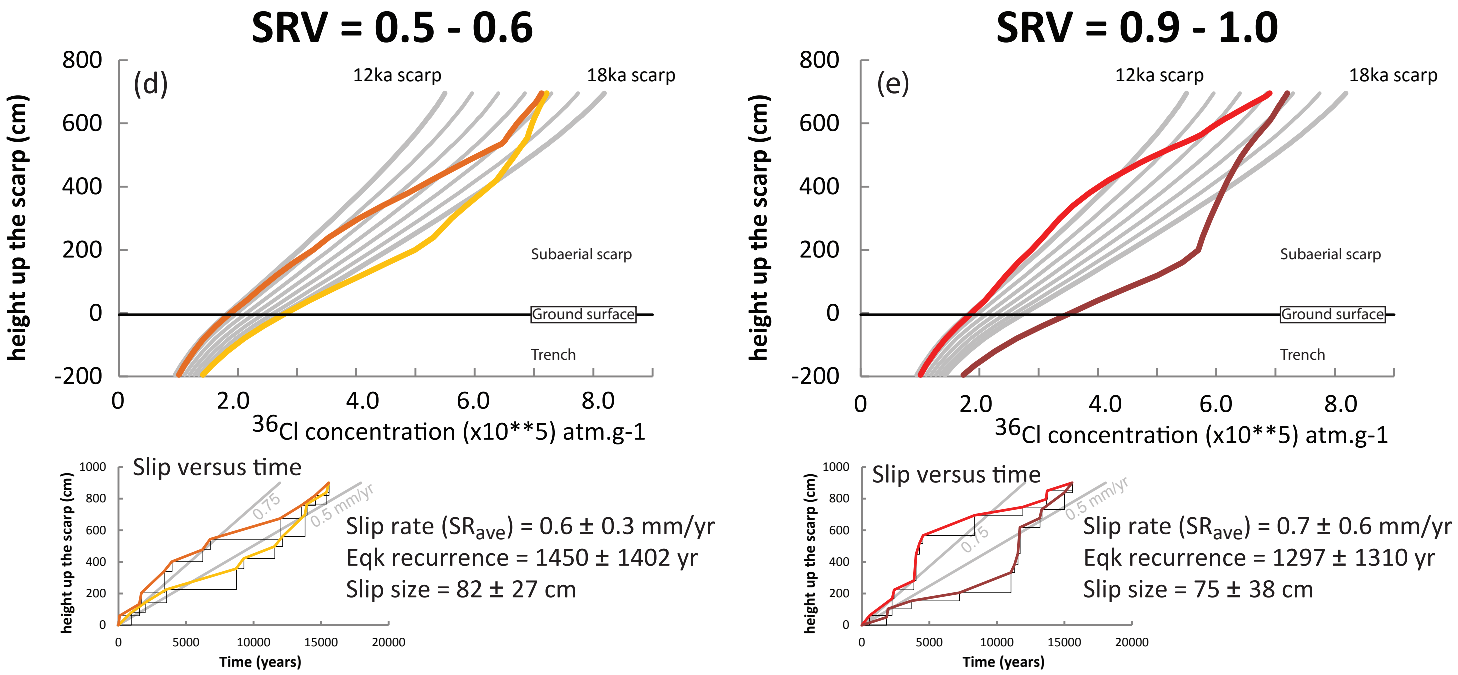

Figure S 4.2.2. Sensitivity of modelled ^{36}Cl profiles to temporal variations in slip rate using synthetic data from the study of fault interaction in Cowie et al. (2012). SRV was calculated for each synthetic data set using a 3000 year window length. The average slip rate (SR_{ave}) as well as the earthquake recurrence statistics are given for each case. The same standard site geometry (see Figure S.4.2.1) is used for all of the examples shown here. Constant rate slip histories are shown by grey lines and denote the range of rates associated with the 12 - 18 k yr time window that defines the demise of the LGM in this area (Giraudi and Frezzotti, 1997). Values of SRV < 0.4 become progressively more difficult to resolve from a constant slip rate (see examples a - c above).

Influence of SRV and ET on ^{36}Cl profile shape in the trench

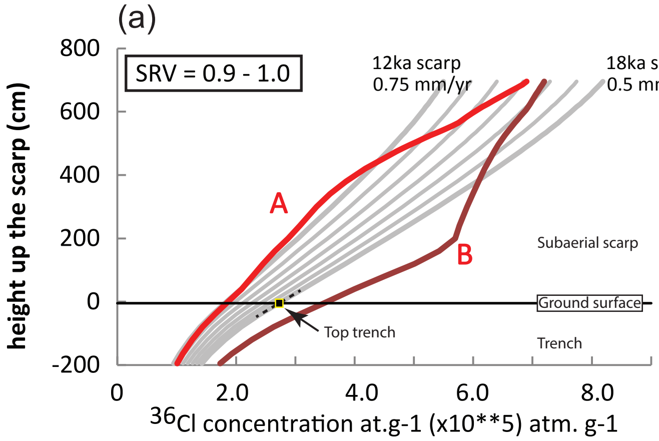

Figure S 4.2.3(a). Location of top trench sample (see black/yellow square). Dashed line indicates the rate of increase of ^{36}Cl concentration with height up the scarp at the trench top. The 36Cl profiles shown here are taken from the synthetic data used to analyse the effect of SRV (see Fig. S. 4.2.2(e)). Grey lines are for constant slip rates. The trench top ^{36}Cl concentration and the increase in ^{36}Cl per cm of the scarp is plotted in Fig. S 4.2.3(c) (a) for the cases A and B (for which SRV = 0.9 - 1.0).

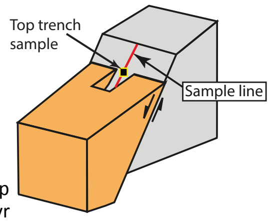

The trench portion of the sampled fault plane provides a strong constraint on the slip history. Not only is it by definition unaffected by surface processes, the decrease in 36Cl concentration with depth or any abrupt changes in ^{36}Cl at the top of the trench are sensitive measures of SRV (Fig. S 4.2.3(a & b) and time that elapsed (ET) since the fault last accumulated significant offset (see D in Fig. S 4.2.3(b)).

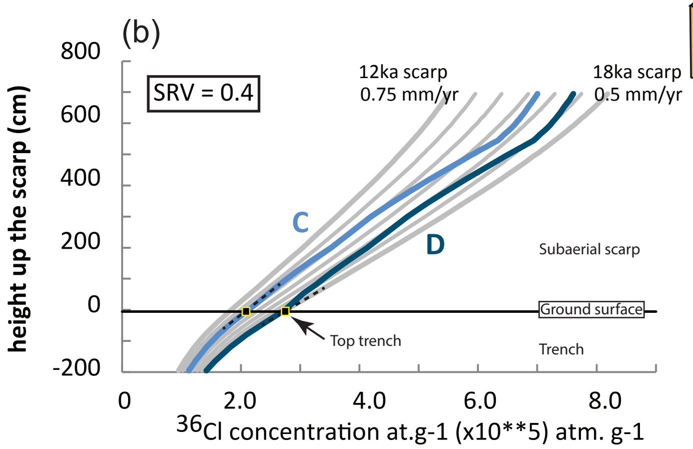

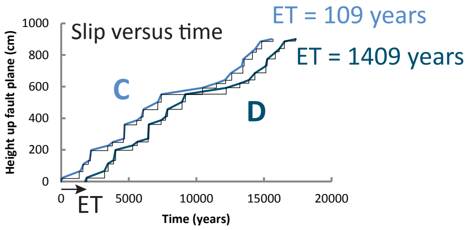

Figure S. 4.2.3(b). Effect of long elasped time (ET) on trench top ^{36}Cl concentration and ^{36}Cl profile shape. Note change in gradient of ^{36}Cl profile (case D) at the ground surface that is not apparent when ET is short, i.e., case C. Above the ground surface the increase is ^{36}Cl with height up the scarp is less than it is in the upper part of the trench in case D. Profile C is taken from synthetic data shown in Fig. S 4.2.2(c). Profile D is the same slip history but with an additional ET = mean earthquake recurrence interval plus 1 standard deviation. The trench top ^{36}Cl concentration and the increase in ^{36}Cl per cm of the scarp are plotted in Fig. S 4.2.3(c) marked by the letter D.

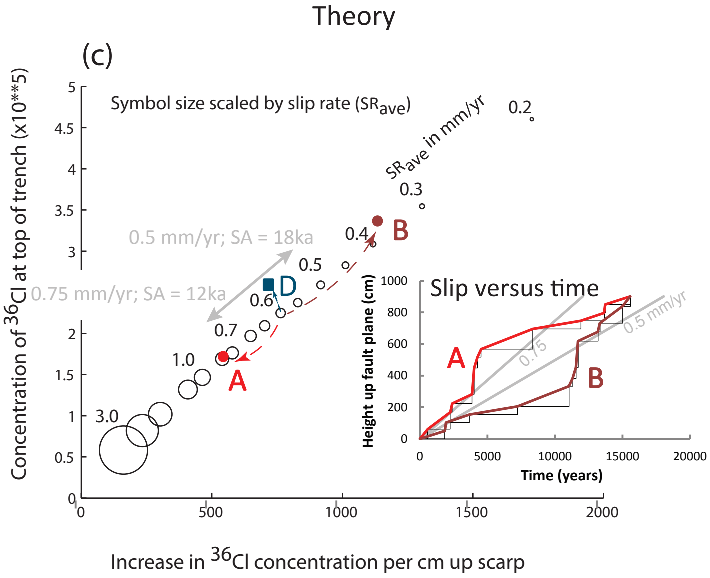

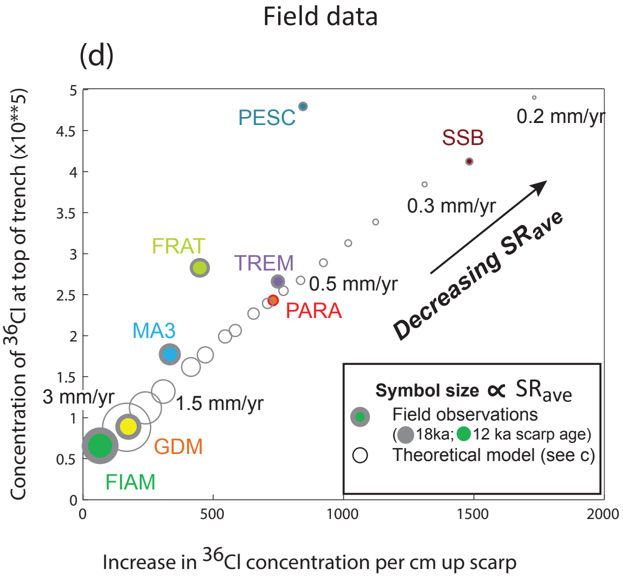

Figure S 4.2.3(c & d). Comparison between theory and field data for ^{36}Cl concentrations measured at the top of the trench. On faults that have a lower average slip rate (lower SR_{ave}) the ^{36}Cl concentration is higher and the rate of increase in ^{36}Cl concentration with height on the scarp is greater. The real data show a similar correlation but with more scatter, partly due to temporal variations in slip rate (SRV) and long elapsed times (see theoretical cases A, B and D in (c)) but also due to variations in cosmogenic production rate between sites. Grey arrow in (c) indicates the range within which SRV > 0 is difficult to resolve with only trench samples because of the likely range in total scarp age (i.e., 12 - 18 ka; Giraudi & Frezzotti, 1997)

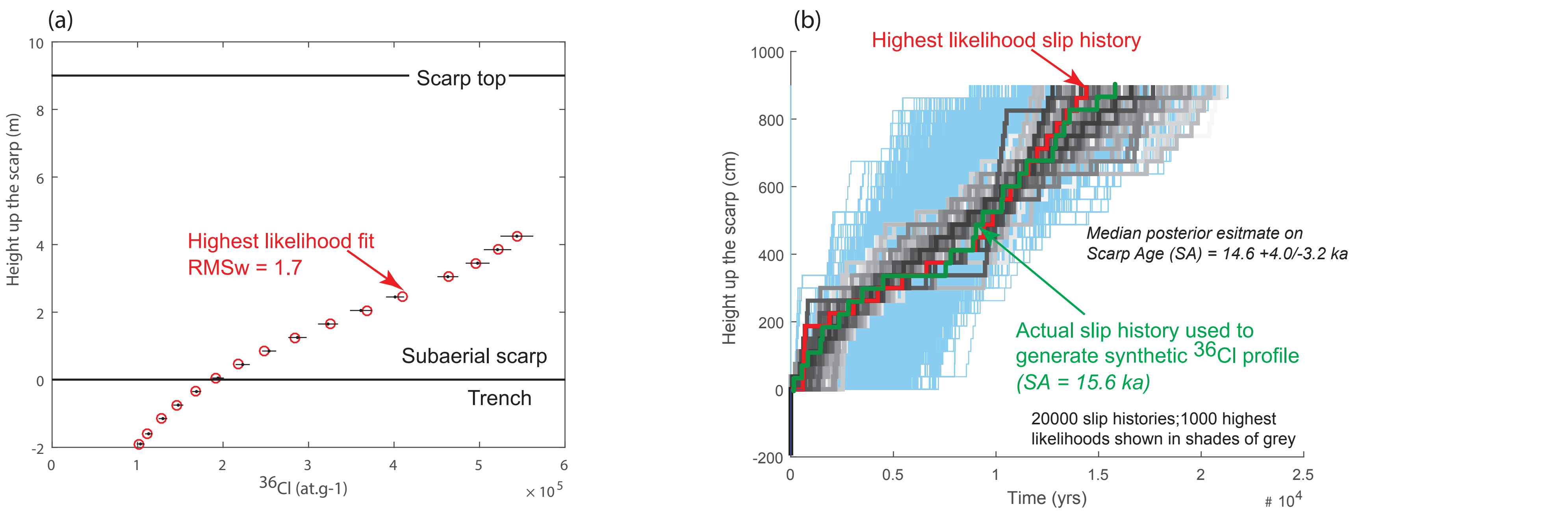

Figure S 4.3. Testing the flexible change point method using synthetic data. (a) Maximum likelihood fit (red circles) to a synthetic ^{36}Cl data set (black points) after 20000 iterations using the flexible change point method. The actual slip history used to generate the synthetic ^{36}Cl profile is shown by the green line in (b), overlain on the results of the modelling. The synthetic data set was generated assuming the same chemistry for each sample; the sample spacing and the analytical error bars are the same as that of a real site in this study (PARA). The modelling of the ^{36}Cl data used the known site geometry (α, β, γ), scarp height, site elevation, and slip size to solve for the timing of each slip event and thus the slip history (red line in (b)). Graphs (c) and (d) show the slip rate variability (SRV) calculated for both the actual and the modelled slip histories respectively. SRV is defined (Cowie et al., 2012) as the standard deviation of the slip rates (SR_1, SR_2, etc.) measured over a fixed time window divided by the average slip rate (SR_{ave}). SRV is calculated for different window lengths and using two different methods (a sliding window (black dots and lines) versus consecutive time windows (grey dots and lines in (c)). The sliding window method gives more stable results and shows that for window lengths > 2500 years the SRV is much less sensitive to window length. In this study we use a window of length 3000 years, consistent with previous published work in central Italy (Cowie et al., 2012). In (d) the SRV values for the five highest likelihood fits are shown (grey lines) plus the inferred Scarp Age (SA) in each case. The maximum likelihood fit (see black dots and line in (d)) captures the SRV value (= 0.45) of the actual slip history used to generate the synthetic data and is well-resolved (SRV > 0.2; see Fig. S 4.2.2).

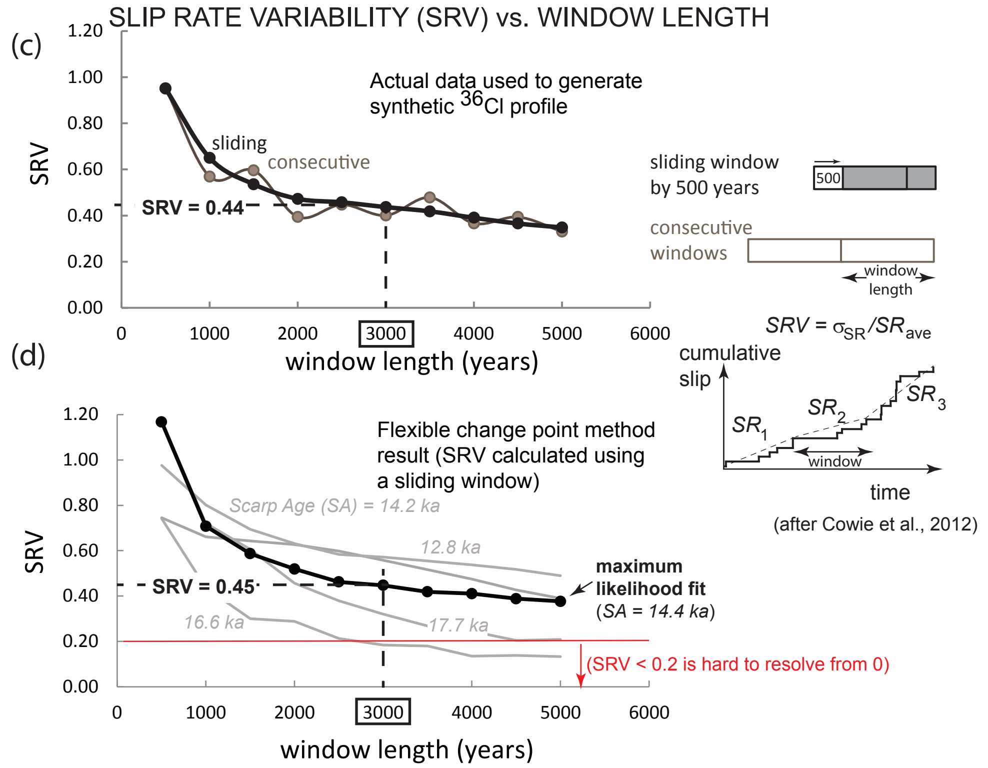

.

Section 4.4 Summary of site specific model parameters (Table S 4.4.1), Bayesian parameters (Table S 4.4.3) and modelling results (Tables S 4.4.2 & S 4.4.4)

Table S 4.4.1 Site specific parameters used in modelling

Site ID	Elevation (m)	Latitude	α	β	γ	Scarp Height* (cm)	Trench* (cm)	HW density (g/cm^3)	Slip/event (cm)#
FIAM	1150	42.3	23	42	33	2705	115	1.5	60
MA3	1255	42.12	30	42	36	1605	395	1.5	30
TREM	1020	42.05	25	57	33	1020	190	1.5	20
PARA	1268	41.99	33	62	33	900	195	1.5	30
SSB	1208	41.95	32	65	32	420	155	1.5	20
GDM	1050	41.95	37	55	40	1900	195	1.88	60
FRAT	1484	41.93	25	53	28	1570	130	1.5	50
PESC	1349	41.83	18	70	39	580	90	1.6	20

* measured in the plane of the fault; #Wells and Coppersmith(1994) scaling corrected for distance from nearest fault tip

HW density = average density of hanging wall colluvial wedge

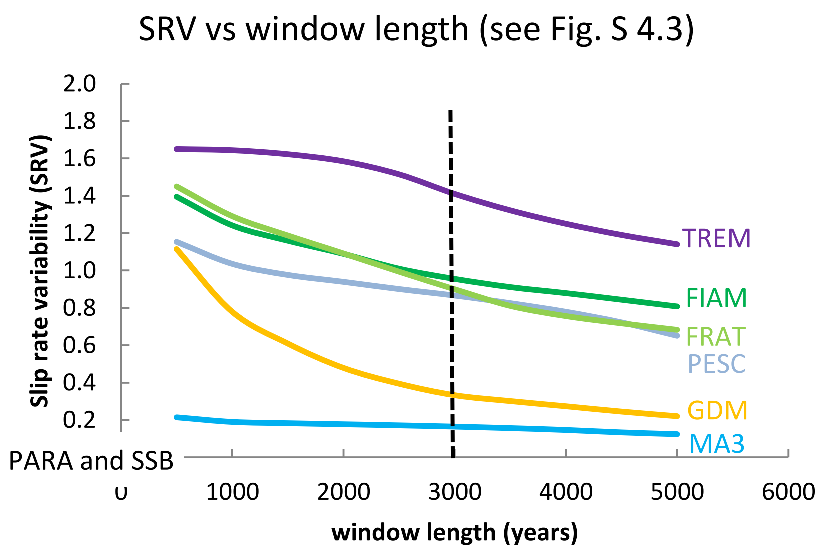

Table S 4.4.2 Model results: Average slip rates, SRV and SR_{max}

	#SR_{mean}	§SR_{mean}	¥SRV	¤SR_{max}
FIAM	1.8 (+0.45/-0.3)	1.9	1.0	6
MA3	1.07 (+0.27/-0.18)	1.55	≥0.2*	1.8
TREM	0.68 (+0.17/-0.11)	0.32	1.4	2
PARA	0.6 (+0.15/-0.1)	0.54	0.0	0.54
SSB	0.26 (+0.06/-0.04)	0.2	0.0	0.2
GDM	1.27 (+0.32/-0.21)	0.98	0.3	2.2
FRAT	1.05 (+0.26/-0.17)	0.86	0.9	2.5
PESC	0.39 (+0.1/-0.06)	0.25	0.9	0.7

Average slip rate in mm/yr; range in brackets (scarp height ÷ 15±3 kyrs)

§ Average slip rate in mm/yr obtained from maximum likelihood fits to ^{36}Cl measurements (see Table S 4.4.4)

¥ Slip Rate Variability (SRV) obtained from maximum likelihood fits to ^{36}Cl measurements using a 3000 year sliding window (SRV defined in Fig. S 4.3; shaded: SRV < 0.2); *slip history from Schlagenhauf et al. (2010): SRV = 0.4.

¤ Maximum short term slip rate (mm/yr) (over a 3000 year time window)

Table S 4.4.3 Parameters used in Bayesian Modelling (see section 4.1)

				Scarp Age prior (yr)¥		Elapsed Time prior (yr)§	
**Site ID**	**CP_1***	**CP_2***	**Elapsed time (yr)**	**Mean**	**Std dev**	**Mean**	**Std dev**
FIAM	1515	2355	665 (fixed)	15000	2500		
MA3	1415		**$**	15000	2500		
TREM	380	390	**$**	15000	2500		
PARA			**$**	15000	2500		
SSB†			**$**	15000	2500		
GDM	640	2099	Estimated	15000	2500	800	200
FRAT (FIXED CP)	880	881	Estimated	15000	2500	3000	1000
FRAT (FLEX CP)			Estimated	15000	2500	3000	1000
PESC			Estimated	15000	2500	6000	1000

***** = height of change point in cm, includes trench depth

† = slip history estimated using optimisation approach only

¥ = Scarp Age (SA) prior normal distribution based on the onset of the demise of the LGM: 12 – 18 ka (Giraudi & Frezzoti, 1997)

§ = Elapsed Time (ET) prior for site GDM chosen to be consistent with Michetti et al. (1996); for site FRAT the ET prior was based on initial trial and error modelling of the ^{36}Cl profile which indicated an elapsed time of a few thousand years was required to explain the change in gradient of ^{36}Cl profile at the ground surface (see Fig. S. 4.2.3(b)); for site PESC a long ET is also indicated by the ^{36}Cl profile shape (see Fig. S 4.2.3(b)) and the prior was chosen to span the timing of onset of the most recent activity on the Frattura fault immediately across strike where site FRAT is located. Normal distributions used to define priors.

$ = the elapsed time of the most recent slip accrued at these sites (quoted in Table 4.4.4) is a function of the slip size assumed, i.e., it is equal to the age of either CP1 (or SA if no change in slip rate is inferred) divided by the number of slip events required to generate the height of CP1 (or SA if no change in slip rate is inferred).

Table S 4.4.4 Highest likelihood age arrays (in years) for each site

FIAM	MA3	TREM	PARA	SSB	GDM	FRAT (FIXED CP)	FRAT (FLEX CP)	PESC
12332.0	10270.0	23253.0	16552.0	20750	17290.0	19260.0	17991.0	22525.0
11320.0	10033.0	23174.0	16000.0	19761.9	16615.0	18832.0	17854.0	22207.0
10308.0	9795.9	23096.0	15448.3	18773.8	15940.0	18404.0	17718.0	21890.0
9296.4	9558.8	23017.0	14896.6	17785.7	15265.0	17975.0	17581.0	21572.0
8284.6	9321.8	22939.0	14344.8	16797.6	14591.0	17547.0	17445.0	21255.0
7272.7	9084.7	22860.0	13793.1	15809.5	13916.0	17119.0	17308.0	20937.0
6260.9	8847.7	22781.0	13241.4	14821.4	13241.0	16691.0	17172.0	20620.0
5249.0	8610.6	22703.0	12689.7	13833.3	12566.0	16262.0	17035.0	20302.0
5014.8	8373.6	22624.0	12137.9	12845.2	11891.0	15834.0	16899.0	19985.0
4780.6	8136.5	22546.0	11586.2	11857.1	11216.0	15406.0	16762.0	19667.0
4546.4	7899.5	22467.0	11034.5	10869.0	10541.0	14978.0	16626.0	19408.0
4312.1	7662.4	22389.0	10482.8	9880.9	9866.4	14549.0	16489.0	19148.0
4077.9	7425.4	22310.0	9931.0	8892.8	9191.5	14121.0	16353.0	18889.0
3843.7	7188.3	22231.0	9379.3	7904.7	8516.6	13693.0	16216.0	18630.0
3609.5	6951.3	22153.0	8827.6	6916.6	7841.8	13265.0	16080.0	18371.0
3375.3	6714.2	22074.0	8275.9	5928.5	7166.9	12836.0	12168.0	18111.0
3141.1	6477.2	21996.0	7724.1	4940.4	6492.0	12408.0	8256.7	16420.0
2906.9	6240.1	21917.0	7172.4	3952.3	5817.1	11980.0	4345.1	14728.0
2672.6	6003.1	21839.0	6620.7	2964.2	5142.3	4930.0	4284.0	13036.0
2438.4	5766.0	21760.0	6069.0	1976.1	4467.4	4796.1	4222.8	11345.0
2204.2	5529.0	21681.0	5517.2	988.0	3792.5	4662.1	4161.7	9653.0
1970.0	5366.4	21603.0	4965.5	0.0	3117.6	4528.2	4100.5	9164.8
1913.3	5203.8	21524.0	4413.8		2442.8	4394.3	4039.3	8676.6
1856.5	5041.1	21446.0	3862.1		1767.9	4260.4	3978.2	8188.5
1799.8	4878.5	21367.0	3310.3		1093.0	4126.4	3917.0	7700.3
1743.0	4715.9	21289.0	2758.6		1035.1	3992.5	3855.8	7212.1
1686.3	4553.3	21210.0	2206.9		977.3	3858.6	3794.7	6723.9
1629.6	4390.7	21131.0	1655.2		919.4	3724.6	3733.5	6235.8
1572.8	4228.1	21053.0	1103.4		861.6	3590.7	3672.4	5747.6
1516.1	4065.4	20974.0	551.7		803.7	3456.8	3611.2	0.0
1459.3	3902.8	14415.0	0.0		745.9	3322.9	3550.0	
1402.6	3740.2	7855.5			688.0	3188.9	3488.9	
1345.9	3577.6	7444.7			0.0	3055.0	0.0	
1289.1	3415.0	7033.9				0.0		
1232.4	3252.4	6623.1						
1175.7	3089.7	6212.2						
1118.9	2927.1	5801.4						
1062.2	2764.5	5390.6						
1005.4	2601.9	4979.8						
948.7	2439.3	4569.0						
892.0	2276.6	4158.2						
835.2	2114.0	3747.3						
778.5	1951.4	3336.5						
721.7	1788.8	2925.7						
665.0	1626.2	2514.9						
0.0	1463.6	2104.1						
	1300.9	1693.3						
	1138.3	1282.4						
	975.7	871.6						
	813.1	460.8						
	650.5	50.0						
	487.9	0.0						
	325.2							
	162.6							
	0.0							

Grey shade in top row indicates Scarp Age (SA) estimated for each site using our modelling approach. The mean SA = 17.8 ± 4.3 ka (9 independent estimates) represents a regional estimate of the onset of bedrock scarp preservation based on our modelling of the fault plane samples. Our upper slope cosmogenic sample at one site (FIAM) gave a slope stabilization age of 17.0 +1.7/-1.8 ka (corrected for LGM erosion rate, see Section 4.1.1) and lies within 1 standard deviation of this regional estimate.

Bayesian Modelling - Site FIAM (Section 3, Table S 4.4.1 and Figure 1 (main text) indicate location and summarize site specific modelling parameters)

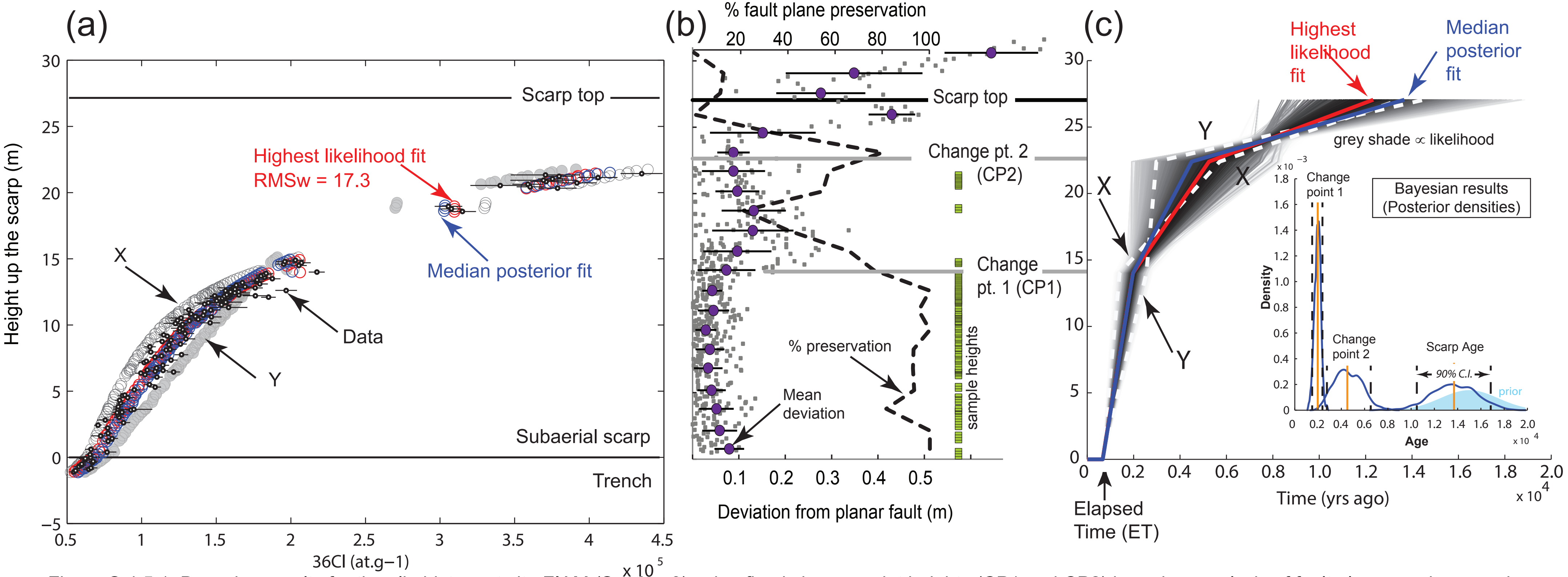

Figure S 4.5.1. Bayesian results for the slip history at site FIAM (Section 3) using fixed change point heights (CP1 and CP2) based on analysis of fault plane roughness using terrestrial LiDAR data (see Fig. S 4.5.2): (a) modelled 36Cl profiles compared to the data, (b) roughness, fault plane preservation and sample ladders, and (c) fault slip versus time over the last ~20 kyrs derived from our Bayesian approach plotted as height on the scarp versus time in years so that 0 = present day.
Age information on the demise of the LGM (12 - 18 ka) and the associated reduction in erosion rates that led to scarp preservation in this area (Tucker et al., 2011), is included as prior information on scarp age (SA) (blue filled pdf in inset in part (c)). Fits X and Y indicate fits that approximately correspond to the 90% credible intervals (C.I.) on ages for CP1, CP2 and SA. An abrupt change in the shape of the 36Cl profile at the base of the scarp at this site indicates that there is an elapsed time (ET) of several hundred years since the last significant accumulation of slip (see synthetic data Fig. S 4.2.3(b)). The non-zero elapsed time that we use to constrain the modelling for this site is the timing of the 1349 AD earthquake (we set ET = 665 yrs), which historical records strongly suggest ruptured this fault (Guerrieri et al., 2002; Galli and Naso, 2009).
Our age estimate for CP1 is 1973 yrs ago (+404/-479 yrs., 90% C.I.), for CP2 is 4516 yrs ago (+2015/-1753 yrs., 90% C.I.) and for SA 13.70 ka (±0.32 ka., 90% C.I.). These age estimates indicate that between 1349 AD and approximately 2000 years ago (i.e., from Roman times to the end of the Middle Ages) displacement accumulation occurred very rapidly, and the slip rate deviated significantly from the Holocene-averaged rate (~1.8 mm/yr) on this fault but that this rapid phase was preceded by a period of lower than average rates of slip, particularly between ~4.5 ka and 13.7 ka (~0.6 mm/yr). **SRV = 1.0** is estimated for the highest likelihood slip history at this site (Table S 4.4.2). Including the second change point (CP2) leads to a better fit to the topmost samples, i.e., above height = 18 m. If CP2 is not included then the RMSw increases from 17.3 to 19.32 and the SA we then infer is only 9.5 ka which lies outside the the 12 - 18 ka age range of the LGM demise and also inconsistent with the age of the stablisation of the upper slope at this site (17.0 +1.7/-1.8 ka; see Section 4.1.1). In addition we ran models with ± 0.5 m variation in the heights of CP1 and CP2 and there was no significant difference in the results shown here. In Fig. S 4.5.3 we present the results of sensitivity tests which we performed to test different exhumation scenarios at this site.

Variations in fault plane roughness used to constrain change points at Site FIAM

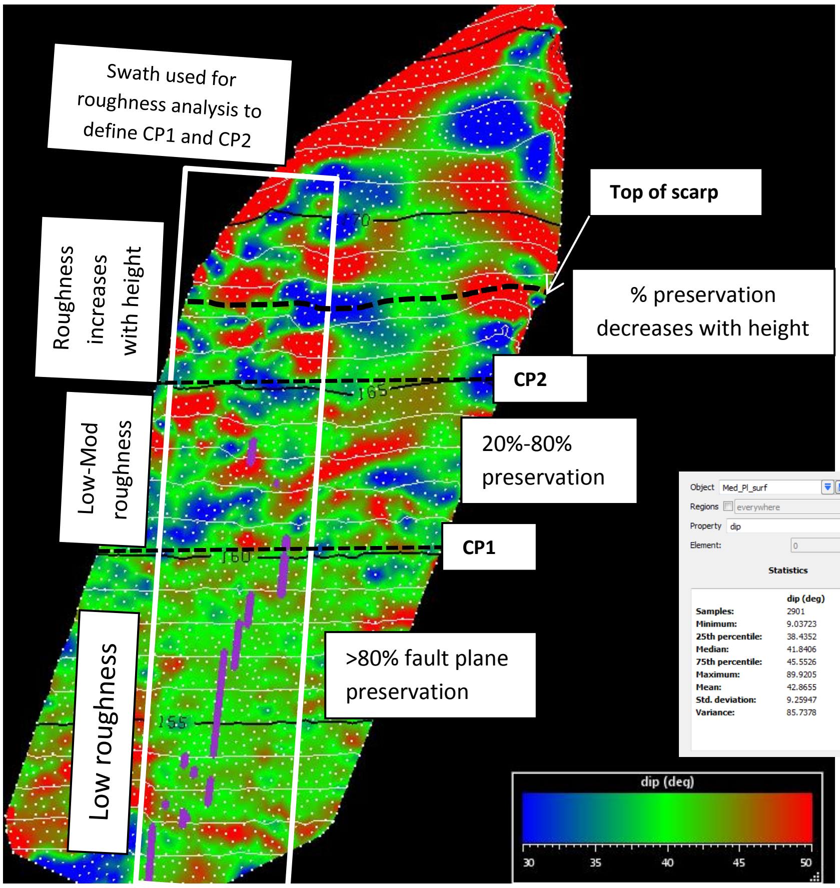

Fig. S 4.5.2. Swath of terrestrial LiDAR data (5m wide) along sample ladder for which fine-scale variations in fault plane roughness and fault plane preservation were extracted (see Fig. S 4.5.1b). Colour scale indicates deviation from planar. Only areas of the fault surface with 100% preservation, low surface roughness and evidence for tectonic striae were sampled for cosmogenic analysis (see sample ladder, purple, located in bright green areas where fault dip = 42°).

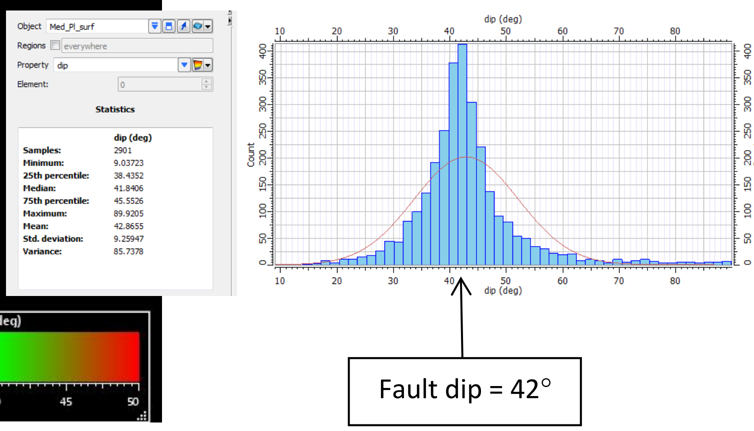

Below CP1 the fault plane is consistently low in roughness (mean deviation < a few cm) and preservation is mostly 90-100%. Between CP1 and CP2 roughness changes abruptly (mean deviation of ~10 cm) but shows no systematic increase with height; the degree of fault plane preservation is more variable in this zone (see red and blue areas). Above CP2 roughness and % preservation both show a clear dependence on height consistent with progressive erosion of the scarp top. Areas of the fault plane where blocks have been recently plucked (e.g., bottom left) are easy to distinguish by angular morphology.

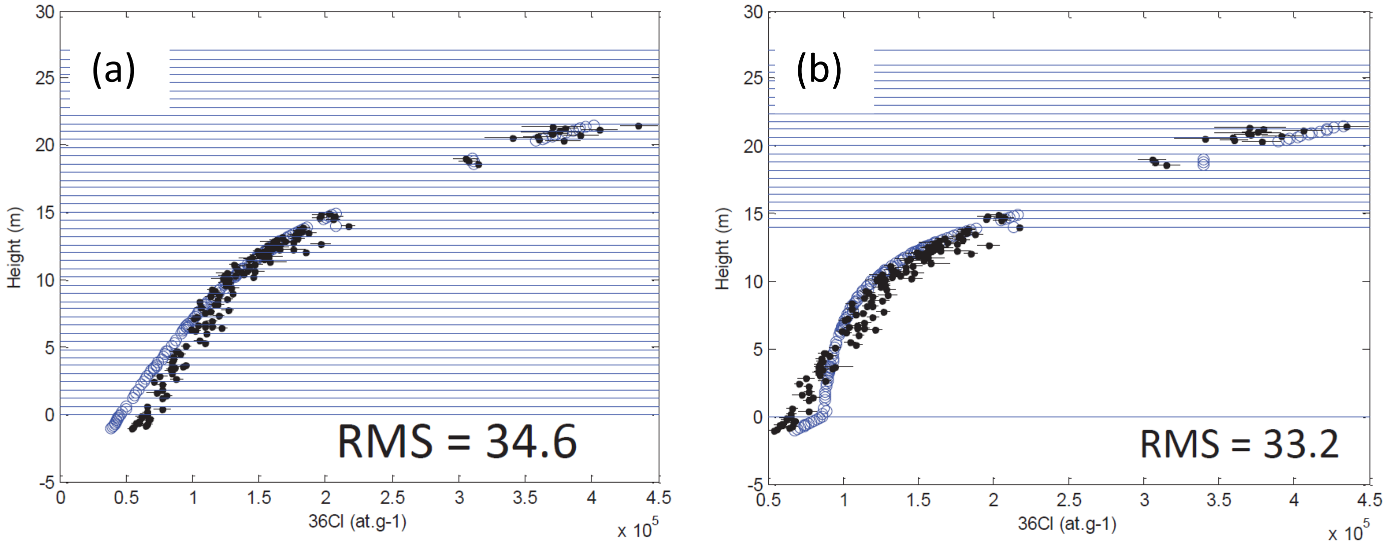

Fig. S 4.5.3. Sensitivity tests: (a) Zero elapsed time used instead of 665 years (corresponding to earthquake of 1349 widely believed to have ruptured the Fiamignano fault: Guerrieri et al., 2002; Galli and Naso, 2009). (b) Single exhumation event by a non-tectonic process (i.e., a landside) for the smoother well-preserved portion of the fault below CP1 (see Fig. S 4.5.2). Both scenarios lead to significantly worse fits to the data; compare with our lowest RMS (highest likelihood) fit using a variable fault slip rate (RMSw=17.1) shown in Fig. S 4.5.1.

Bayesian Modelling - Site MA3 (Section 3, Table S 4.4.1 and Figure 1 (main text) indicate location and summarize site specific modelling parameters)

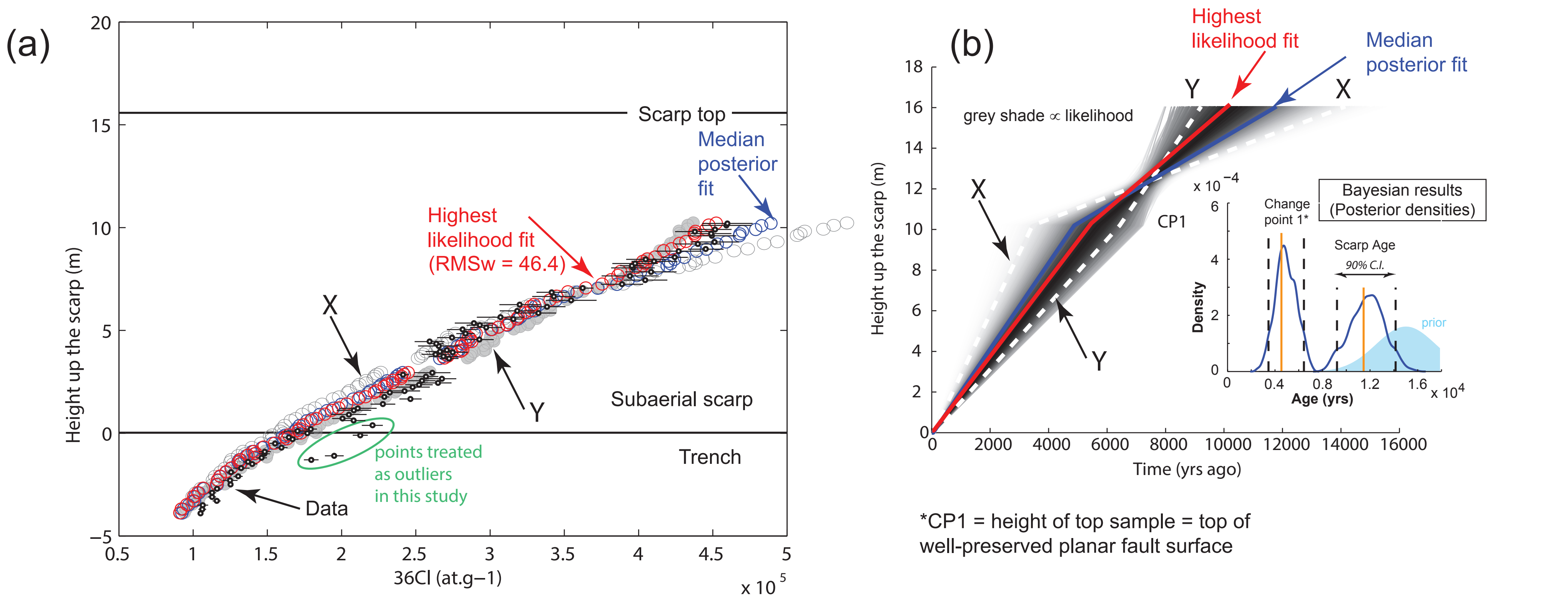

*CP1 = height of top sample = top of well-preserved planar fault surface

Figure S 4.5.4. Bayesian results for the slip history at site MA3 (data and model parameters from Schlagenhauf et al., 2010). Here we solve for one fixed change point (CP1) based on the extent of the sample ladder which corresponds to the high % preservation portion of the fault (Schlagenhauf et al., 2010): (a) modelled ^{36}Cl profiles compared to the data, (b) fault slip versus time since 18 ka derived from Bayesian approach plotted as height up the scarp versus time in years ago. Fits X and Y indicate fits that approximately correspond to the 90% credible intervals (C.I.) on ages for CP1 and Scarp Age (SA). Our single change point approach allows us to search for the 1st order features of the slip history rather than details that may be artefacts caused by hanging-wall sedimentation/erosion during the Holocene (see Galli et al. 2012). We estimate **SA = 11.76 ka (+2.4/-2.6 kyrs 90% C.I)** for this site, which is at the lower bound of the prior that we use for SA based on the demise of the LGM in this region (12 - 18 ka; Giraudi and Frezzoti, 1997). Our estimate for **the age of CP1 is 4914 yrs ago (+1545/-1465 years 90% C.I)**. Our results suggest only subtle variations in slip rate over the last ~12 ka, no significant elapsed time (ET) and **SRV = 0.2 (Table S 4.4.2).** Galli et al. (2012) present paleoseismological observation indicating that this fault ruptured in earthquakes in 508 AD and 1915, consistent with the interpretation that ET is short, whereas Schlagenhauf et al. (2010) estimated that ET ≈ 1500 yrs. Our modelling approach favours simpler fits and minimum values for SRV; the slip history published by Schlagenhauf et al. (2010) for this site is characterised by SRV = 0.4. **The average rate of slip is estimated to be 1.6 mm/yr (+0.3/-0.2 mm/yr 90% C.I.).** Schlagenhauf et al. (2010; see their supplementary file) estimated an average rate of 1.5 mm/yr over the last 10 ka (RMSw 35.6) although they refer to this as a creep rate and it is not clear what assumptions/parameters were used to model the creep.

Bayesian Modelling - Site TREM (Section 3, Table S 4.4.1 and Figure 1 (main text) indicate location and summarize site specific modelling parameters)

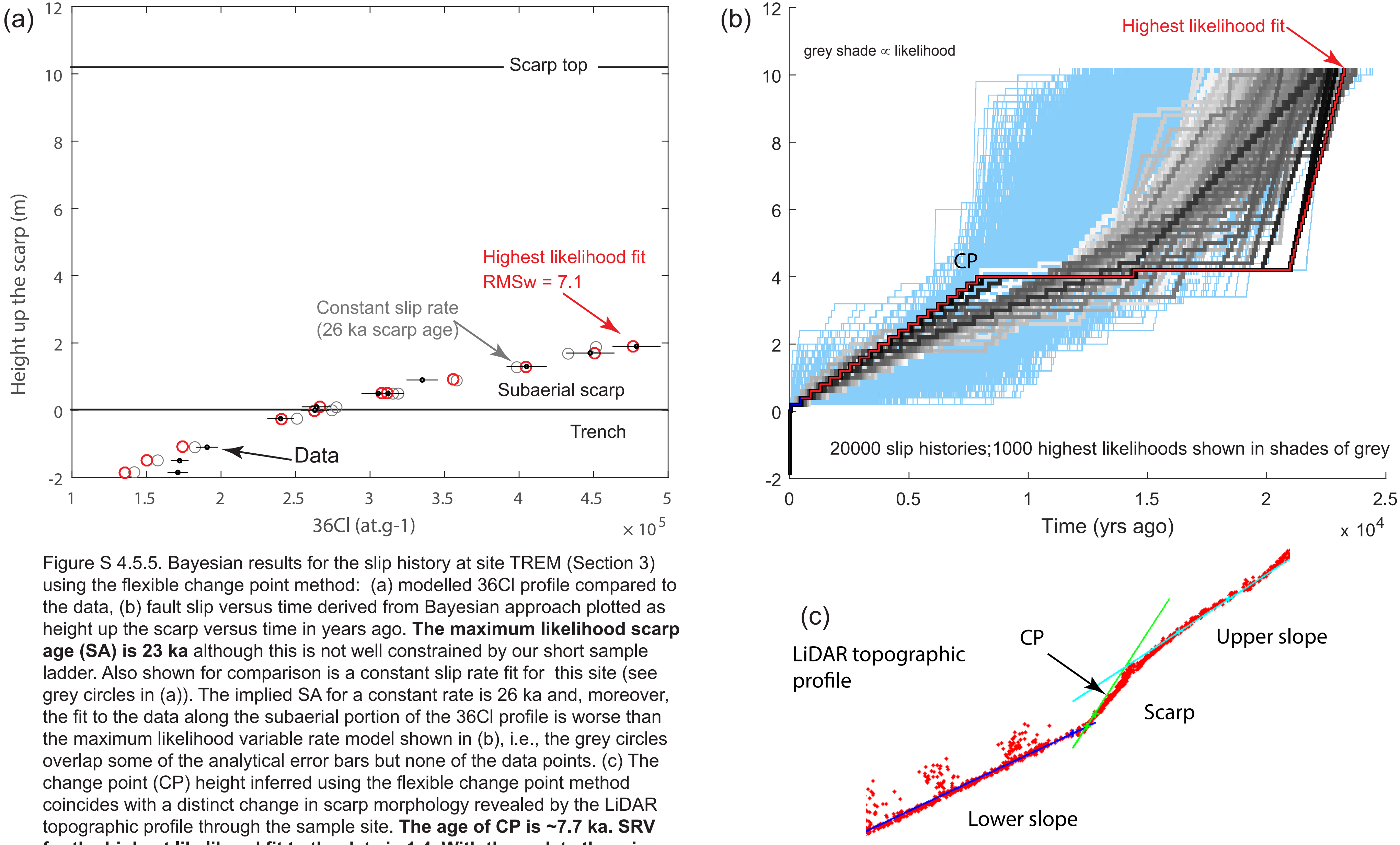

Figure S 4.5.5. Bayesian results for the slip history at site TREM (Section 3) using the flexible change point method: (a) modelled 36Cl profile compared to the data, (b) fault slip versus time derived from Bayesian approach plotted as height up the scarp versus time in years ago. **The maximum likelihood scarp age (SA) is 23 ka** although this is not well constrained by our short sample ladder. Also shown for comparison is a constant slip rate fit for this site (see grey circles in (a)). The implied SA for a constant rate is 26 ka and, moreover, the fit to the data along the subaerial portion of the 36Cl profile is worse than the maximum likelihood variable rate model shown in (b), i.e., the grey circles overlap some of the analytical error bars but none of the data points. (c) The change point (CP) height inferred using the flexible change point method coincides with a distinct change in scarp morphology revealed by the LiDAR topographic profile through the sample site. **The age of CP is ~7.7 ka. SRV for the highest likelihood fit to the data is 1.4. With these data there is no significant ET resolved at this site, i.e. ET < a few hundred years.** A relative high slip variability (Table S 4.4.2) is consistent with the highly oblique orientation of this fault (NE-SW) relative to the regional strike (NW-SE) of the overall fault array in this area but SRV is not well constrained by these data.

Bayesian Modelling - Site PARA (Section 3, Table S 4.4.1 and Figure 1 (main text) indicate location and summarize site specific modelling parameters)

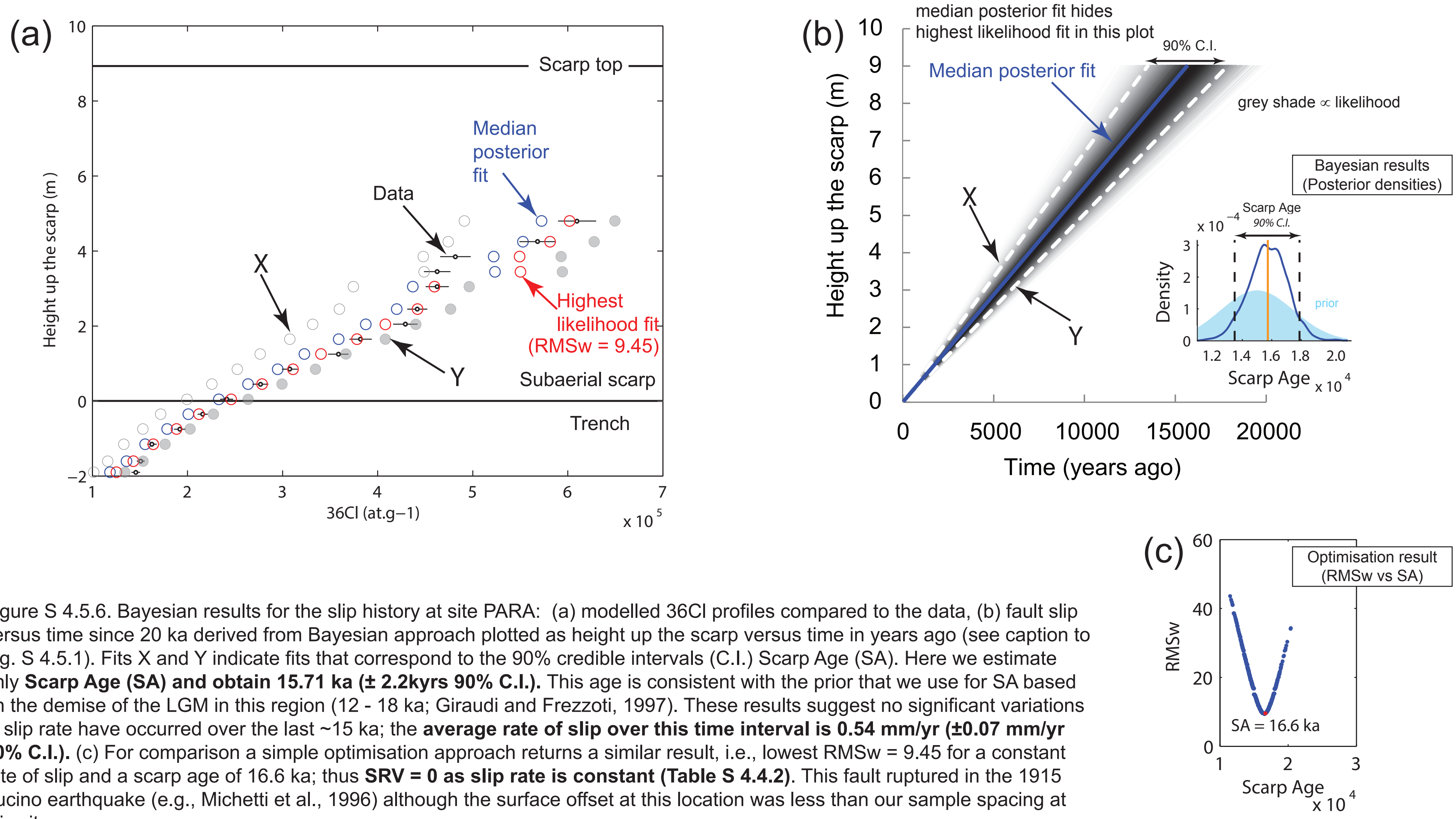

Figure S 4.5.6. Bayesian results for the slip history at site PARA: (a) modelled 36Cl profiles compared to the data, (b) fault slip versus time since 20 ka derived from Bayesian approach plotted as height up the scarp versus time in years ago (see caption to Fig. S 4.5.1). Fits X and Y indicate fits that correspond to the 90% credible intervals (C.I.) Scarp Age (SA). Here we estimate only **Scarp Age (SA) and obtain 15.71 ka (± 2.2kyrs 90% C.I.).** This age is consistent with the prior that we use for SA based on the demise of the LGM in this region (12 - 18 ka; Giraudi and Frezzoti, 1997). These results suggest no significant variations in slip rate have occurred over the last ~15 ka; the **average rate of slip over this time interval is 0.54 mm/yr (±0.07 mm/yr 90% C.I.).** (c) For comparison a simple optimisation approach returns a similar result, i.e., lowest RMSw = 9.45 for a constant rate of slip and a scarp age of 16.6 ka; thus **SRV = 0 as slip rate is constant (Table S 4.4.2)**. This fault ruptured in the 1915 Fucino earthquake (e.g., Michetti et al., 1996) although the surface offset at this location was less than our sample spacing at this site.

Optimisation modelling - Site SSB (Section 3, Table S 4.4.1 and Figure 1 (main text) indicate location and summarize site specific modelling parameters)

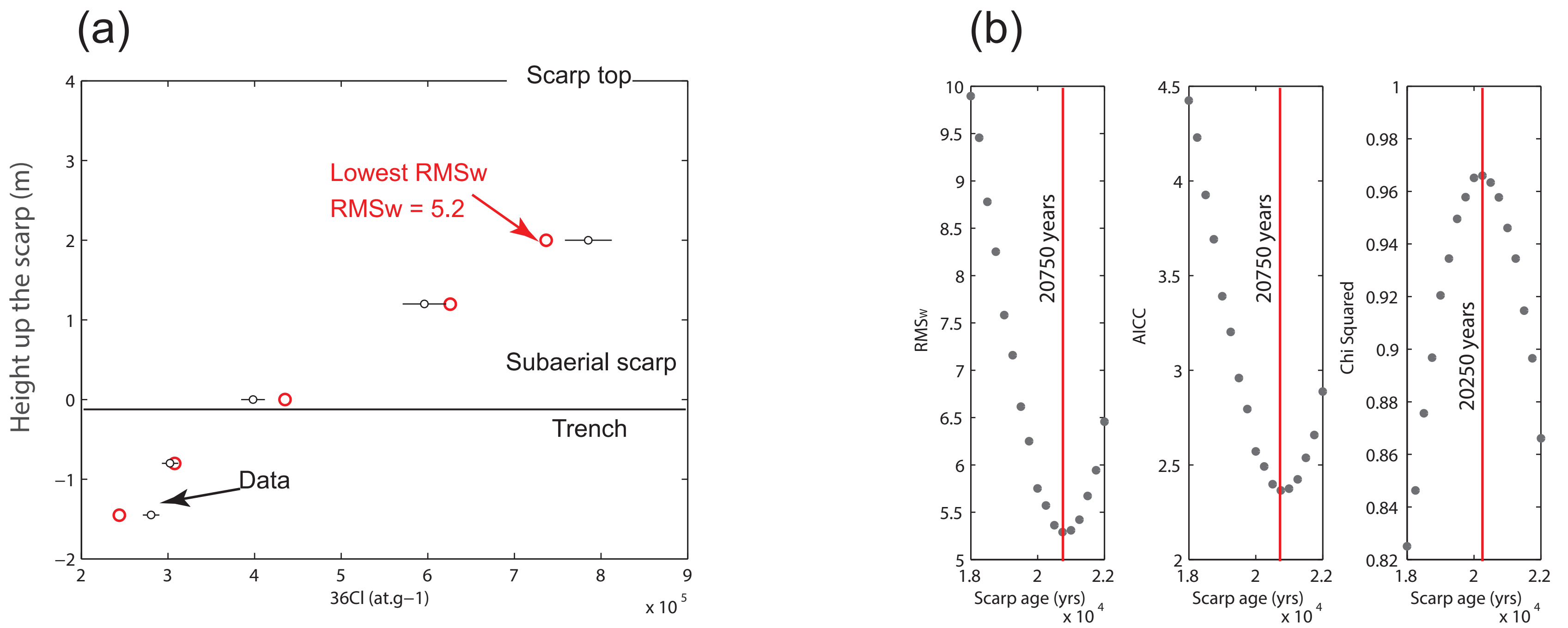

Figure S 4.5.7. Optimisation result for site SSB. As there are only 5 samples at this site we use a simple optimisation approach to fit a constant slip rate to this data set. (a) 36Cl profile for the lowest RMSw fit to the measurements, (b) RMSw, AICC and Chi-squared values for constant slip rate models as a function of scarp age (SA). **The SA implied by the lowest RMSw constant slip rate model is ~20 ka** which agrees to within two standard deviations the mean scarp age of 15 ka used as prior information in the modelling of the other sites. **The slip rate is 0.2 mm/yr and SRV = 0** as the slip rate is constant for the fit to these data (Table S 4.4.2 & Table S 4.4.4).

Bayesian Modelling - Site GDM (Section 3, Table S 4.4.1 and Figure 1 (main text) indicate location and summarize site specific modelling parameters)

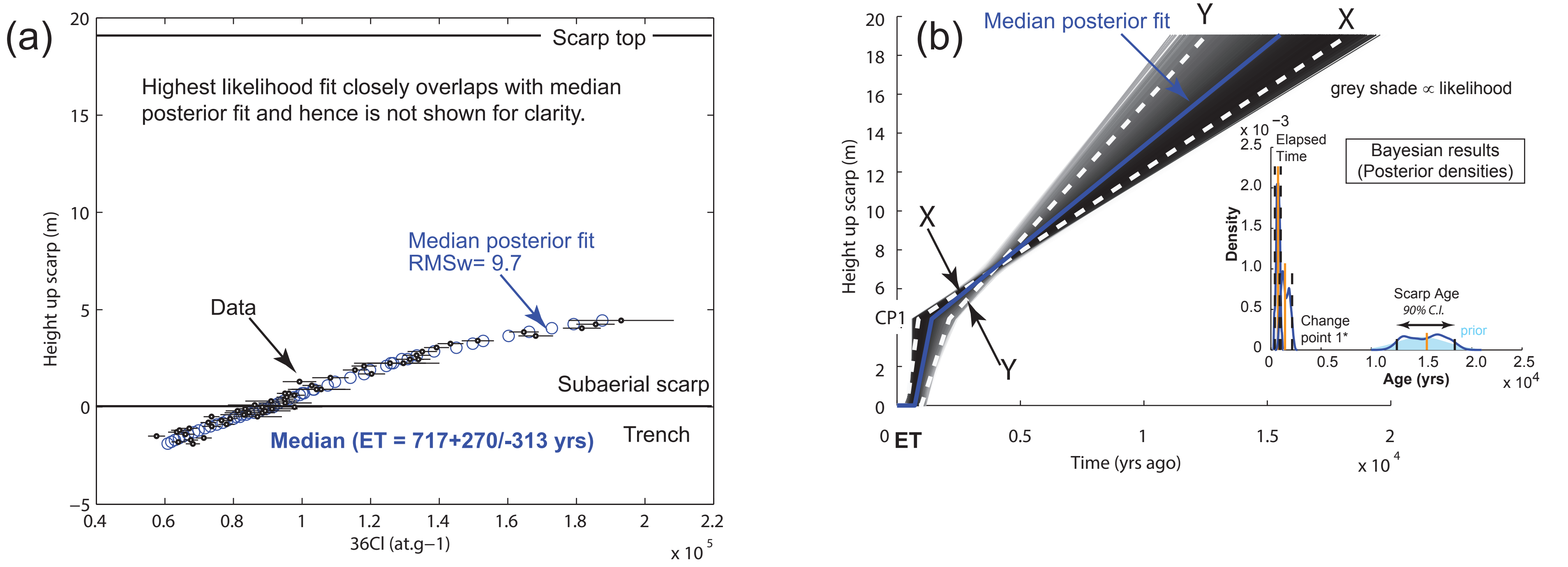

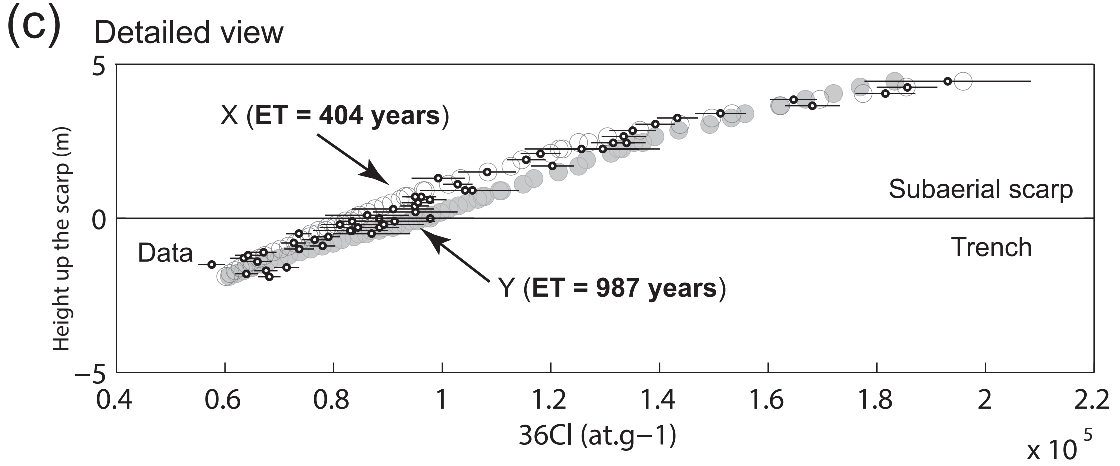

*CP1 = height of top sample = top of pristine planar fault surface

Our estimate for **Elapsed Time (ET) is 717 (+270/-313 yrs., 90% C.I.) and the age of CP1 = 1400 yrs ago (+720/-530 years 90% C.I)**. We also conducted model runs with a uniform prior on (ET) and found that if ET is less than ≈ 700 ± 300 years then RMSw increases to >20.

Figure S 4.5.8. Bayesian results for the slip history at site GDM (Section 3) using one fixed change point height (CP1) based on extent of smoothest/highest % preservation portion of sampled fault plane: (a) modelled 36Cl profiles compared to the data, (b) fault slip versus time since 20 ka derived from Bayesian approach plotted as height up the scarp versus time in years ago. (c) Shows a detailed view of the sample ladder and the 90% C.I. fits (X and Y) used to constrain Elasped Time (ET). Here we solved for both ET as well as the age of CP1 and scarp age (SA). The results show that SA closely follows the prior (12 - 18 ka), a consequence of the limited extent of the sample ladder. The cosmogenic data provide evidence for a period of rapid slip between approximately Roman times and the end of the Middle Ages similar to that which we inferred for the slip history at site FIAM and also agrees with the paleoseismic study on the Fucino fault published by Michetti et al. (1996) which found evidence for at least two large events in historical times (probably 801 A.D. and between 1000-1349 A.D.). Surface slip in the 1915 earthquake was ≤ sample spacing at this site and thus not resolved. We calculate **SRV = 0.3** for the maximum likelihood slip history at this site (Table S 4.4.2).

Bayesian Modelling - Site FRAT (Section 3, Table S 4.4.1 and Figure 1 (main text) indicate location and summarize site specific modelling parameters)

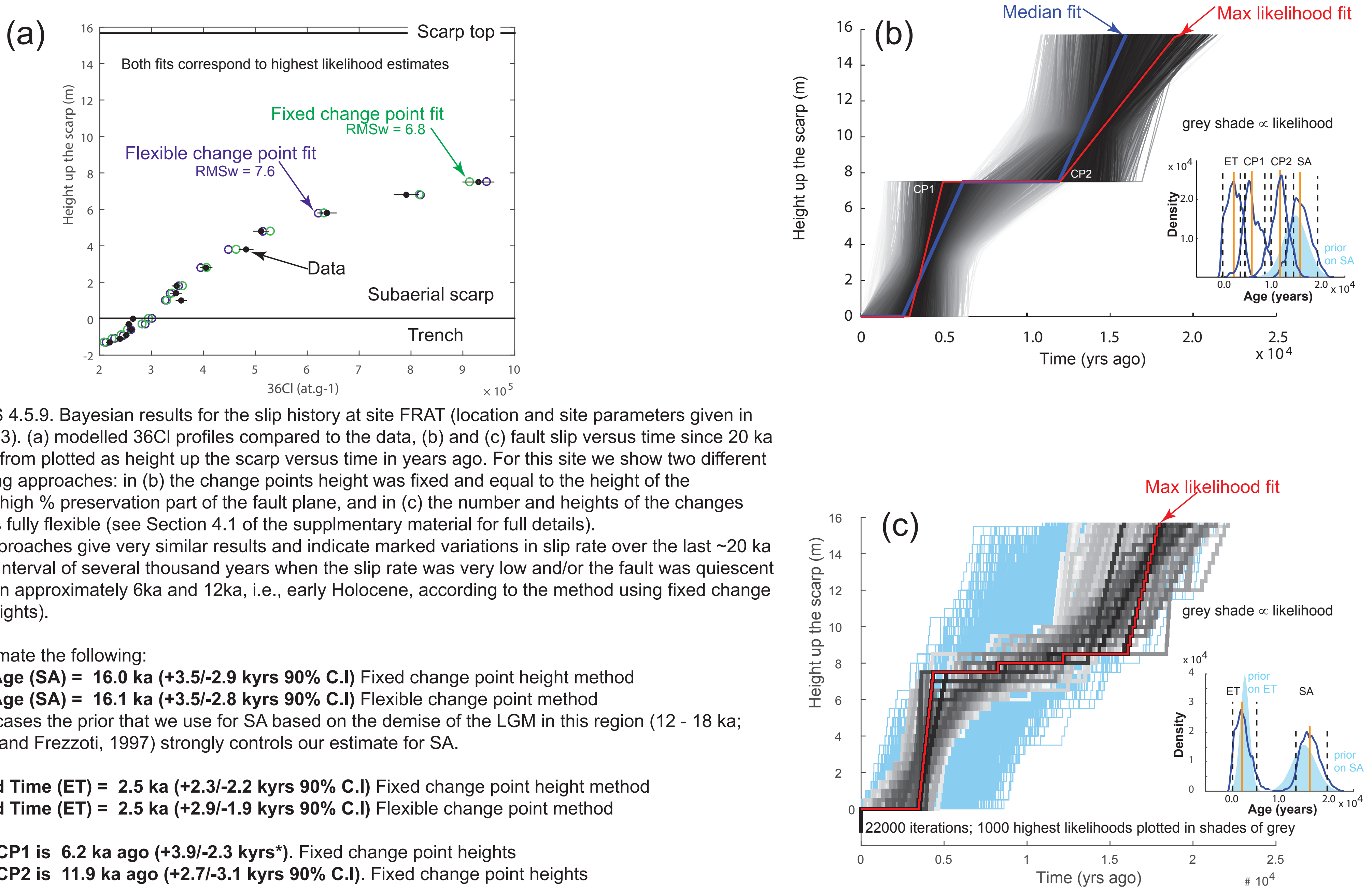

Figure S 4.5.9. Bayesian results for the slip history at site FRAT (location and site parameters given in Section 3). (a) modelled 36Cl profiles compared to the data, (b) and (c) fault slip versus time since 20 ka derived from plotted as height up the scarp versus time in years ago. For this site we show two different modelling approaches: in (b) the change points height was fixed and equal to the height of the smooth/high % preservation part of the fault plane, and in (c) the number and heights of the changes points is fully flexible (see Section 4.1 of the supplmentary material for full details).
Both approaches give very similar results and indicate marked variations in slip rate over the last ~20 ka with an interval of several thousand years when the slip rate was very low and/or the fault was quiescent (between approximately 6ka and 12ka, i.e., early Holocene, according to the method using fixed change point heights).

We estimate the following:
Scarp Age (SA) = 16.0 ka (+3.5/-2.9 kyrs 90% C.I) Fixed change point height method
Scarp Age (SA) = 16.1 ka (+3.5/-2.8 kyrs 90% C.I) Flexible change point method
in both cases the prior that we use for SA based on the demise of the LGM in this region (12 - 18 ka; Giraudi and Frezzoti, 1997) strongly controls our estimate for SA.

Elapsed Time (ET) = 2.5 ka (+2.3/-2.2 kyrs 90% C.I) Fixed change point height method
Elapsed Time (ET) = 2.5 ka (+2.9/-1.9 kyrs 90% C.I) Flexible change point method

Age of **CP1 is 6.2 ka ago (+3.9/-2.3 kyrs*)**. Fixed change point heights
Age of **CP2 is 11.9 ka ago (+2.7/-3.1 kyrs 90% C.I)**. Fixed change point heights
* not fully converged after 22000 iterations

Highest likelihood slip histories: **SRV = 0.9 (fixed CPs) and 1.3 (flex CPs) Table S 4.4.2**. If a uniform prior on SA is used the data can be fit without CP2 and then SA = 28ka, which is nearly double the expected age based on the onset of scarp preservation/end of the LGM (for this scenario SRV = 0.92).

Bayesian Modelling - Site PESC (Section 3, Table S 4.4.1 and Figure 1 (main text) indicate location and summarize site specific modelling parameters)

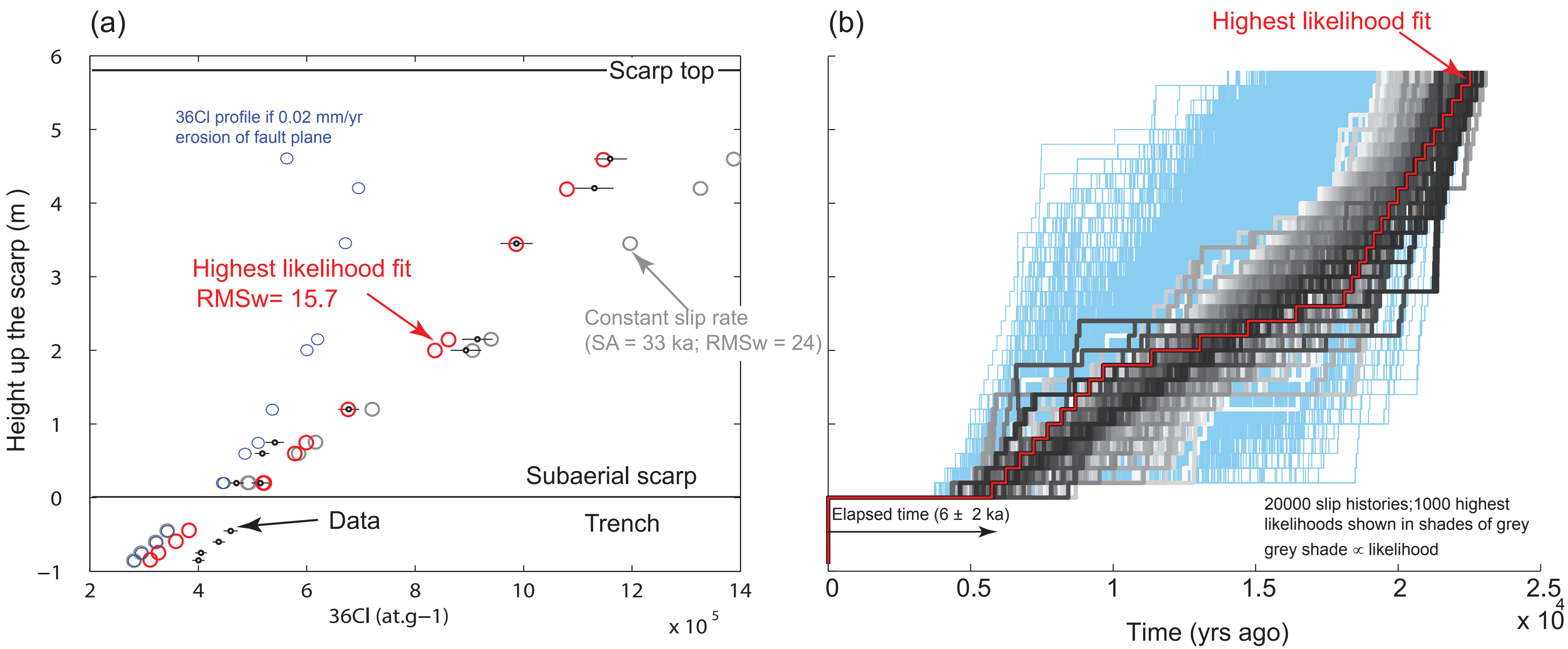

Figure S 4.5.10. Bayesian results for the slip history at site PESC (Section 3) using flexible change point method: (a) modelled ^{36}Cl profiles compared to the data, (b) fault slip versus time since 25 ka derived from Bayesian approach plotted as height up the scarp versus time in years ago.

The prior on the ET was set at 6 ± 2 ka yrs ago. **The maximum likelihood fit implies a scarp age (SA) = 22.5 ka**. Inherited ^{36}Cl was included in the modelling at this site to take into account the relatively low erosion rate compared to fault slip rate during the LGM indicated by a less well-developed planar upper slope at this site (Section 3). Using the formula of Tucker et al. (2011) and the measured site geometry we estimated that the inherited ^{36}Cl is equivalent to 6000yrs of preexposure at this site. The **ET for the highest likelihood fit = 5.7 kyrs** and a conservative estimate of **SRV = 0.9** (using a 3000 year sliding window; Table S 4.4.2).
If inherited ^{36}Cl is not included the estimated SA = 29 ka, which signifinantly older than the demise of the LGM (12-18 ka) and onset of scarp preservation due to reduced Holocene erosion rates (Tucker et al. 2011), but similar results are obtained for ET = 6.7 kyrs and SRV = 1.3.
Thus a long ET and high SRV are robust conclusions obtained from our modelling of this site: a constant slip rate model does not fit the data well (RMSw increases from 16 to 24) and implies a scarp age of 33 kyrs. Some erosion of the fault plane during the Holocene is evident at this site (see site charactersation photographs in Section 3) but we sampled from the least degraded areas. The measured profile of ^{36}Cl concentration increases with height, as at the other sites, whereas even a modest erosion rate (0.02mm/yr) would predict little increase or even a decreasing ^{36}Cl concentration with height (see blue circle in (a)). Finally, even though the subaerial portion of the ^{36}Cl profile may be somewhat modified by erosion the subsurface ^{36}Cl profile at this site indicates that this fault has a very long ET, i.e., several 1000 years, consistent with the lack of evidence for historical ruptures on this fault.

Table S 4.6 Parameters used in dissipation analysis (Geodynamic explanation)

**MODEL PARAMETERS**	
crustal density (kg/m^3)	2800
sediment density (kg/m^3)	2000
acc. due to gravity, g (m/s^2)	9.81
crustal thickness (m)	10000
effective elastic thickness (m)	5000
Youngs modulus (Pa)	5E+10
poissons ratio	0.25
friction coefficient	0.4
F_f *	0.33
F_h *	0.66
Extensional velocity V (m/yr)†	0.0015
* partitioning of footwall uplift (F_f) vs hanging wall subsidence (F_h); † 2V = full extension rate; Buiter et al. (2008)	

5. References

Ackermann, F. Airborne laser scanning - present status and future expectations. *ISPRS Journal of Photogrammetry & Remote Sensing,* 54, 64-67 (1999).

Bubeck, A., Wilkinson, M., Roberts, G. P., Cowie, P. A., McCaffrey, K. J. W., Phillips, R. & Sammonds, P. The tectonic geomorphology of bedrock scarps on active normal faults in the Italian Apennines mapped using combined ground penetrating radar and terrestrial laser scanning. *Geomorphology*, 237, doi: 10.1016/j.geomorph.2014.03.011 (2015).

Buiter, S.J.H., Huismans**,** R.S., and Beaumont, C. Dissipation Analysis as a Guide to Mode Selection during Crustal Extension and Implications for the Styles of Sedimentary Basins, J. Geophys. Res., 113, B06406, doi:10.1029/2007JB005272 (2008).

Cowie, P. A., G. P. Roberts, J. Bull, & Visini, F. Relationships between fault geometry, slip rate variability and earthquake recurrence in extensional settings, *Geophys. J. Int.*, 189, 143–160, doi: 10.1111/j.1365-246X.2012.05378.x (2012).

Flood, M. & Gutelius, B. Commercial implications of topographic terrain mapping using scanning airborne laser radar. *Photogrammetric Eng. & Remote Sensing Journal*, 63, 327-329 (1997).

Galli, P. & Naso, J. Unmasking the 1349 earthquake source (southern Italy): paleoseismological and archaeoseismological indications from the Aquae Iuliae fault. *J. Struct. Geol*. 31, 128–149. http://dx.doi.org/10.1016/j.jsg.2008.09.007 (2009).

Galli, P., Messina, P., Giaccio, B., Peronace, E. & Quadrio, B. Early Pleistocene to late Holocene activity of the Magnola fault (Fucino fault system, central Italy), *Bollettino di Geofisica Teorica ed Applicata*, 53, 435-458 (2012).

Giraudi, C. & Frezzotti, M. Late Pleistocene glacial events in the central Apennines, Italy. *Quaternary Research*, 48, 280–290 (1997).

Gosse, J.C. & Phillips, F.M. Terrestrial in situ cosmogenic nuclides: theory and application. *Quaternary Science Reviews*, 20, 1475-1560. doi:10.1016/S0277-3791(00)00171-2 (2001).

Green, P. J. Reversible jump Markov chain Monte Carlo computation and Bayesian model determination. *Biometrika* 82(4): 711-732 (1995).

Guerrieri, L, Pascarella, F., Silvestri, S. & Serva, L. Evoluzione recente del paesaggio e dissesto geologico-idraulico: primi risultati in un'area campione dell'Appennino Centrale (valle del Salto – Rieti), *Mem. Soc. Geol. It.*, 57, 453-461 (2002).

Hastings, W. K. Monte Carlo sampling methods using Markov chains and their applications. *Biometrika* 57(1): 97-109 (1970).

Jol, M. & Bristow, C. GPR in sediments: advice on data collection, basic processing and interpretation, a good practice guide, in Ground Penetrating Radar in Sediments, *Geological Society, London, Special Publications*, eds Bristow C. S., Jol H. M. 211, pp 9–27 (2003).

Metropolis, N., et al. Equation of state calculations by fast computing machines. *The journal of Chemical Physics* 21(6): 1087-1092 (1953).

Michetti, A. M., Brunamonte, F., Serva, L. & Vittori, E. Trench investigations of the 1915 Fucino earthquake fault scarps (Abruzzo, central Italy): Geological evidence of large historical events, *J. Geophys. Res.,* 101, 5921–5936 , 10.1029/95JB02852 (1996).

Neal, A. Ground-penetrating radar and its use in sedimentology: principles, problems and progress, *Earth-Science Reviews*, 66, Issues 3–4, 261–330, doi:10.1016/j.earscirev.2004.01.004 (2004).

Roberts, G. P. & Michetti, A. M. Spatial and temporal variations in growth rates along active normal fault systems: an example from The Lazio–Abruzzo Apennines, central Italy. *J. Struct. Geol.*, 26, 339-376 (2004).

Sambridge, M., et al. (2006). Trans-dimensional inverse problems, model comparison and the evidence. *Geophys. J. Int.* 167(2): 528-542 (2006).

Schimmelpfennig, I., Benedetti, L., Finkel, R., Pik, R., Blard, P-H., Bourles, D., Burnard, P. & Williams, A. Sources of in-situ 36Cl in basaltic rocks. Implications for calibration of production rates. *Quaternary Geochronology,* 4, 441-461 doi:10.1016/j.quageo.2009.06.003 (2009).

Schlagenhauf, A., Gaudemer, Y., Benedetti, L., Manighetti, I., Palumbo, L., Schimmelpfennig, I., Finkel, R. & Pou, K. Using in situ Chlorine-36 cosmonuclide to recover past earthquake histories on limestone normal fault scarps: a reappraisal of methodology and interpretations, *Geophys. J. Int.*, 182, 36-72, doi: 10.1111/j.1365-246X.2010.04622.x (2010).

Schrott, L. & Sass, O. Application of field geophysics in geomorphology: Advances and limitations exemplified by case studies, *Geomorphology*, 93 (1-2), 55-73(2008).

Tucker, G. E., McCoy, S. W., Whittaker, A., Roberts, G. P., Lancaster, S. & Phillips, R. Geomorphic significance of postglacial bedrock scarps on normal-fault footwalls. *J. Geophys. Res*., 116, F01022, doi:10.1029/2010JF001861 (2011).

Vincent, K.R. & Chadwick, O.A. Synthesizing bulk-density for soils with abundance rock fragments, *Soil Science Society of America*, 58(2), 455-464, doi:10.2136/sssaj1994.03615995005800020030x (1994).

Wehr, A. & Lohr, U. Airborne laser scanning - an introduction and overview, *ISPRS Journal of Photogrammetry and Remote Sensing*, 54, 68-82 (1999).

Wells, D. L. & Coppersmith, K. J. New empirical relationships among magnitude, rupture length, rupture width, rupture area, and surface displacement. *Bull. Seism. Soc. Amer.*, 84, 974–1002 (1994).

Wilcken, K.M., Freeman, S.P.H.T., Schnabel, C., Binnie, S.A., Xu, S. & Phillips, R.J. ^{36}Cl accelerator mass spectrometry with a bespoke instrument, *Nuclear Instruments and Methods in Physics Research B*, 294, 107-114. doi:10.1016/j.nimb.2012.04.027 (2013).

6. Data Tables

Table 6.1.0 Mean chemical composition for each site including the upper slope sample at site FIAM. Tables 6.1.1-6.1.8 Cl content and chlorine isotopic composition for each sample. Table 6.1.9 Cl content and chlorine isotopic composition of processed blanks. For site MA3 see Schlagenhauf et al., 2010). Tables 6.2.x Input files for each fault scarp site for use in Schlagenhauf et al.'s 2010 Matlab® code (datarock.xls, datacolluvium.xls and datmagfield.xls) available online at https://www.dropbox.com/home/NatureScienceReports/data%20files%20as%20PDFs?preview=PDFs+of+all+data+txt+files.zip

Mean chemical composition for each site Sample measurements on homogeneous whole rock by fusion ICP, except for B which was determined by PGNAA

FAULT	Ca ICP	Al2O3 (Al)	CaO (Ca)	Fe2O3 (Fe)	K2O(K)	MnO (Mn)	MgO (Mg)	Na2O(Na)	P2O5(P)	SiO2 (Si)	TiO2(Ti)	B	U	V
	**[ppm]**	[wt.%]	[wt.%]	[wt.%]	[wt.%]	[wt.%]	[wt.%]	[wt.%]	[wt.%]	[wt.%]	[wt.%]	[ppm]	[ppm]	[ppm]
**FIAM**	391214	0.14	54.74	0.06	0.024	0.007	0.55	0.02	0.05	0.37	0.004	4.01	0.68	6.00
**GDM**	395426	0.09	55.33	0.05	0.010	0.003	0.39	0.03	0.03	0.41	0.002	2.90	0.39	0.00
**TREM**	399820	0.11	55.68	0.01	0.005	0.002	0.47	0.02	0.04	0.34	0.003	3.77	0.44	0.00
**PESC**	391572	0.08	54.79	0.05	0.010	0.004	0.43	0.03	0.02	0.22	0.002	4.26	0.07	0.00
**SSEB**	394542	0.03	55.37	0.00	0.000	0.004	0.39	0.00	0.03	0.00	0.000	2.00	0.31	0.00
**PARA**	402837	0.11	54.55	0.01	0.021	0.000	0.39	0.02	0.04	0.34	0.003	3.77	0.44	0.00
**FRAT**	396350	0.01	55.46	0.76		0.008	0.51	0.05					4.49	
**FIAM Upper Slope**	388322	0.08	54.06	0.08	0.005	0.002	0.41	0.02	0.01	0.61	0.002	1.80	0.21	0.00

FAULT	Li	As	Ba	Be	Bi	Cd	Ce	Co	Cr	Cs	Cu	Dy	W	Y
	[ppm]	[ppm]	[ppm]	[ppm]	[ppm]	[ppm]	[ppm]	[ppm]	[ppm]	[ppm]	[ppm]	[ppm]	[ppm]	[ppm]
**FIAM**	0.00	0.00	10.00	0.00	0.00	5.00	0.15	0.00	0.00	0.00	70.91	0.02	0.00	0.00
**GDM**	0.00	0.00	8.50	0.00	0.00	0.00	0.00	0.00	0.00	0.00	62.50	0.00	0.00	0.00
**TREM**	0.00	0.00	8.95	0.00	0.00	0.00	0.15	0.00	0.00	0.00	74.09	0.02	0.00	0.00
**PESC**	0.00	0.00	7.33	0.00	0.00	0.00	0.00	0.00	0.00	0.00	88.33	0.00	0.00	0.00
**SSEB**	0.92	0.00	3.08	0.00	0.00	0.27	0.53	2.12	0.00	0.00	0.00	0.12	0.00	1.37
**PARA**	0.00	0.00	8.95	0.00	0.00	0.00	0.15	0.00	0.00	0.00	74.09	0.02	0.00	0.00
**FRAT**							2.08					1.00		
**FIAM Upper Slope**	1.00	0.00	9.00	1.00	0.00	0.00	0.00	1.00	20.00	0.10	0.00	0.00	0.00	0.00

FAULT	Er	Eu	Ga	Gd	Ge	Hf	Ho	In	La	Lu	Mo	Nb	Yb	Zn
	[ppm]	[ppm]	[ppm]	[ppm]	[ppm]	[ppm]	[ppm]	[ppm]	[ppm]	[ppm]	[ppm]	[ppm]	[ppm]	[ppm]
**FIAM**	0.01	0.00	0.00	0.03	0.00	0.00	0.00	0.00	0.00	0.01	0.00	0.00	0.02	0.00
**GDM**	0.00	0.00	0.00	0.00	0.00	0.00	0.00	0.00	0.00	0.00	0.00	0.00	0.00	0.00
**TREM**	0.01	0.00	0.00	0.03	0.00	0.00	0.00	0.00	0.00	0.00	0.00	0.00	0.02	0.00
**PESC**	0.00	0.00	0.00	0.00	0.00	0.00	0.00	0.00	0.00	0.00	0.00	0.00	0.00	0.00
**SSEB**	0.09	0.02	0.00	0.11	0.00	0.00	0.03	0.00	0.65	0.01	0.00	0.00	0.08	0.00
**PARA**	0.01	0.00	0.00	0.03	0.00	0.00	0.00	0.00	0.00	0.00	0.00	0.00	0.02	0.00
**FRAT**	0.63	0.23		1.01			0.22		4.49	0.08			0.54	
**FIAM Upper Slope**	0.00	0.00	0.00	0.01	0.00	0.00	0.00	0.00	0.00	0.00	2.00	0.00	0.00	0.00

FAULT	Nd	Ni	Pb	Pr	Rb	Sb	Sm	Sn	Sr	Ta	Tb	Th	Tm	Zr
	[ppm]	[ppm]	[ppm]	[ppm]	[ppm]	[ppm]	[ppm]	[ppm]	[ppm]	[ppm]	[ppm]	[ppm]	[ppm]	[ppm]
**FIAM**	0.13	66.36	7.00	0.02	1.00	0.00	0.03	0.00	177.27	0.00	0.00	0.12	0.00	1.56
**GDM**	0.00	57.50	0.00	0.00	0.00	0.50	0.00	0.00	99.00	0.00	0.00	0.00	0.00	0.00
**TREM**	0.12	68.64	0.00	0.02	0.00	0.00	0.03	0.00	154.95	0.00	0.00	0.11	0.00	1.45
**PESC**	0.00	81.67	0.00	0.00	0.00	0.00	0.00	0.00	159.50	0.00	0.00	0.08	0.00	1.00
**SSEB**	0.47	21.52	5.35	0.11	0.39	0.00	0.10	0.00	116.05	0.00	0.02	0.04	0.01	0.00
**PARA**	0.12	68.64	0.00	0.02	0.00	0.00	0.03	0.00	154.95	0.00	0.00	0.11	0.00	1.45
**FRAT**	0.00		0.12	1.11			0.96				0.16	0.03	0.09	
**FIAM Upper Slope**	0.00	60.00	0.00	0.00	1.00	0.00	0.01	0.00	106.00	0.00	0.00	0.05	0.00	1.00

Table 6.1.1: Cl content and chlorine isotopic composition of limestone fault samples

Fault: **Fiamignano**

LOCATION 0344586E/4681827N 33T

ELEVATION 1148m

SHIELDING 0.933169931

**AMS ID**	**Z, position on scarp**	**m sample**	**m_cl spike**[a]	**36Cl/Cl**[b]	**± 1 sigma uncertainty**			**37Cl/35Cl**	**Clnat AMS**	**N_Cl36,rock**	
SUERC-	[cm]	[g]	[mg]	derived from 36Cl/35Cl		derived from 36Cl/37Cl			[ppm]	36Cl [atoms/g]	s36Cl [atoms/g]
c1523	-104	29.8377	1.3233	6.017E-14	2.389E-15	3.012E-13	1.222E-14	0.0573	1.94	54430	3208
c1524	-95	29.7949	1.3160	6.192E-14	1.831E-15	3.169E-13	9.371E-15	0.0561	1.63	55808	2878
c1525	-85	29.9595	1.3041	6.771E-14	2.813E-15	2.863E-13	1.189E-14	0.0677	4.60	64473	3656
c1526	-76	30.5655	2.6321	6.721E-14	3.367E-15	3.038E-13	1.541E-14	0.0634	6.84	66118	4156
c1531	-66	30.3639	1.3174	6.332E-14	2.954E-15	2.900E-13	1.353E-14	0.0626	3.24	57930	3607
c1532	-57	30.0304	1.3127	6.655E-14	2.127E-15	3.393E-13	1.085E-14	0.0561	1.62	59506	3085
c1533	-47	29.4730	1.3114	6.514E-14	1.914E-15	3.220E-13	9.463E-15	0.0578	2.07	59802	3008
c1534	-38	29.8354	1.2975	7.455E-14	2.405E-15	3.870E-13	1.248E-14	0.0551	1.36	66910	3381
c1539	-28	29.9620	1.3074	7.536E-14	1.963E-15	3.823E-13	9.961E-15	0.0561	1.61	67569	3131
c1540	-19	30.4197	1.3094	7.083E-14	2.641E-15	3.688E-13	1.375E-14	0.0547	1.25	62031	3399
c1541	-10	30.5017	1.3021	7.137E-14	3.728E-15	3.758E-13	1.946E-14	0.0541	1.10	62152	4115
c1325	0	31.8499	0.7587	7.585E-14	2.234E-15	1.302E-13	3.835E-15	0.1708	0.58	65660	3522
c1542	135	30.1538	1.3008	7.368E-14	2.183E-15	3.796E-13	1.125E-14	0.0554	1.42	65452	3204
c1324	155	30.2402	0.7619	7.054E-14	1.858E-15	1.095E-13	2.884E-15	0.1888	11.53	76803	6287
c1548	175	30.0438	1.3001	7.134E-14	3.365E-15	3.230E-13	1.523E-14	0.0630	3.33	66064	4038
c1323	237	29.7945	0.7587	8.071E-14	2.301E-15	1.361E-13	3.882E-15	0.1738	2.22	77548	3912
c1549	257	29.7956	1.3114	8.528E-14	2.852E-15	3.825E-13	1.279E-14	0.0636	3.55	80244	3900
c1316	277	28.5079	0.7656	7.558E-14	5.058E-15	1.295E-13	8.663E-15	0.1704	0.43	72598	6406
c1679	297	30.5461	0.9840	1.154E-13	3.374E-15	5.805E-13	1.698E-14	0.0574	1.82	77454	2976
c1680	337	31.5523	0.9698	1.198E-13	3.627E-15	6.237E-13	1.889E-14	0.0555	1.40	77190	3039
c1681	357	30.3490	0.9704	1.057E-13	2.624E-15	5.465E-13	1.356E-14	0.0559	1.53	70665	2486
c1682	377	30.9903	0.9760	1.322E-13	5.803E-15	6.696E-13	2.939E-14	0.0572	1.74	87867	4480
c1314	397	31.9382	0.7631	8.648E-14	4.588E-15	1.483E-13	7.871E-15	0.1705	0.43	75653	5514
c1683	417	30.7874	0.9803	1.278E-13	3.784E-15	6.585E-13	1.950E-14	0.0561	1.56	84883	3284
c1307	437	28.4690	0.7600	8.348E-14	2.471E-15	1.410E-13	4.171E-15	0.1735	2.16	84033	4243
c1690	453	30.3712	0.9723	1.215E-13	3.500E-15	5.739E-13	1.654E-14	0.0611	2.49	83570	3154
c1684	457	31.1623	1.0131	1.309E-13	3.600E-15	6.543E-13	1.800E-14	0.0578	1.91	86804	3197

c1305	473	29.2372	0.7625	9.006E-14	1.864E-15	1.484E-13	3.072E-15	0.1778	4.60	92955	4041
c1306	477	27.6072	0.7656	8.966E-14	2.491E-15	1.515E-13	7.078E-15	0.1742	2.66	94790	12338
c1691	479	30.5935	0.9902	1.259E-13	3.863E-15	6.520E-13	2.000E-14	0.0558	1.53	84020	3327
c1689	497	30.1326	0.9618	1.238E-13	3.645E-15	6.271E-13	1.846E-14	0.0571	1.74	84292	3247
c1693	525	31.8932	0.9797	1.176E-13	3.352E-15	4.107E-13	1.171E-14	0.0827	6.72	85962	3112
c1694	545	30.1474	0.9698	1.180E-13	3.408E-15	4.909E-13	1.418E-14	0.0694	4.14	85104	3171
c1297	565	29.6581	0.7637	9.595E-14	2.524E-15	1.634E-13	4.299E-15	0.1701	0.26	90687	4620
c1692	580	30.6623	0.9865	1.326E-13	4.017E-15	7.219E-13	2.186E-14	0.0531	1.04	87271	3442
c1699	585	30.0143	0.9556	1.314E-13	3.227E-15	7.014E-13	1.723E-14	0.0542	1.22	88702	3096
c1700	625	30.8521	0.9958	1.436E-13	4.301E-15	7.514E-13	2.250E-14	0.0552	1.42	95098	3700
c1280	645	29.1458	0.9315	1.0014E-13	3.189E-15	1.9407E-13	6.307E-15	0.1504	4.50	108978	4020
c1709	664	30.2474	1.0416	1.401E-13	3.932E-15	6.634E-13	1.862E-14	0.0610	2.37	105181	3799
c1279	720	29.2775	0.9365	1.0615E-13	3.228E-15	2.1408E-13	6.657E-15	0.1444	1.60	110273	3906
c1710	740	29.8467	1.0570	1.360E-13	3.885E-15	6.735E-13	1.924E-14	0.0584	1.90	102240	3758
c1711	750	30.1703	1.0286	1.345E-13	4.040E-15	6.785E-13	2.038E-14	0.0574	1.63	99444	3786
c1274	760	30.3658	0.9315	1.1239E-13	3.526E-15	2.1333E-13	6.835E-15	0.1535	5.86	121450	4346
c1934	765	31.2917	1.0787	1.533E-13	4.452E-15	7.251E-13	2.105E-14	0.0601	2.40	109013	3388
c1273	770	29.6636	0.9378	1.0299E-13	2.578E-15	1.9389E-13	5.014E-15	0.1547	6.67	114315	3400
c1935	775	30.2243	1.0539	1.440E-13	3.728E-15	7.276E-13	1.884E-14	0.0563	1.67	103757	2911
c1712	790	29.8705	1.0465	1.480E-13	4.076E-15	7.656E-13	2.109E-14	0.0558	1.36	109858	3955
c1272	810	29.3746	0.9378	1.0695E-13	3.369E-15	2.1009E-13	6.758E-15	0.1483	3.46	114240	4150
c1713	830	32.0618	1.0341	1.475E-13	4.385E-15	7.765E-13	2.308E-14	0.0549	1.09	101581	3845
c1937	840	31.1975	1.0539	1.498E-13	4.796E-15	7.953E-13	2.547E-14	0.0535	1.09	103221	3524
c1271	850	30.1259	0.9403	1.1532E-13	3.62E-15	2.2849E-13	7.324E-15	0.1471	2.81	119755	4304
c1714	870	30.9758	1.0286	1.516E-13	4.022E-15	7.787E-13	2.066E-14	0.0563	1.38	108875	3825
c1942	880	30.7135	1.0508	1.596E-13	4.025E-15	8.136E-13	2.051E-14	0.0558	1.54	113250	3088
c1270	890	29.2204	0.9340	1.0874E-13	3.455E-15	2.0027E-13	6.496E-15	0.1582	8.67	126634	4579
c1719	910	31.4269	1.0397	1.502E-13	4.298E-15	7.788E-13	2.229E-14	0.0558	1.29	106097	3910
c1943	925	30.6188	1.0304	1.645E-13	4.741E-15	8.577E-13	2.472E-14	0.0545	1.27	116327	3583
c1269	930	30.9894	0.9378	1.1597E-13	3.707E-15	2.2636E-13	7.383E-15	0.1493	3.74	119127	4338
c1720	950	29.9881	1.0416	1.430E-13	4.120E-15	7.416E-13	2.137E-14	0.0557	1.33	105605	3917
c1944	965	30.9134	1.0453	1.684E-13	3.544E-15	8.734E-13	1.838E-14	0.0548	1.34	118283	2739
c1264	970	30.7612	0.9340	1.2332E-13	3.66E-15	2.442E-13	7.419E-15	0.1471	2.73	125961	4277
c1721	990	29.9789	1.0484	1.550E-13	4.571E-15	8.307E-13	2.449E-14	0.0539	0.99	113787	4284
c1945	1000	30.7605	1.0477	1.691E-13	4.918E-15	8.655E-13	2.517E-14	0.0555	1.48	119740	3715
c1252	1010	29.2370	0.9215	1.2427E-13	3.972E-15	2.4817E-13	8.095E-15	0.1459	6.95	129714	6218

c1722	1030	31.4305	1.0242	1.641E-13	4.453E-15	8.186E-13	2.221E-14	0.0579	1.66	117125	4146
c1946	1040	30.5752	1.0446	1.610E-13	4.648E-15	8.189E-13	2.364E-14	0.0559	1.56	114788	3543
c1251	1050	28.3052	0.9215	1.1667E-13	3.547E-15	2.2691E-13	7.054E-15	0.1498	9.11	129147	5994
c1723	1070	31.3451	1.0360	1.771E-13	4.948E-15	9.247E-13	2.584E-14	0.0553	1.19	125433	4534
c1947	1080	30.2883	1.0496	1.782E-13	5.113E-15	9.381E-13	2.692E-14	0.0540	1.22	127509	3901
c1250	1090	28.9485	0.9290	1.2295E-13	3.554E-15	2.5284E-13	7.491E-15	0.1416	5.09	125426	5829
c1724	1110	29.8299	1.0385	1.726E-13	4.563E-15	9.450E-13	2.498E-14	0.0528	0.77	126962	4465
c1952	1120	30.9125	1.0366	1.822E-13	5.209E-15	9.685E-13	2.769E-14	0.0535	1.08	127488	3884
c1811	1127	31.6428	1.0471	1.858E-13	5.410E-15	9.875E-13	2.878E-14	0.0543	1.19	122678	8087
c1249	1130	29.8828	0.9196	1.4066E-13	4.362E-15	2.7907E-13	8.843E-15	0.1469	7.24	145796	6799
c1242	1147	29.9473	0.9278	1.3142E-13	3.242E-15	2.6681E-13	6.807E-15	0.1435	5.75	132043	5678
c1728	1150	31.3502	1.0348	1.758E-13	5.155E-15	9.308E-13	2.730E-14	0.0546	1.06	124142	4634
c1955	1157	30.4426	1.0378	1.905E-13	5.474E-15	9.433E-13	2.711E-14	0.0574	1.84	137938	4213
c1812	1167	30.1015	1.0156	1.810E-13	5.004E-15	9.336E-13	2.584E-14	0.0559	1.52	126402	8201
c1243	1175	29.4432	0.9184	1.4062E-13	4.14E-15	2.8054E-13	8.458E-15	0.1460	6.92	146842	6701
c1953	1180	31.4178	1.0378	1.906E-13	5.516E-15	8.916E-13	2.580E-14	0.0608	2.43	135948	4177
c1241	1187	29.4569	0.9259	1.423E-13	4.501E-15	2.9804E-13	9.625E-15	0.1391	3.91	141285	6832
c1729	1195	30.0725	1.0106	1.783E-13	5.428E-15	9.031E-13	2.750E-14	0.0571	1.55	132719	5053
c2165	1197	30.1560	1.0947	1.683E-13	4.815E-15	8.103E-13	2.319E-14	0.0599	2.68	133211	3988
c1244	1215	29.4622	0.9202	1.3566E-13	4.013E-15	2.6946E-13	8.162E-15	0.1467	7.26	142112	6499
c1240	1227	29.2713	0.9278	1.3446E-13	3.909E-15	2.5982E-13	7.74E-15	0.1507	9.31	146326	6551
c1954	1230	30.8770	1.0440	1.865E-13	4.246E-15	9.655E-13	2.198E-14	0.0549	1.35	131577	3253
c1817	1247	30.5737	1.0001	2.239E-13	6.470E-15	1.078E-12	5.156E-14	0.0604	2.32	158190	12631
c1234	1267	30.4594	0.9209	1.5267E-13	4.333E-15	3.072E-13	8.964E-15	0.1448	6.18	153456	6883
c2170	1272	29.9478	1.0904	1.8429E-13	5.309E-15	1.0067E-12	2.9E-14	0.0528	1.19	142232	4278
c1824	1281	30.1192	0.9871	2.096E-13	5.302E-15	1.126E-12	2.850E-14	0.0537	1.07	144759	9347
c2173	1286	30.7314	1.0947	2.0766E-13	5.937E-15	1.1311E-12	3.234E-14	0.0529	1.18	156637	4661
c1818	1287	31.1922	1.0032	2.288E-13	5.753E-15	1.146E-12	5.215E-14	0.0579	1.81	156344	11126
c1231	1296	29.5179	0.9353	1.491E-13	4.359E-15	3.079E-13	9.223E-15	0.1410	4.76	150308	6933
c1233	1307	28.4846	0.9278	1.4007E-13	3.404E-15	2.753E-13	6.925E-15	0.1482	8.30	153957	6427
c1825	1316	31.4319	0.9816	2.285E-13	6.634E-15	1.203E-12	3.495E-14	0.0548	1.21	152068	10010
c2171	1317	30.8523	1.0972	2.045E-13	5.899E-15	1.0972E-12	3.165E-14	0.0537	1.34	154195	4629
c2174	1326	30.5547	1.0873	2.0558E-13	5.939E-15	1.0829E-12	3.129E-14	0.0548	1.56	157460	4732
c1819	1327	31.4491	0.9853	2.340E-13	6.707E-15	1.230E-12	3.527E-14	0.0550	1.25	155948	10230
c2172	1332	30.8417	1.0879	2.0713E-13	5.889E-15	1.1351E-12	3.227E-14	0.0526	1.11	155447	4601
c1230	1336	24.3182	0.9309	1.4395E-13	4.615E-15	2.968E-13	9.710E-15	0.1413	5.91	176175	8497

c2175	1341	30.0021	1.0923	2.0491E-13	4.611E-15	1.088E-12	2.448E-14	0.0543	1.50	159376	3773
c1232	1342	29.0640	0.9309	1.5829E-13	4.731E-15	3.2223E-13	9.857E-15	0.1431	5.76	165107	7606
c1826	1356	29.8784	0.9884	2.296E-13	5.822E-15	1.173E-12	2.976E-14	0.0566	1.62	162343	10407
c2180	1366	31.4010	1.0774	2.2129E-13	6.38E-15	1.1647E-12	3.358E-14	0.0548	1.51	165050	4941
c1259	1376	29.4734	0.9372	1.814E-13	6.115E-15	3.6107E-13	1.24E-14	0.1464	2.53	196857	7270
c2181	1381	30.4058	1.0818	2.0991E-13	6.045E-15	1.144E-12	3.295E-14	0.0529	1.18	160051	4794
c1832	1396	30.0672	1.0057	2.325E-13	6.777E-15	1.236E-12	3.605E-14	0.0543	1.20	161576	10659
c2040	1324	30.5738	1.1319	2.139E-13	5.333E-15	1.131E-12	2.820E-14	0.0554	1.40	184984	4943
c2069	1334	30.5853	1.0570	2.157E-13	4.403E-15	1.153E-12	2.354E-14	0.0536	0.93	148973	3433
c2041	1344	30.0860	1.1220	2.045E-13	4.568E-15	1.121E-12	2.504E-14	0.0534	1.01	157056	3696
c2070	1354	30.4011	1.0267	2.304E-13	5.234E-15	1.263E-12	2.868E-14	0.0524	0.69	159463	4009
c2046	1364	29.9480	1.1281	2.078E-13	6.042E-15	1.155E-12	3.357E-14	0.0526	0.86	159624	4838
c2071	1374	30.2985	1.0397	2.252E-13	5.133E-15	1.237E-12	2.819E-14	0.0523	0.68	156267	3946
c2047	1384	31.0057	1.1238	2.179E-13	5.425E-15	1.193E-12	2.968E-14	0.0534	0.98	162504	4226
c2076	1394	30.4778	1.0323	2.528E-13	6.751E-15	1.353E-12	3.613E-14	0.0537	0.93	176091	5077
c2048	1404	31.0010	1.1257	2.277E-13	5.769E-15	1.247E-12	3.342E-14	0.0531	0.92	169303	4592
c2077	1414	31.1216	1.0249	2.412E-13	6.911E-15	1.313E-12	3.762E-14	0.0527	0.72	163373	5032
c2049	1424	29.7574	1.1201	2.322E-13	6.587E-15	1.310E-12	3.717E-14	0.0518	0.69	179272	5278
c2078	1434	30.2739	1.0434	2.429E-13	6.959E-15	1.385E-12	3.967E-14	0.0504	0.33	167499	5163
c2050	1444	29.9802	1.1251	2.304E-13	6.626E-15	1.289E-12	3.707E-14	0.0522	0.77	176812	5277
c2079	1454	30.4334	1.0515	2.554E-13	5.697E-15	1.390E-12	3.101E-14	0.0528	0.78	177467	4370
c2051	1464	30.1046	1.1275	2.430E-13	6.935E-15	1.317E-12	3.760E-14	0.0539	1.11	187573	5541
c2080	1474	31.4985	1.0372	2.686E-13	7.712E-15	1.401E-12	4.024E-14	0.0550	1.13	182295	5596
c2056	1484	44.7077	1.1084	3.440E-13	6.971E-15	1.785E-12	3.617E-14	0.0555	0.95	179992	3758
c2081	1494	44.7353	1.0174	3.745E-13	1.064E-14	1.866E-12	5.301E-14	0.0576	1.11	182173	5481
c2057	1504	44.8835	1.1152	3.485E-13	8.581E-15	1.746E-12	4.299E-14	0.0575	1.23	183497	4631
c2082	1514	44.1501	1.0378	2.161E-13	4.836E-15	3.573E-13	7.994E-15	0.1738	31.46	217397	5374
c2058	1564	30.1868	1.1009	2.708E-13	6.731E-15	1.500E-12	3.728E-14	0.0520	0.71	205416	5283
c2087	1574	29.7066	1.0267	2.743E-13	6.84E-15	1.519E-12	3.788E-14	0.0521	0.65	195221	5283
c2059	1584	31.4272	1.1053	2.832E-13	8.161E-15	1.551E-12	4.470E-14	0.0526	0.80	207198	6147
c2088	1594	30.9586	1.0502	2.850E-13	8.192E-15	1.538E-12	4.42E-14	0.0534	0.87	196004	6015
c2060	1604	31.5924	1.0991	2.790E-13	8.072E-15	1.528E-12	4.421E-14	0.0526	0.79	202998	6048
c2061	1971	31.5828	1.0985	3.226E-13	9.222E-15	8.442E-13	2.413E-14	0.1101	14.93	314903	9196
c2089	1991	30.7130	1.0205	3.662E-13	8.215E-15	1.152E-12	2.585E-14	0.0916	9.03	307573	7365
c2090	2011	30.1868	1.0131	4.115E-13	1.184E-14	1.877E-12	5.399E-14	0.0632	2.72	305622	9262
c1260	2148	29.5336	0.9334	3.3217E-13	1.047E-14	6.4299E-13	2.07E-14	0.1505	4.50	378783	12795

c2182	2158	31.1225	1.0737	4.3215E-13	1.061E-14	1.6773E-12	4.119E-14	0.0743	5.72	360474	9149
c1833	2168	31.2450	1.0187	4.704E-13	1.049E-14	1.956E-12	4.367E-14	0.0694	4.12	340382	20063
c2183	2178	31.1856	1.0762	4.3102E-13	1.217E-14	1.6725E-12	4.723E-14	0.0743	5.72	358867	10416
c1261	2188	30.5642	0.9321	3.5732E-13	1.073E-14	6.9597E-13	2.138E-14	0.1496	3.91	391767	13142
c2184	2198	30.3481	1.0768	4.3967E-13	1.233E-14	1.7727E-12	4.973E-14	0.0715	5.21	371103	10694
c1834	2208	30.0871	0.9853	4.933E-13	1.242E-14	2.052E-12	5.170E-14	0.0694	4.14	369368	22257
c2189	2218	29.9314	1.0675	4.3703E-13	1.085E-14	1.7417E-12	4.326E-14	0.0724	5.45	375383	9624
c1262	2228	30.5054	0.9328	3.5958E-13	1.084E-14	6.8346E-13	2.108E-14	0.1532	5.69	405822	13119
c2190	2238	29.9908	1.0657	4.6665E-13	1.13E-14	2.1941E-12	5.311E-14	0.0614	2.94	379827	9487
c1839	2248	30.3311	1.0044	5.102E-13	1.458E-14	2.285E-12	6.537E-14	0.0645	3.17	370683	23314
c1263	2258	29.7629	0.9328	3.8106E-13	1.152E-14	7.3359E-13	2.268E-14	0.1513	4.86	435102	14090

[a]Mass of $^{35/37}$Cl spike added to sample prior to dissolution. Spike concentration: for samples c1230 - c1325 the mgCl/g solution = 6.2602, ^{37}at/^{35}at = 0.0551; for samples c1523 - c1549 the mgCl/g solution = 6.6199, ^{37}at/^{35}at = 0.0507; for samples c1679 - c2190 the mgCl/g solution = 6.1850, ^{37}at/^{35}at = 0.0510.

[b]36Cl/Cl is based on either the measured ^{36}Cl/^{35}Cl or ^{36}Cl/^{37}Cl ratios assuming natural ^{35}Cl/^{37}Cl ratios.

Table 6.1.2: Cl content and chlorine isotopic composition of limestone fault samples

Fault: **Gio dei Marsi**

LOCATION 0393515E/464452N 33T

ELEVATION 1023m

SHIELDING 0.875020669

**AMS ID**	**Z, position on scarp**	**m sample**	**m_cl spike**[a]	**36Cl/Cl**[b]	**± 1 sigma uncertainty**			**37Cl/35Cl**	**Clnat AMS**	**N_Cl36,rock**	
SUERC-	[cm]	[g]	[mg]	derived from 36Cl/35Cl		derived from 36Cl/37Cl			[ppm]	36Cl [atoms/g]	s36Cl [atoms/g]
c2346	-183	30.3291	1.2252	7.2755E-14	2.047E-15	2.7803E-13	7.854E-15	0.0763	6.69	68130	2073
c2347	-174	30.0026	1.2661	6.8423E-14	2.033E-15	2.7085E-13	8.076E-15	0.0736	6.24	63953	2064
c2348	-164	30.8250	1.2640	7.4033E-14	2.163E-15	2.9445E-13	8.636E-15	0.0733	5.99	67539	2128
c2349	-154	30.4646	1.1843	7.763E-14	2.369E-15	3.0774E-13	9.424E-15	0.0736	5.75	71351	2335
c2467	-145	30.8382	1.2331	6.571E-14	2.290E-15	2.525E-13	8.801E-15	0.0750	6.34	57558	2482
c2468	-135	30.7810	1.2690	7.476E-14	2.514E-15	2.977E-13	1.001E-14	0.0725	5.83	65975	2673
c2469	-125	30.6268	1.2446	7.100E-14	2.313E-15	2.707E-13	8.821E-15	0.0756	6.61	63472	2532
c2470	-116	30.1387	1.2252	7.125E-14	2.190E-15	2.759E-13	8.481E-15	0.0745	6.30	64241	2446
c2471	-106	30.4018	1.2209	7.313E-14	2.044E-15	2.689E-13	7.518E-15	0.0787	7.40	67143	2340
c2472	-97	30.4560	1.2274	7.944E-14	2.471E-15	2.875E-13	8.944E-15	0.0796	7.69	73657	2737
c2479	-87	30.9481	1.3372	7.881E-14	2.192E-15	2.885E-13	8.025E-15	0.0791	8.36	77937	2385
c2480	-77	29.9434	1.3207	7.216E-14	2.063E-15	2.680E-13	7.663E-15	0.0779	8.15	72665	2302
c2481	-68	31.1138	1.3566	7.953E-14	2.283E-15	3.090E-13	8.870E-15	0.0745	7.03	76489	2412
c2482	-58	30.7508	1.3056	8.120E-14	1.996E-15	3.103E-13	7.627E-15	0.0756	7.17	79048	2154
c1746	-49	31.8204	1.0279	1.202E-13	3.499E-15	5.021E-13	1.461E-14	0.0693	3.80	73620	2333
c2487	-48	30.1136	1.3221	7.497E-14	2.149E-15	2.870E-13	8.226E-15	0.0748	7.17	86963	7140
c1747	-39	30.3634	1.0026	1.113E-13	3.300E-15	4.759E-13	1.411E-14	0.0674	3.49	83368	2529
c2488	-38	30.0180	1.3042	8.361E-14	2.313E-15	3.155E-13	8.727E-15	0.0760	7.45	83066	6909
c1748	-29	30.5976	0.9797	1.139E-13	3.481E-15	4.887E-13	1.494E-14	0.0675	3.40	88390	3698
c2489	-28	30.8220	1.3307	8.961E-14	3.493E-15	3.295E-13	1.285E-14	0.0778	7.95	84443	7061
c1749	-20	30.4360	0.9958	1.198E-13	3.573E-15	5.181E-13	1.545E-14	0.0669	3.35	81142	3433
c2490	-19	31.1703	1.3049	8.444E-14	3.387E-15	3.150E-13	1.209E-14	0.0765	7.33	89224	7446
c1750	-10	31.1346	0.9605	1.242E-13	3.609E-15	5.185E-13	1.506E-14	0.0693	3.63	83405	2498
c2491	-9	30.2361	1.3071	8.521E-14	2.328E-15	3.320E-13	9.068E-15	0.0736	6.70	91209	7527
c1751	0	31.1908	0.9748	1.337E-13	3.980E-15	5.675E-13	1.690E-14	0.0682	3.46	88366	2889
c2492	0	31.2068	1.2884	9.280E-14	2.801E-15	3.590E-13	1.084E-14	0.0740	6.52	97798	8125
c1756	10	30.9722	0.9778	1.189E-13	5.072E-15	5.145E-13	2.195E-14	0.0662	3.10	86145	7758

c1757	20	31.5945	0.9624	1.313E-13	3.446E-15	5.508E-13	1.446E-14	0.0690	3.52	95037	7758
c1758	29	31.2004	0.9661	1.234E-13	3.803E-15	5.072E-13	1.563E-14	0.0704	3.86	90959	7529
c2497	39	30.6726	1.2970	1.011E-13	2.590E-15	4.387E-13	1.123E-14	0.0663	4.53	94934	2647
c2498	49	30.2271	1.3013	1.0273E-13	2.993E-15	4.4687E-13	1.302E-14	0.0660	4.54	95535	3002
c1760	58	30.5207	0.9680	1.340E-13	4.038E-15	5.048E-13	1.521E-14	0.0768	5.29	97729	3075
c1913	68	30.4353	1.0651	1.138E-13	2.088E-15	3.994E-13	7.329E-15	0.0825	3.59	94991	2371
c1914	68	31.4779	1.0644	1.150E-13	2.292E-15	3.748E-13	7.472E-15	0.0890	5.08	96125	2792
c1759	87	31.4405	0.9519	1.423E-13	4.123E-15	5.625E-13	1.630E-14	0.0732	4.32	104261	8379
c2499	87	29.6016	1.3056	9.7419E-14	2.826E-15	4.0962E-13	1.188E-14	0.0683	5.29	105557	8573
c1915	106	31.4260	1.0539	1.174E-13	2.141E-15	3.481E-13	6.352E-15	0.0977	7.35	102830	2748
c1916	125	30.7058	1.0490	1.172E-13	5.019E-15	3.919E-13	1.833E-14	0.0868	4.57	99249	4891
c1917	145	30.2333	1.0471	1.182E-13	4.733E-15	3.471E-13	1.591E-14	0.0988	7.91	108265	5308
c1922	164	30.3607	1.0348	1.304E-13	3.918E-15	3.656E-13	1.099E-14	0.1020	8.71	120281	3931
c1923	183	30.7253	1.0279	1.373E-13	3.947E-15	4.558E-13	1.310E-14	0.0862	4.33	115438	3563
c1924	202	30.7894	1.0360	1.346E-13	3.876E-15	4.052E-13	1.167E-14	0.0950	6.64	118101	3682
c1761	217	30.7361	0.9686	1.697E-13	4.978E-15	6.678E-13	1.959E-14	0.0735	4.56	125632	10486
c1766	217	30.5835	0.9797	1.691E-13	4.931E-15	7.387E-13	2.154E-14	0.0663	3.16	129535	10507
c1925	236	30.4705	1.0378	1.535E-13	4.380E-15	5.029E-13	1.435E-14	0.0873	4.69	131428	4012
c1926	236	30.1222	1.0403	1.658E-13	4.145E-15	6.541E-13	1.636E-14	0.0725	1.20	133882	3576
c1927	255	30.0893	1.0558	1.634E-13	4.732E-15	6.265E-13	1.815E-14	0.0746	1.70	133441	4098
c2036	275	30.2401	1.0521	1.824E-13	5.515E-15	8.375E-13	2.560E-14	0.0638	2.73	135057	4313
c2037	294	30.1094	1.0273	1.756E-13	4.387E-15	6.654E-13	1.662E-14	0.0773	5.58	139200	3671
c2038	313	30.2130	1.0564	1.828E-13	4.568E-15	7.153E-13	1.788E-14	0.0748	5.14	143278	3773
c2500	328	30.9393	1.2970	1.6254E-13	4.778E-15	7.4854E-13	2.2E-14	0.0624	3.48	151234	4690
c2338	352	29.9400	1.3020	1.805E-13	5.305E-15	8.1127E-13	2.393E-14	0.0649	4.08	168147	5080
c2339	371	30.6582	1.2590	1.8069E-13	4.609E-15	7.998E-13	2.05E-14	0.0659	4.10	164683	4326
c2344	390	30.2466	1.2561	1.9563E-13	5.812E-15	8.5465E-13	2.548E-14	0.0667	4.34	181582	5531
c2345	409	30.4077	1.2654	2.0309E-13	5.979E-15	9.2459E-13	2.732E-14	0.0641	3.70	185571	5598
c1767	429	31.5101	0.9438	2.525E-13	7.315E-15	9.257E-13	2.682E-14	0.0789	5.43	193073	15335

[a]Mass of $^{35/37}$Cl spike added to sample prior to dissolution. Spike concentration: for samples c1746 - c2038 the mgCl/g solution = 6.1850, ^{37}at/^{35}at = 0.0510; for samples c2338 - c2500 the mgCl/g solution = 7.1777, ^{37}at/^{35}at = 0.0510.

[b]36Cl/Cl is based on either the measured ^{36}Cl/^{35}Cl or ^{36}Cl/^{37}Cl ratios assuming natural ^{35}Cl/^{37}Cl ratios.

Table 6.1.3: Cl content and chlorine isotopic composition of limestone fault samples

Fault: **Parasano**
LOCATION 0392328E/4650380N 33T
ELEVATION 1268m
SHIELDING 0.798624984

**AMS ID**	**Z, position on scarp**	**m sample**	**m_cl spike**[a]	**36Cl/Cl**[b]	**± 1 sigma uncertainty**			**37Cl/35Cl**	**Clnat AMS**	**N_Cl36,rock**	
SUERC-	[cm]	[g]	[mg]	derived from 36Cl/35Cl		derived from 36Cl/37Cl			[ppm]	36Cl [atoms/g]	s36Cl [atoms/g]
c2534	-173	30.0823	1.4386	1.131E-13	3.322E-15	3.117E-13	9.259E-15	0.1045	18.81	1.457E+05	4.55E+03
c2535	-146	30.6415	1.4247	1.204E-13	3.087E-15	3.362E-13	8.747E-15	0.1031	17.69	1.508E+05	4.14E+03
c2536	-106	30.1098	1.4131	1.296E-13	3.800E-15	3.708E-13	1.100E-14	0.1006	16.79	1.626E+05	5.05E+03
c2537	-71	30.4848	1.3838	1.535E-13	4.487E-15	4.315E-13	1.276E-14	0.1024	16.98	1.919E+05	5.92E+03
c2543	-36	30.8012	1.3838	1.725E-13	3.947E-15	4.769E-13	1.112E-14	0.1041	17.51	2.160E+05	5.30E+03
c2544	0	31.0518	1.4077	1.935E-13	5.135E-15	5.382E-13	1.464E-14	0.1035	17.41	2.412E+05	6.80E+03
c2545	35	30.1283	1.3977	2.242E-13	6.456E-15	6.688E-13	1.949E-14	0.0965	14.94	2.767E+05	8.36E+03
c2546	70	30.2410	1.4023	2.527E-13	6.432E-15	7.708E-13	1.992E-14	0.0943	14.07	3.075E+05	8.28E+03
c2547	106	30.5225	1.4000	2.703E-13	7.751E-15	7.028E-13	2.040E-14	0.1107	20.79	3.587E+05	1.07E+04
c2548	141	30.7832	1.4000	2.892E-13	8.289E-15	7.489E-13	2.172E-14	0.1112	20.84	3.821E+05	1.17E+04
c2553	176	31.5127	1.4085	3.253E-13	9.324E-15	8.194E-13	2.376E-14	0.1143	21.90	4.291E+05	1.28E+04
c2554	211	30.0947	1.3900	3.246E-13	7.317E-15	8.296E-13	1.905E-14	0.1126	21.82	4.417E+05	1.06E+04
c2555	264	31.7454	1.4046	3.323E-13	8.353E-15	7.700E-13	1.966E-14	0.1242	26.54	4.627E+05	1.22E+04
c2556	300	30.4478	1.3984	3.316E-13	9.622E-15	8.074E-13	2.370E-14	0.1182	24.42	4.626E+05	1.40E+04
c2557	335	30.0996	1.3946	3.703E-13	1.085E-14	9.860E-13	2.921E-14	0.1081	20.11	4.818E+05	1.61E+04
c2563	370	30.2613	1.4069	4.267E-13	1.220E-14	1.095E-12	3.168E-14	0.1121	22.03	5.683E+05	1.92E+04
c2564	419	30.8521	1.3560	4.402E-13	1.282E-14	1.027E-12	3.026E-14	0.1234	26.23	6.098E+05	2.00E+04

[a]Mass of $^{35/37}$Cl spike added to sample prior to dissolution. Spike concentration: mgCl/g solution = 7.1777, ^{37}at/^{35}at = 0.0510.

[b]36Cl/Cl is based on either the measured ^{36}Cl/^{35}Cl or ^{36}Cl/^{37}Cl ratios assuming natural ^{35}Cl/^{37}Cl ratios.

Table 6.1.4: Cl content and chlorine isotopic composition of limestone fault samples

Fault: **Tre Monti**

LOCATION 037305E/4658263N 33T

ELEVATION 1009m

SHIELDING 0.813219452

**AMS ID**	**Z, position on scarp**	**m sample**	**m_cl spike[a]**	**36Cl/Cl[b]**	**± 1 sigma uncertainty**			**37Cl/35Cl**	**Clnat AMS**	**N_Cl36,rock**	
SUERC-	[cm]	[g]	[mg]	derived from 36Cl/35Cl		derived from 36Cl/37Cl			[ppm]	36Cl [atoms/g]	s36Cl [atoms/g]
c2508	-164	30.3305	1.3799	1.7568E-13	5.325E-15	8.818E-13	2.673E-14	0.0566	1.81	1.710E+05	6.94E+03
c2509	-134	30.0647	1.4131	1.765E-13	3.943E-15	9.0532E-13	2.023E-14	0.0553	1.51	1.722E+05	5.98E+03
c2510	-100	29.9853	1.4008	1.826E-13	5.21E-15	7.5647E-13	2.158E-14	0.0683	5.20	1.906E+05	7.26E+03
c2511	-28	30.2705	1.3738	2.1495E-13	6.313E-15	7.332E-13	2.153E-14	0.0829	9.64	2.400E+05	8.98E+03
c2512	-7	30.6107	1.4154	2.375E-13	6.673E-15	8.2854E-13	2.328E-14	0.0815	9.34	2.630E+05	9.52E+03
c2522	2	30.9558	1.4000	2.6313E-13	7.44E-15	1.1345E-12	3.208E-14	0.0653	4.17	2.640E+05	9.84E+03
c2517	36	30.6809	1.4054	2.9099E-13	8.234E-15	1.138E-12	3.22E-14	0.0720	6.21	3.054E+05	1.12E+04
c2533	36	30.5507	1.4239	3.054E-13	7.623E-15	1.388E-12	3.517E-14	0.0633	3.72	3.120E+05	1.09E+04
c2518	69	30.5181	1.4216	3.1419E-13	6.892E-15	1.2063E-12	2.646E-14	0.0734	6.76	3.350E+05	1.06E+04
c2519	103	30.1639	1.4085	3.6733E-13	9.098E-15	1.3373E-12	3.312E-14	0.0774	8.06	4.051E+05	1.35E+04
c2520	137	30.5243	1.3984	4.2663E-13	1.202E-14	1.7221E-12	4.85E-14	0.0698	5.55	4.477E+05	1.63E+04
c2521	154	30.5785	1.4193	4.5808E-13	1.134E-14	1.8678E-12	4.623E-14	0.0690	5.38	4.788E+05	1.61E+04

[a]Mass of $^{35/37}Cl$ spike added to sample prior to dissolution. Spike concentration: mgCl/g solution = 7.1777, $^{37}at/^{35}at$ = 0.0510.

[b]36Cl/Cl is based on either the measured $^{36}Cl/^{35}Cl$ or $^{36}Cl/^{37}Cl$ ratios assuming natural $^{35}Cl/^{37}Cl$ ratios.

Table 6.1.5: Cl content and chlorine isotopic composition of limestone fault samples

Fault: **Pescasseroli**
LOCATION 0400068E/4632244N 33T
ELEVATION 1304m
SHIELDING 0.673982134

**AMS ID**	**Z, position on scarp**	**m sample**	**m_cl spike**[a]	**36Cl/Cl**[b]	**± 1 sigma uncertainty**			**37Cl/35Cl**	**Clnat AMS**	**N_Cl36,rock**	
SUERC-	[cm]	[g]	[mg]	derived from	36Cl/35Cl	derived from	36Cl/37Cl		[ppm]	36Cl [atoms/g]	s36Cl [atoms/g]
c1776	-160	30.2178	1.1399	3.806E-13	9.439E-15	9.543E-13	2.367E-14	0.1154	18.63	400038	11621
c1777	-150	30.0789	1.0496	3.959E-13	8.747E-15	9.993E-13	2.208E-14	0.1147	16.98	405001	10864
c1778	-135	30.0722	1.0527	4.165E-13	7.691E-15	1.022E-12	2.679E-14	0.1185	18.41	437491	11779
c1779	-120	29.9864	1.0459	4.677E-13	1.122E-14	1.262E-12	3.030E-14	0.1072	14.44	459684	13143
c1780	-55	30.2712	1.0335	4.712E-13	1.139E-14	1.215E-12	2.937E-14	0.1122	15.77	470291	13463
c1787	-55	30.9329	1.0211	5.128E-13	1.437E-14	1.265E-12	3.545E-14	0.1174	16.98	514701	16412
c1781	-15	30.9013	1.0267	5.270E-13	1.127E-14	1.347E-12	2.881E-14	0.1132	15.67	517557	13581
c1786	0	29.6856	1.0063	5.446E-13	1.507E-14	1.444E-12	3.996E-14	0.1091	14.64	540852	17174
c1788	45	30.0530	1.0156	6.801E-13	1.624E-14	1.773E-12	4.234E-14	0.1110	15.21	677263	19208
c1789	125	30.7816	1.0310	8.672E-13	2.415E-14	2.091E-12	5.824E-14	0.1200	18.15	893665	28227
c1790	140	30.0091	1.0378	8.621E-13	2.345E-14	2.073E-12	5.641E-14	0.1203	18.85	914846	28365
c1791	270	29.5511	1.0007	9.477E-13	2.461E-14	2.354E-12	6.112E-14	0.1165	17.10	986999	29625
c1796	345	29.9064	1.0137	1.102E-12	3.057E-14	2.771E-12	7.691E-14	0.1151	16.63	1130354	35701
c1797	385	29.9760	1.0273	1.150E-12	2.426E-14	2.977E-12	6.280E-14	0.1118	15.69	1160326	30109

[a]Mass of $^{35/37}$Cl spike added to sample prior to dissolution. Spike concentration: mgCl/g solution = 6.1850, ^{37}at/^{35}at = 0.0510.

[b]36Cl/Cl is based on either the measured ^{36}Cl/^{35}Cl or ^{36}Cl/^{37}Cl ratios assuming natural ^{35}Cl/^{37}Cl ratios.

Table 6.1.6: Cl content and chlorine isotopic composition of limestone fault samples

Fault: **San Sebastiano**

LOCATION 0397401E/4644517N 33T

ELEVATION 1207m

SHIELDING 0.75281255

**AMS ID** SUERC-	**Z, position on scarp** [cm]	**m sample** [g]	**m_cl spike**[a] [mg]	**36Cl/Cl**[b] derived from 36Cl/35Cl	**± 1 sigma uncertainty**	derived from 36Cl/37Cl		**37Cl/35Cl**	**Clnat AMS** [ppm]	**N_Cl36,rock** 36Cl [atoms/g]	s36Cl [atoms/g]
c2565	-145	31.7039	1.3992	2.399E-13	7.015E-15	6.910E-13	2.043E-14	0.0999	15.80	280591	9565
c2566	-80	31.3005	1.3799	2.9045E-13	7.352E-15	1.0899E-12	2.801E-14	0.0767	7.74	302482	9462
c2567	-40	30.1799	1.3714	4.8593E-13	1.107E-14	2.4625E-12	5.716E-14	0.0568	2.22	476502	14137
c2568	0	30.7089	1.4177	3.7268E-13	1.092E-14	1.4042E-12	4.16E-14	0.0764	8.01	398206	13675
c2574	80	30.2701	1.3707	3.0356E-13	9.766E-15	1.3211E-12	4.89E-14	0.0662	4.79	309491	12747
c2575	120	30.8544	1.3637	6.2859E-13	2.236E-14	3.3444E-12	1.329E-13	0.0541	1.48	596102	25163
c2576	200	30.2094	1.3830	8.0599E-13	2.32E-14	4.2465E-12	1.237E-13	0.0547	1.68	785326	27033

[a]Mass of $^{35/37}$Cl spike added to sample prior to dissolution. Spike concentration: mgCl/g solution = 7.1777, ^{37}at/^{35}at = 0.0510.

[b]36Cl/Cl is based on either the measured ^{36}Cl/^{35}Cl or ^{36}Cl/^{37}Cl ratios assuming natural ^{35}Cl/^{37}Cl ratios.

Table 6.1.7: Cl content and chlorine isotopic composition of limestone fault samples

Fault: **Frattura**

LOCATION 0407445E/4642543N 33T

ELEVATION 1484 m

SHIELDING 0.969976117

**AMS ID**	**Z, position on scarp**	**m sample**	**m_cl spike[a]**	**36Cl/Cl[b]**	**± 1 sigma uncertainty**			**37Cl/35Cl**	**Clnat AMS**	**N_Cl36,rock**	
SUERC-	[cm]	[g]	[mg]	derived from 36Cl/35Cl		derived from 36Cl/37Cl			[ppm]	36Cl [atoms/g]	s36Cl [atoms/g]
c3002	-130	29.8943	1.0211	1.469E-13	4.468E-15	2.533E-13	7.370E-15	0.1754	44.97	219283	7192
c3003	-110	30.7973	1.0262	1.6008E-13	4.843E-15	2.7133E-13	7.849E-15	0.1784	46.07	238981	7674
c3004	-90	30.1275	1.0280	1.5547E-13	3.538E-15	2.545E-13	5.334E-15	0.1847	52.31	251143	7322
c3005	-60	30.4543	1.0167	1.7377E-13	5.263E-15	2.9518E-13	8.549E-15	0.1780	45.86	260319	8327
c3013	-30	29.9533	1.0331	1.7238E-13	4.299E-15	2.9075E-13	7.251E-15	0.1752	45.22	255743	7042
c3014	0	30.1117	1.0205	1.849E-13	5.28E-15	3.1733E-13	9.062E-15	0.1721	42.24	264267	9065
c3015	100	30.1540	1.0041	2.2041E-13	6.316E-15	3.473E-13	9.953E-15	0.1875	53.41	357111	10828
c3016	140	30.0433	1.0230	2.1068E-13	5.863E-15	3.317E-13	9.157E-15	0.1876	54.71	346671	11903
c3017	180	30.1950	1.0167	2.1725E-13	5.436E-15	3.4576E-13	8.651E-15	0.1856	52.34	347971	9336
c3022	280	30.2089	1.0224	2.5695E-13	7.367E-15	4.1385E-13	1.186E-14	0.1834	50.74	405238	12231
c3023	380	30.5002	0.9808	2.9337E-13	8.371E-15	4.5234E-13	1.291E-14	0.1915	55.12	481903	14439
c3024	480	30.6277	1.0230	3.4541E-13	9.801E-15	5.7503E-13	1.632E-14	0.1774	45.39	511120	15165
c3025	580	30.1401	1.0318	4.336E-13	1.213E-14	7.3608E-13	2.06E-14	0.1740	44.01	637652	18590
c2970	680	29.9436	1.0154	5.0056E-13	1.354E-14	7.9556E-13	2.119E-14	0.1807	51.34	791302	24488
c2971	750	30.1518	1.0192	6.349E-13	1.898E-14	1.0581E-12	3.125E-14	0.1724	44.59	929615	30824

[a]Mass of $^{35/37}$Cl spike added to sample prior to dissolution. Spike concentration: mgCl/g solution = 6.2954, ^{37}at/^{35}at = 0.05008.

[b]36Cl/Cl is based on either the measured ^{36}Cl/^{35}Cl or ^{36}Cl/^{37}Cl ratios assuming natural ^{35}Cl/^{37}Cl ratios.

Table 6.1.8: Cl content and chlorine isotopic composition of limestone bedrock footwall samples

Sample: **Fiamignano Upper** (dip of planar surface: ~35deg. using compass clino; consistent with footwall dip)

LOCATION 0344611E/4681859N 33T

ELEVATION 1219m

SHIELDING 0.955987063

AMS ID SUERC-	Thickness [cm]	m sample [g]	m_Cl spike[a] [mg]	36Cl/Cl[b] derived from 36Cl/35Cl	± 1 sigma uncertainty	derived from 36Cl/37Cl		37Cl/35Cl	Clnat AMS [ppm]	N_Cl36,rock 36Cl [atoms/g]	s36Cl [atoms/g]
c1703	2.5	30.4275	0.9952	9.942E-13	2.878E-14	2.676E-12	7.746E-14	0.1074	13.77	897581	31497
c1704	2.5	30.0882	0.9785	1.004E-12	2.825E-14	2.706E-12	7.613E-14	0.1073	13.66	911641	31391
c1770	2.5	31.5759	0.9828	1.056E-12	2.805E-14	2.733E-12	7.257E-14	0.1119	14.11	970635	67870
c1800	2.5	30.1945	1.0317	9.725E-13	2.697E-14	2.658E-12	7.372E-14	0.1059	13.74	943037	29958
c1933	2.5	31.0887	1.0453	9.701E-13	2.602E-14	2.403E-12	6.444E-14	0.1148	12.59	963909	28151
c2477	2.5	30.2004	1.2066	9.099E-13	2.423E-14	2.609E-12	6.947E-14	0.1008	14.41	1030032	28311
c2501	2.5	30.8162	1.2697	8.220E-13	2.252E-14	2.435E-12	6.670E-14	0.0970	13.75	934793	26414
c1956	2.5	30.8757	1.0242	1.033E-12	2.417E-14	2.816E-12	6.587E-14	0.1043	12.83	947516	24393
c2193	2.5	30.0995	1.0768	9.348E-13	1.021E-14	2.684E-12	2.930E-14	0.1005	13.23	929397	12163
c2068	2.5	30.1065	1.1257	9.087E-13	1.823E-14	2.585E-12	5.186E-14	0.1009	13.01	896232	18243
c2093	2.5	31.4667	1.0490	1.055E-12	2.922E-14	2.873E-12	7.960E-14	0.1057	12.94	946807	27185

[a]Mass of $^{35/37}$Cl spike added to sample prior to dissolution. Spike concentration: for samples c1703 - c2193 the mgCl/g solution = 6.1850, ^{37}at/^{35}at = 0.0510; for samples c2477 and c2501 the mgCl/g solution = 7.1777, ^{37}at/^{35}at = 0.0510.

[b]36Cl/Cl is based on either the measured ^{36}Cl/^{35}Cl or ^{36}Cl/^{37}Cl ratios assuming natural ^{35}Cl/^{37}Cl ratios.

Table 6.1.9: Cl content and chlorine isotopic composition of processed blanks.

**AMS ID** SUERC -	**AMS ID for assoc. samples** (SUERC-)	**m sample** [g]	**m_cl spike**[a] [mg]	**spike Cl content** [mgCl/g solu'n]	**ratio spike Cl** ^{37}at/^{35}at	**36Cl/Cl**[b] derived from 36Cl/35Cl	**± 1 sigma uncertainty**	derived from 36Cl/37Cl		**37Cl/35Cl**
Fiamignano										
c1253	c1230 - c1252	0	0.8889	6.2602	0.0551	8.382E-15	6.891E-16	1.931E-14	1.593E-15	0.1264
c1254	c1230 - c1252	0	0.9253	6.2602	0.0551	8.1078E-15	2.118E-15	1.7888E-14	4.678E-15	0.1321
c1281	c1259 - c1280	0	0.9365	6.2602	0.0551	8.6951E-15	7.53E-16	1.8109E-14	1.573E-15	0.1399
c1282	c1259 - c1280	0	0.9359	6.2602	0.0551	8.5476E-15	8.462E-16	1.7556E-14	1.742E-15	0.1418
c1326	c1297 - c1325	0	0.7594	6.2602	0.0551	9.579E-15	2.324E-15	1.6595E-14	4.025E-15	0.1696
c1551	c1523 - c1549	0	1.3068	6.6199	0.0507	3.4104E-15	5.462E-16	1.9767E-14	3.166E-15	0.0492
c1701	c1679 - c1700	0	0.9395	6.1850	0.0510	4.1924E-15	4.81E-16	2.6614E-14	3.054E-15	0.0458
c1702	c1679 - c1700	0	0.9673	6.1850	0.0510	3.5575E-15	5.515E-16	2.1231E-14	3.284E-15	0.0484
c1730	c1709 - c1729	0	1.0465	6.1850	0.0510	2.7554E-15	4.539E-16	1.6311E-14	2.691E-15	0.0488
c1731	c1709 - c1729	0	1.0137	6.1850	0.0510	4.189E-15	3.434E-16	2.4907E-14	2.042E-15	0.0486
c1840	c1811 - c1839	0	0.9160	6.1850	0.0510	1.1399E-15	4.309E-16	6.9344E-15	2.621E-15	0.0474
c1841	c1811 - c1839	0	0.9989	6.1850	0.0510	1.2565E-15	6.616E-16	7.5639E-15	3.984E-15	0.0480
c1957	c1934 - c1955	0	1.0236	6.1850	0.0510	5.2127E-15	4.539E-16	3.1245E-14	2.72E-15	0.0474
c1958	c1934 - c1955	0	1.0323	6.1850	0.0510	4.7185E-15	5.31E-16	2.8268E-14	3.181E-15	0.0475
c2067	c2040 - c2061	0	1.1009	6.1850	0.0510	5.6297E-15	9.953E-16	3.3469E-14	5.917E-15	0.0483
c2091	c2069 - c2090	0	1.0088	6.1850	0.0510	5.8177E-15	1.013E-15	3.4752E-14	6.05E-15	0.0482
c2092	c2069 - c2090	0	1.0205	6.1850	0.0510	5.5021E-15	1.003E-15	3.2852E-14	6.062E-15	0.0489
c2191	c2165 - c2190	0	1.0799	6.1850	0.0510	4.6893E-15	4.668E-16	2.8922E-14	2.879E-15	0.0468
c2192	c2165 - c2190	0	1.0737	6.1850	0.0510	4.5224E-15	4.666E-16	2.7818E-14	2.87E-15	0.0469
Gioa di Marsi		0								
c1768	c1746 - c1767	0	0.9364	6.1850	0.0510	2.6631E-15	3.184E-16	1.5615E-14	1.867E-15	0.0493
c1769	c1746 - c1767	0	1.0564	6.1850	0.0510	2.6501E-15	4.248E-16	1.5675E-14	2.495E-15	0.0489
c2039	c1913 - c2038	0	1.0236	6.1850	0.0510	6.8641E-15	9.034E-16	4.0382E-14	5.331E-15	0.0497
c1932	c1913 - c2038	0	1.0607	6.1850	0.0510	5.0165E-15	6.04E-16	2.1289E-14	2.563E-15	0.0670
c2478	c2338 - c2472	0	1.1233	7.1777	0.0510	7.8872E-15	1.179E-15	4.6488E-14	6.9E-15	0.0491
c2350	c2338 - c2472	0	1.2425	7.1777	0.0510	4.0826E-15	5.321E-16	2.4649E-14	3.214E-15	0.0481
c2502	c2479 - c2500	0	1.3049	7.1777	0.0510	5.3666E-15	4.143E-16	3.1957E-14	2.467E-15	0.0482
c2507	c2479 - c2500	0	1.2949	7.1777	0.0510	5.6064E-15	4.997E-16	3.3488E-14	2.985E-15	0.0477
Parasano		0								
c2584	c2534 - c2556	0	1.4100	7.1777	0.0510	3.6925E-15	4.756E-16	2.1707E-14	2.772E-15	0.0489
c2585	c2534 - c2556	0	1.3938	7.1777	0.0510	4.3651E-15	3.439E-16	2.5347E-14	2E-15	0.0496
c2586	c2557 - c2564	0	1.3876	7.1777	0.0510	7.8257E-15	7.377E-16	4.6725E-14	4.409E-15	0.0482
c2587	c2557 - c2564	0	1.3514	7.1777	0.0510	4.137E-15	9.278E-16	2.4853E-14	5.615E-15	0.0479
Tre Monte		0								

c2582	c2508 - c2538	0	1.3575	7.1777	0.0510	7.5939E-15	9.448E-16	4.324E-14	5.351E-15	0.0505
c2583	c2508 - c2538	0	1.4046	7.1777	0.0510	4.5205E-15	7.29E-16	2.6752E-14	4.316E-15	0.0486
Pescasseroli		0								
c1798	c1776 - c1797	0	1.0261	6.1850	0.0510	5.2321E-15	9.916E-16	3.1445E-14	5.89E-15	0.0480
c1799	c1776 - c1797	0	1.0552	6.1850	0.0510	5.4408E-15	6.75E-16	3.2723E-14	4.06E-15	0.0481
San Seb.		0								
c2586	c2565 - c2576	0	1.3876	7.1777	0.0510	7.8257E-15	7.377E-16	4.6725E-14	4.409E-15	0.0482
c2587	c2565 - c2576	0	1.3514	7.1777	0.0510	4.137E-15	9.278E-16	2.4853E-14	5.615E-15	0.0479
Frattura		0								
c2968	c2970 - c2971	0	1.0217	6.2954	0.0501	8.0003E-15	1.073E-15	4.632E-14	6.227E-15	0.0496
c2969	c2970 - c2971	0	1.0249	6.2954	0.0501	4.5785E-15	9.324E-16	2.7262E-14	5.558E-15	0.0483
c2978	c3002 - c3025	0	1.0350	6.2954	0.0501	5.1818E-15	6.501E-16	3.1108E-14	3.893E-15	0.0503
c2979	c3002 - c3025	0	1.0280	6.2954	0.0501	4.5878E-15	6.64E-16	2.7142E-14	3.921E-15	0.0511
FIAM - Us [c]		0								
c1701	c1703-c1704	0	0.9395	6.1850	0.0510	4.1924E-15	4.81E-16	2.6614E-14	3.054E-15	0.0458
c1702	c1703-c1704	0	0.9673	6.1850	0.0510	3.5575E-15	5.515E-16	2.1231E-14	3.284E-15	0.0484
c1768	c1770	0	0.9364	6.1850	0.0510	2.6631E-15	3.184E-16	1.5615E-14	1.867E-15	0.0493
c1769	c1770	0	1.0564	6.1850	0.0510	2.6501E-15	4.248E-16	1.5675E-14	2.495E-15	0.0489
c1798	c1800	0	1.0261	6.1850	0.0510	5.2321E-15	9.916E-16	3.1445E-14	5.89E-15	0.0480
c1799	c1800	0	1.0552	6.1850	0.0510	5.4408E-15	6.75E-16	3.2723E-14	4.06E-15	0.0481
c2039	c1933	0	1.0236	6.1850	0.0510	6.8641E-15	9.034E-16	4.0382E-14	5.331E-15	0.0497
c1932	c1933	0	1.0607	6.1850	0.0510	5.0165E-15	6.04E-16	2.1289E-14	2.563E-15	0.0670
c2478	c2477	0	1.1233	7.1777	0.0510	7.8872E-15	1.179E-15	4.6488E-14	6.9E-15	0.0491
c2350	c2477	0	1.2425	7.1777	0.0510	4.0826E-15	5.321E-16	2.4649E-14	3.214E-15	0.0481
c2502	c2501	0	1.3049	7.1777	0.0510	5.3666E-15	4.143E-16	3.1957E-14	2.467E-15	0.0482
c2507	c2501	0	1.2949	7.1777	0.0510	5.6064E-15	4.997E-16	3.3488E-14	2.985E-15	0.0477
c1957	c1956	0	1.0236	6.1850	0.0510	5.2127E-15	4.539E-16	3.1245E-14	2.72E-15	0.0474
c1958	c1956	0	1.0323	6.1850	0.0510	4.7185E-15	5.31E-16	2.8268E-14	3.181E-15	0.0475
c2191	c2193	0	1.0799	6.1850	0.0510	4.6893E-15	4.668E-16	2.8922E-14	2.879E-15	0.0468
c2192	c2193	0	1.0737	6.1850	0.0510	4.5224E-15	4.666E-16	2.7818E-14	2.87E-15	0.0469
c2067	c2068	0	1.1009	6.1850	0.0510	5.6297E-15	9.953E-16	3.3469E-14	5.917E-15	0.0483
c2091	c2093	0	1.0088	6.1850	0.0510	5.8177E-15	1.013E-15	3.4752E-14	6.05E-15	0.0482
c2092	c2093	0	1.0205	6.1850	0.0510	5.5021E-15	1.003E-15	3.2852E-14	6.062E-15	0.0489

[a]Mass of $^{35/37}$Cl spike added to blank. Spike concentrationn is listed for each processed blank.

[b]36Cl/Cl is based on either the measured ^{36}Cl/^{35}Cl or ^{36}Cl/^{37}Cl ratios assuming natural ^{35}Cl/^{37}Cl ratios.

[c]The Fiamignano upper slope samples were processed alonside multiple batches. Processed blanks are the same for those batches, and are repeated here for clarity.
